# Supplementary material for: Insight into the ecology of vaginal bacteria through integrative analyses of metagenomic and metatranscriptomic data
Source: Genome Biol. 2022 Mar 1;23:66. doi: 10.1186/s13059-022-02635-9 (PMC8886902; doi:10.1186/s13059-022-02635-9)
Supplement: Supplementary file 1 — Additional file 1. Plots that displaying the longitudinal taxonomic composition of the metagenome and metatranscriptome for each subject. [file 13059_2022_2635_MOESM1_ESM.pdf]

Supplementary File 1

Plots displaying the longitudinal taxonomic composition of the metagenome and metatranscriptome for each of the 39 subjects.

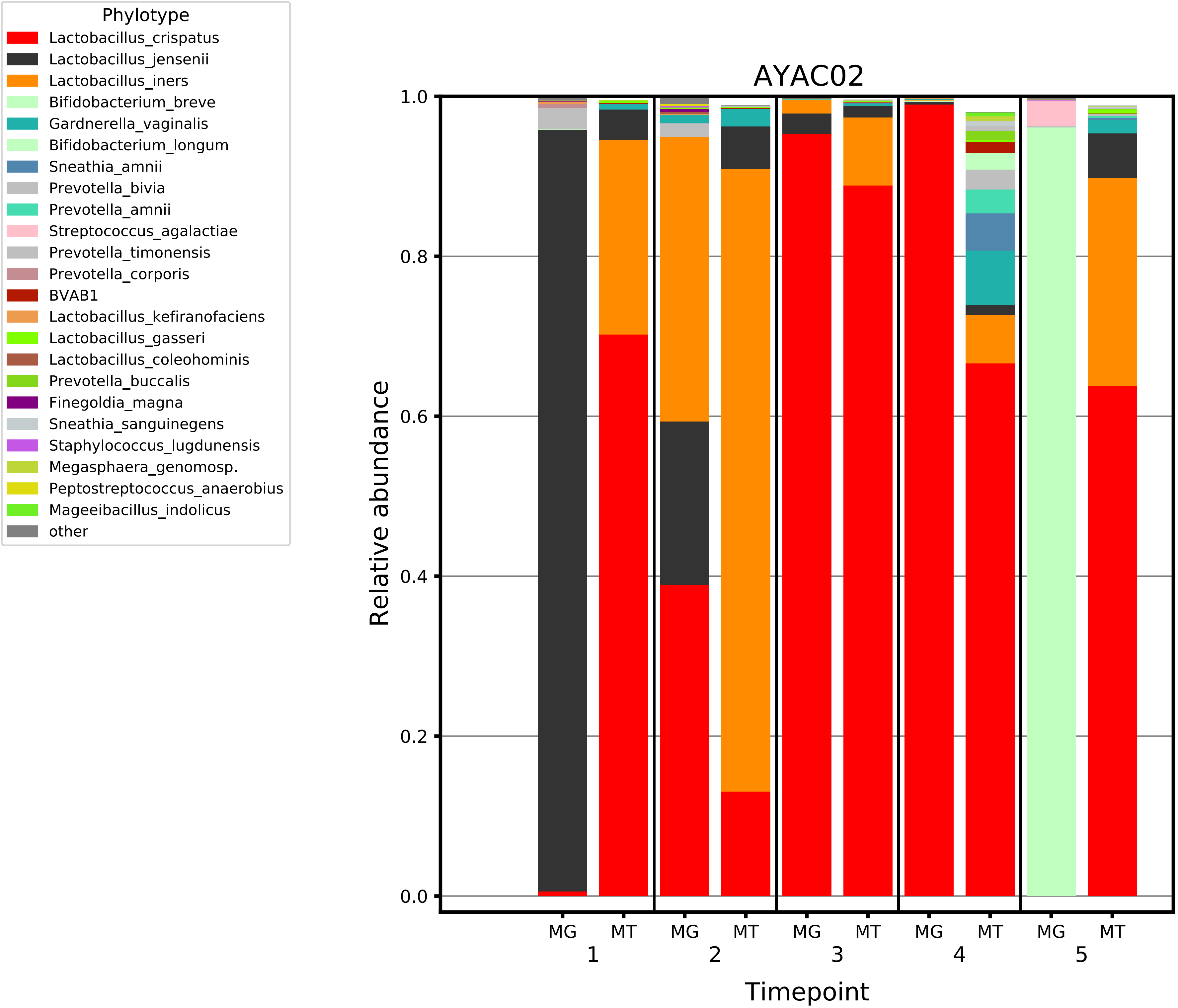

Phylotype
Lactobacillus_crispatus
Lactobacillus_jensenii
Lactobacillus_iners
Bifidobacterium_breve
Gardnerella_vaginalis
Bifidobacterium_longum
Sneathia_amnii
Prevotella_bivia
Prevotella_amnii
Streptococcus_agalactiae
Prevotella_timonensis
Prevotella_corporis
BVAB1
Lactobacillus_kefiranofaciens
Lactobacillus_gasseri
Lactobacillus_coleohominis
Prevotella_buccalis
Finegoldia_magna
Sneathia_sanguinegens
Staphylococcus_lugdunensis
Megasphaera_genomosp.
Peptostreptococcus_anaerobius
Mageeibacillus_indolicus
other
AYAC02
Relative abundance
1.0
0.8
0.6
0.4
0.2
0.0
MG
MT
1
2
3
4
5
Timepoint

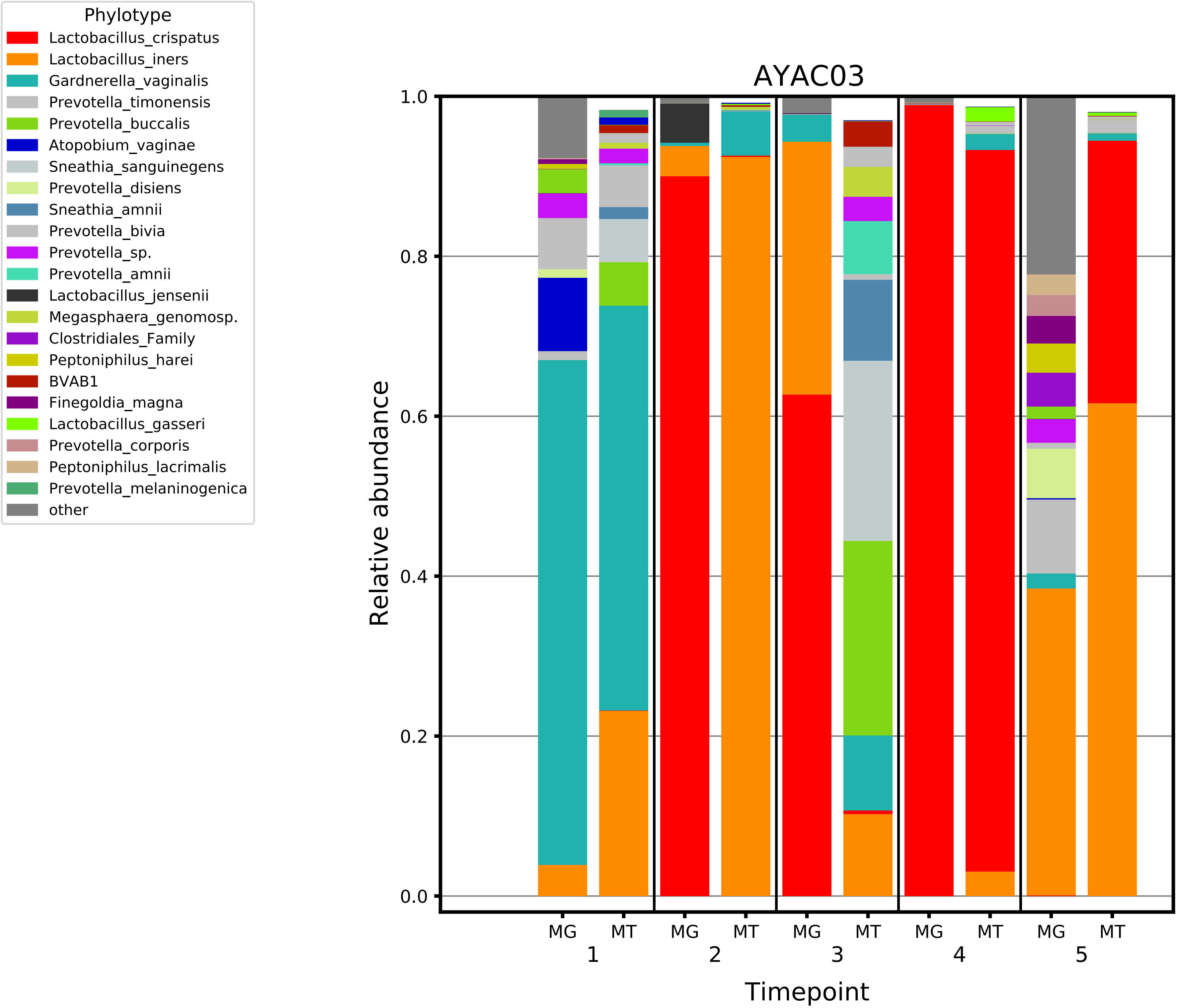

Phylotype
Lactobacillus_crispatus
Lactobacillus_iners
Gardnerella_vaginalis
Prevotella_timonensis
Prevotella_buccalis
Atopobium_vaginae
Sneathia_sanguinegens
Prevotella_disiens
Sneathia_amnii
Prevotella_bivia
Prevotella_sp.
Prevotella_amnii
Lactobacillus_jensenii
Megasphaera_genomosp.
Clostridiales_Family
Peptoniphilus_harei
BVAB1
Finegoldia_magna
Lactobacillus_gasseri
Prevotella_corporis
Peptoniphilus_lacrimalis
Prevotella_melaninogenica
other
AYAC03
Relative abundance
1.0
0.8
0.6
0.4
0.2
0.0
MG
MT
1
2
3
4
5
Timepoint

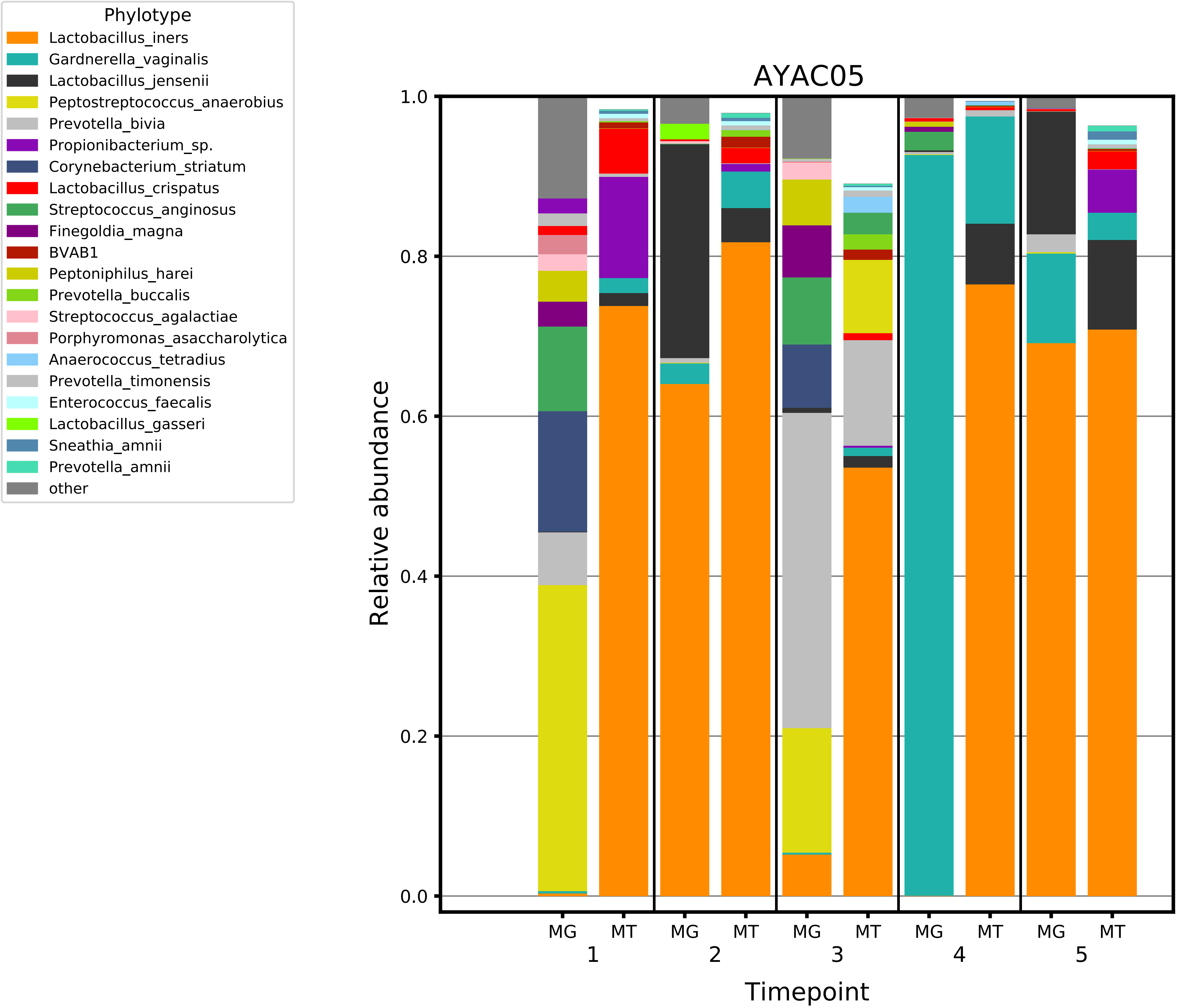
Phylotype
Lactobacillus_iners
Gardnerella_vaginalis
Lactobacillus_jensenii
Peptostreptococcus_anaerobius
Prevotella_bivia
Propionibacterium_sp.
Corynebacterium_striatum
Lactobacillus_crispatus
Streptococcus_anginosus
Finegoldia_magna
BVAB1
Peptoniphilus_harei
Prevotella_buccalis
Streptococcus_agalactiae
Porphyromonas_asaccharolytica
Anaerococcus_tetradius
Prevotella_timonensis
Enterococcus_faecalis
Lactobacillus_gasseri
Sneathia_amnii
Prevotella_amnii
other
AYAC05
Relative abundance
1.0
0.8
0.6
0.4
0.2
0.0
MG
MT
1
2
3
4
5
Timepoint

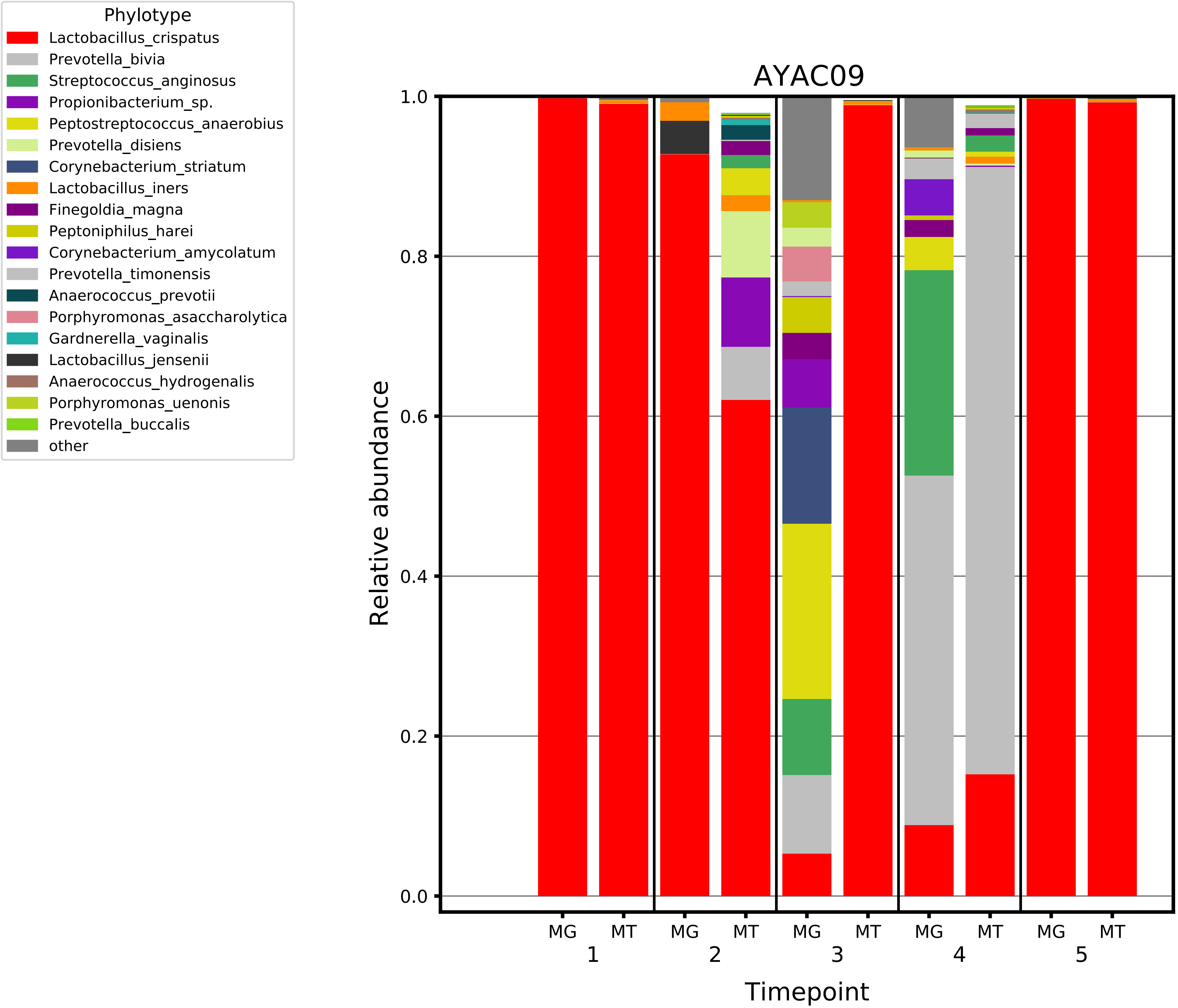
Phylotype
Lactobacillus_crispatus
Prevotella_bivia
Streptococcus_anginosus
Propionibacterium_sp.
Peptostreptococcus_anaerobius
Prevotella_disiens
Corynebacterium_striatum
Lactobacillus_iners
Finegoldia_magna
Peptoniphilus_harei
Corynebacterium_amycolatum
Prevotella_timonensis
Anaerococcus_prevotii
Porphyromonas_asaccharolytica
Gardnerella_vaginalis
Lactobacillus_jensenii
Anaerococcus_hydrogenalis
Porphyromonas_uenonis
Prevotella_buccalis
other
AYAC09
Relative abundance
1.0
0.8
0.6
0.4
0.2
0.0
MG
MT
1
2
3
4
5
Timepoint

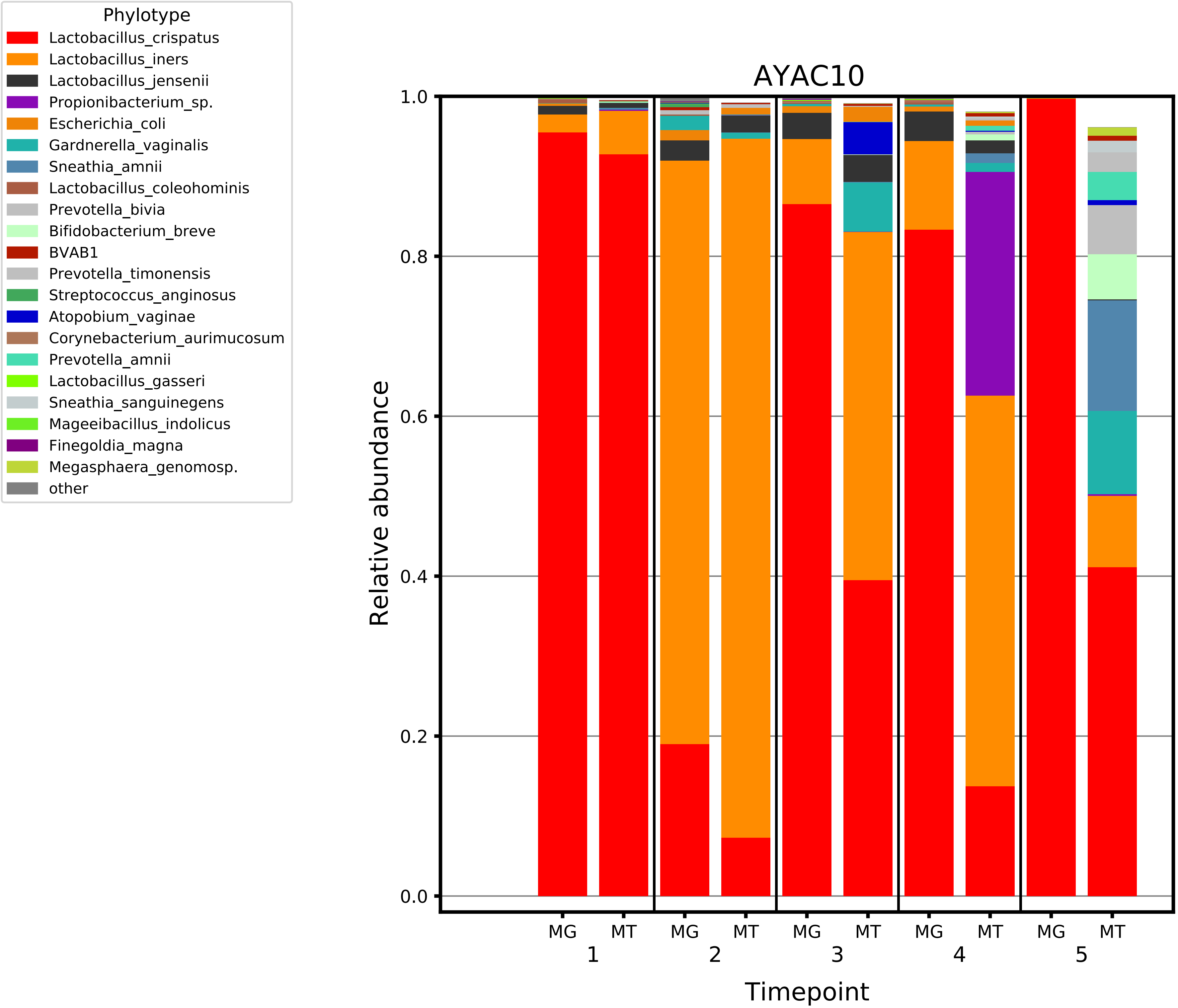
Phylotype
Lactobacillus_crispatus
Lactobacillus_iners
Lactobacillus_jensenii
Propionibacterium_sp.
Escherichia_coli
Gardnerella_vaginalis
Sneathia_amnii
Lactobacillus_coleohominis
Prevotella_bivia
Bifidobacterium_breve
BVAB1
Prevotella_timonensis
Streptococcus_anginosus
Atopobium_vaginae
Corynebacterium_aurimucosum
Prevotella_amnii
Lactobacillus_gasseri
Sneathia_sanguinegens
Mageeibacillus_indolicus
Finegoldia_magna
Megasphaera_genomosp.
other
AYAC10
Relative abundance
0.0
0.2
0.4
0.6
0.8
1.0
MG
MT
1
2
3
4
5
Timepoint

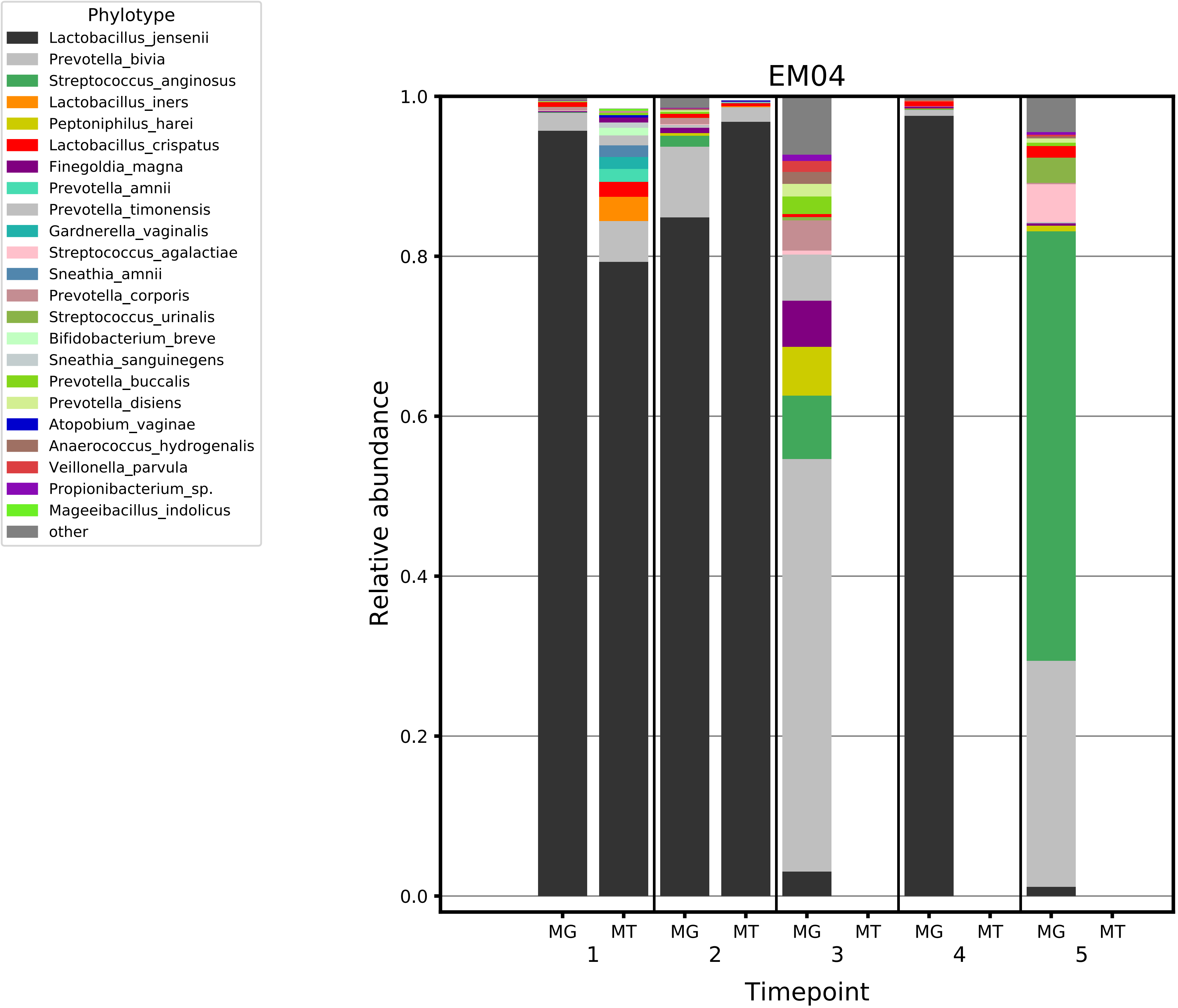

Phylotype
Lactobacillus_jensenii
Prevotella_bivia
Streptococcus_anginosus
Lactobacillus_iners
Peptoniphilus_harei
Lactobacillus_crispatus
Finegoldia_magna
Prevotella_amnii
Prevotella_timonensis
Gardnerella_vaginalis
Streptococcus_agalactiae
Sneathia_amnii
Prevotella_corporis
Streptococcus_urinalis
Bifidobacterium_breve
Sneathia_sanguinegens
Prevotella_buccalis
Prevotella_disiens
Atopobium_vaginae
Anaerococcus_hydrogenalis
Veillonella_parvula
Propionibacterium_sp.
Mageeibacillus_indolicus
other
EM04
Relative abundance
1.0
0.8
0.6
0.4
0.2
0.0
MG
MT
1
2
3
4
5
Timepoint

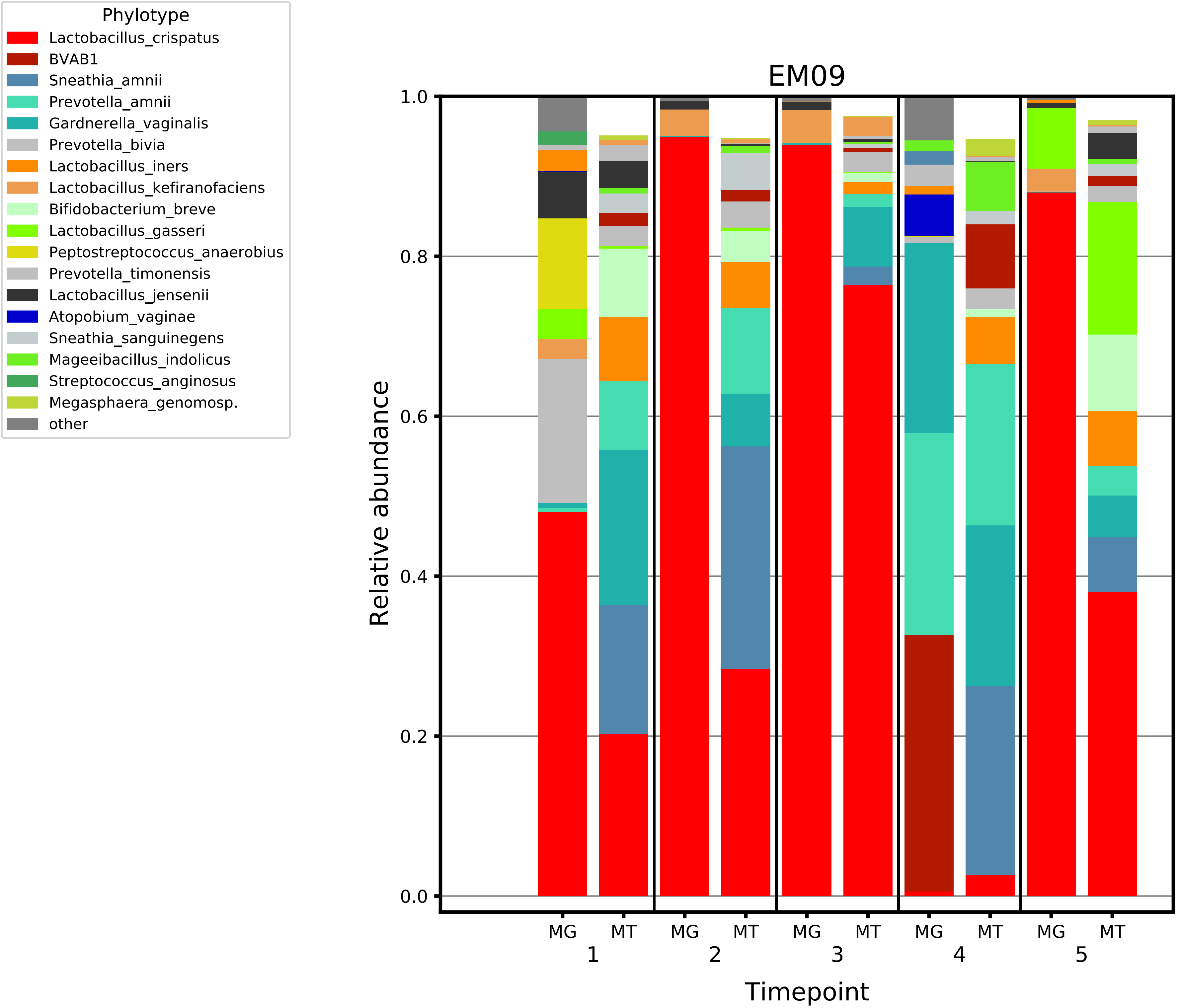

Phylotype
Lactobacillus_crispatus
BVAB1
Sneathia_amnii
Prevotella_amnii
Gardnerella_vaginalis
Prevotella_bivia
Lactobacillus_iners
Lactobacillus_kefiranofaciens
Bifidobacterium_breve
Lactobacillus_gasseri
Peptostreptococcus_anaerobius
Prevotella_timonensis
Lactobacillus_jensenii
Atopobium_vaginae
Sneathia_sanguinegens
Mageeibacillus_indolicus
Streptococcus_anginosus
Megasphaera_genomosp.
other
EM09
Relative abundance
1.0
0.8
0.6
0.4
0.2
0.0
MG
MT
1
2
3
4
5
Timepoint

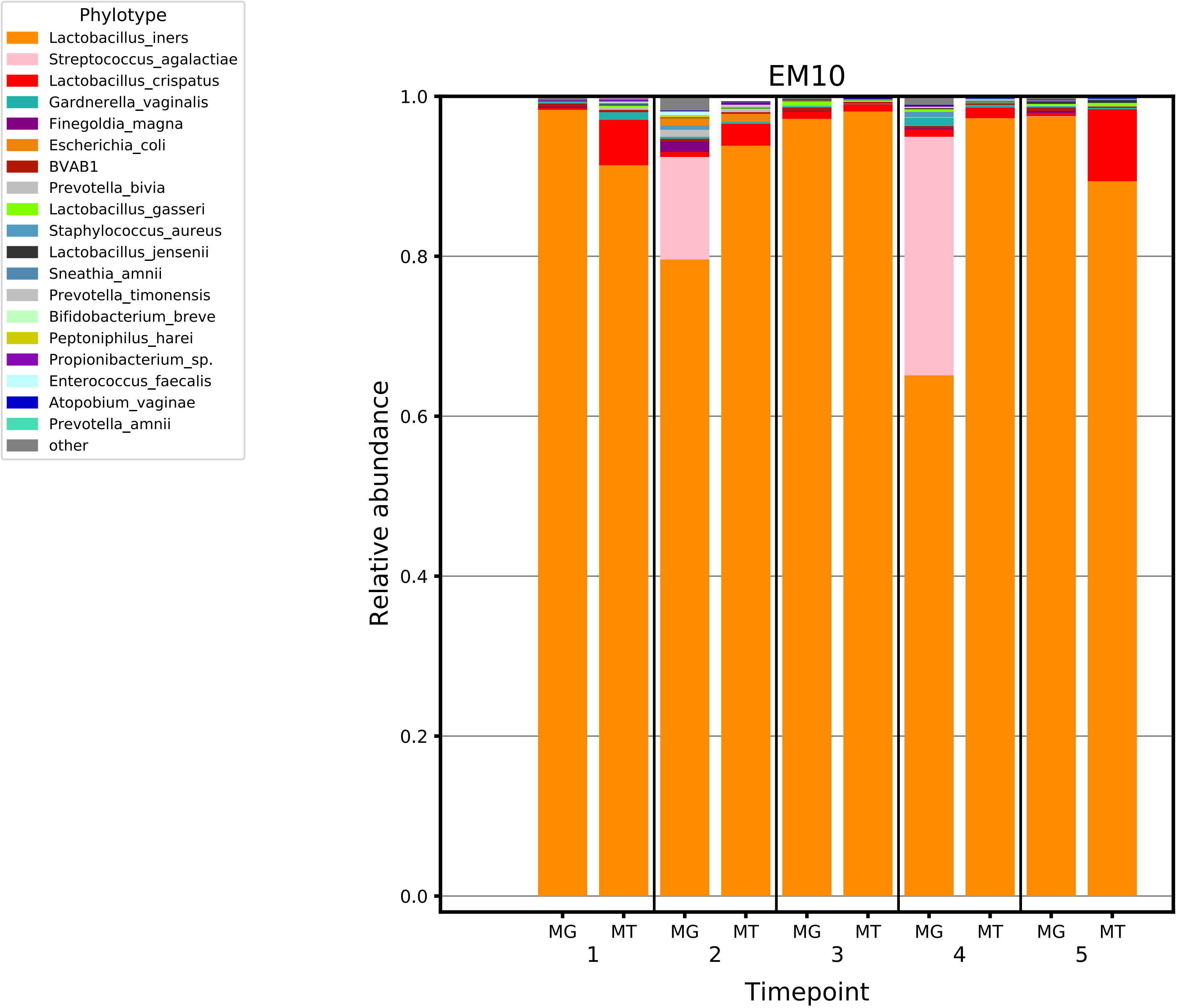
Phylotype
Lactobacillus_iners
Streptococcus_agalactiae
Lactobacillus_crispatus
Gardnerella_vaginalis
Finegoldia_magna
Escherichia_coli
BVAB1
Prevotella_bivia
Lactobacillus_gasseri
Staphylococcus_aureus
Lactobacillus_jensenii
Sneathia_amnii
Prevotella_timonensis
Bifidobacterium_breve
Peptoniphilus_harei
Propionibacterium_sp.
Enterococcus_faecalis
Atopobium_vaginae
Prevotella_amnii
other
EM10
Relative abundance
1.0
0.8
0.6
0.4
0.2
0.0
MG MT MG MT MG MT MG MT MG MT
1 2 3 4 5
Timepoint

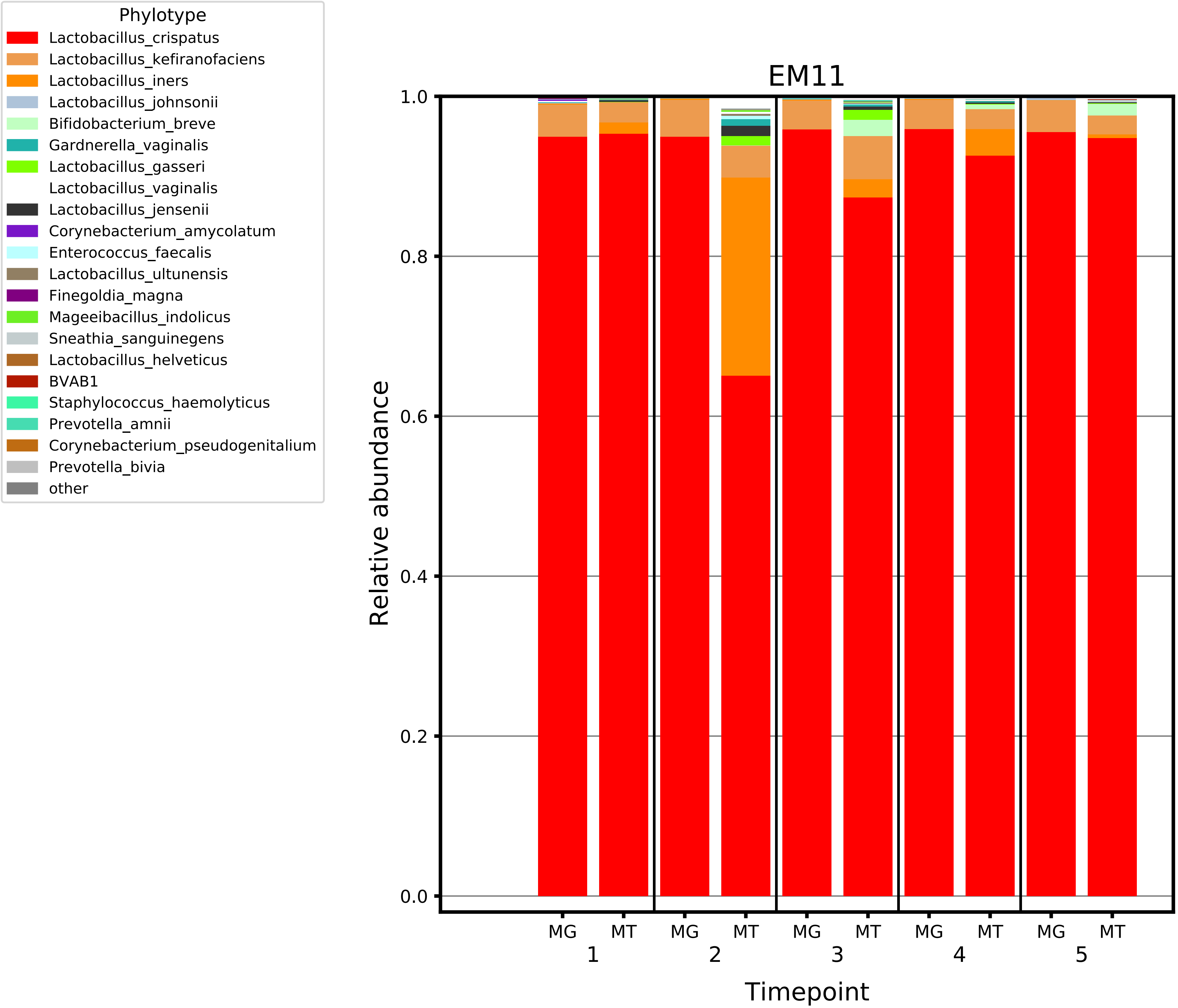
Phylotype
Lactobacillus_crispatus
Lactobacillus_kefiranofaciens
Lactobacillus_iners
Lactobacillus_johnsonii
Bifidobacterium_breve
Gardnerella_vaginalis
Lactobacillus_gasseri
Lactobacillus_vaginalis
Lactobacillus_jensenii
Corynebacterium_amycolatum
Enterococcus_faecalis
Lactobacillus_ultunensis
Finegoldia_magna
Mageeibacillus_indolicus
Sneathia_sanguinegens
Lactobacillus_helveticus
BVAB1
Staphylococcus_haemolyticus
Prevotella_amnii
Corynebacterium_pseudogenitalium
Prevotella_bivia
other
EM11
Relative abundance
1.0
0.8
0.6
0.4
0.2
0.0
MG
MT
1
2
3
4
5
Timepoint

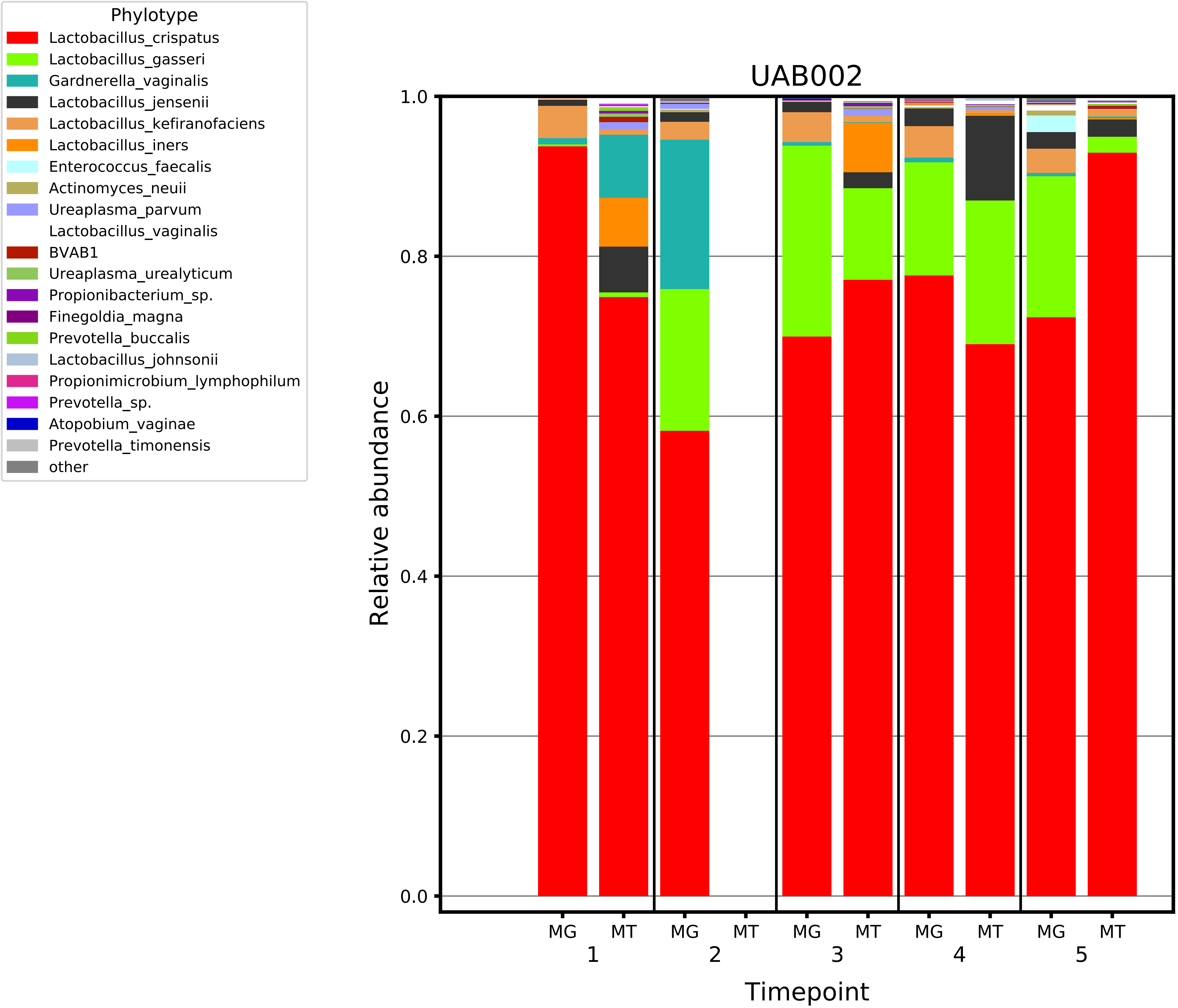
Phylotype
Lactobacillus_crispatus
Lactobacillus_gasseri
Gardnerella_vaginalis
Lactobacillus_jensenii
Lactobacillus_kefiranofaciens
Lactobacillus_iners
Enterococcus_faecalis
Actinomyces_neuii
Ureaplasma_parvum
Lactobacillus_vaginalis
BVAB1
Ureaplasma_urealyticum
Propionibacterium_sp.
Finegoldia_magna
Prevotella_buccalis
Lactobacillus_johnsonii
Propionimicrobium_lymphophilum
Prevotella_sp.
Atopobium_vaginae
Prevotella_timonensis
other
UAB002
Relative abundance
1.0
0.8
0.6
0.4
0.2
0.0
MG
MT
1
2
3
4
5
Timepoint

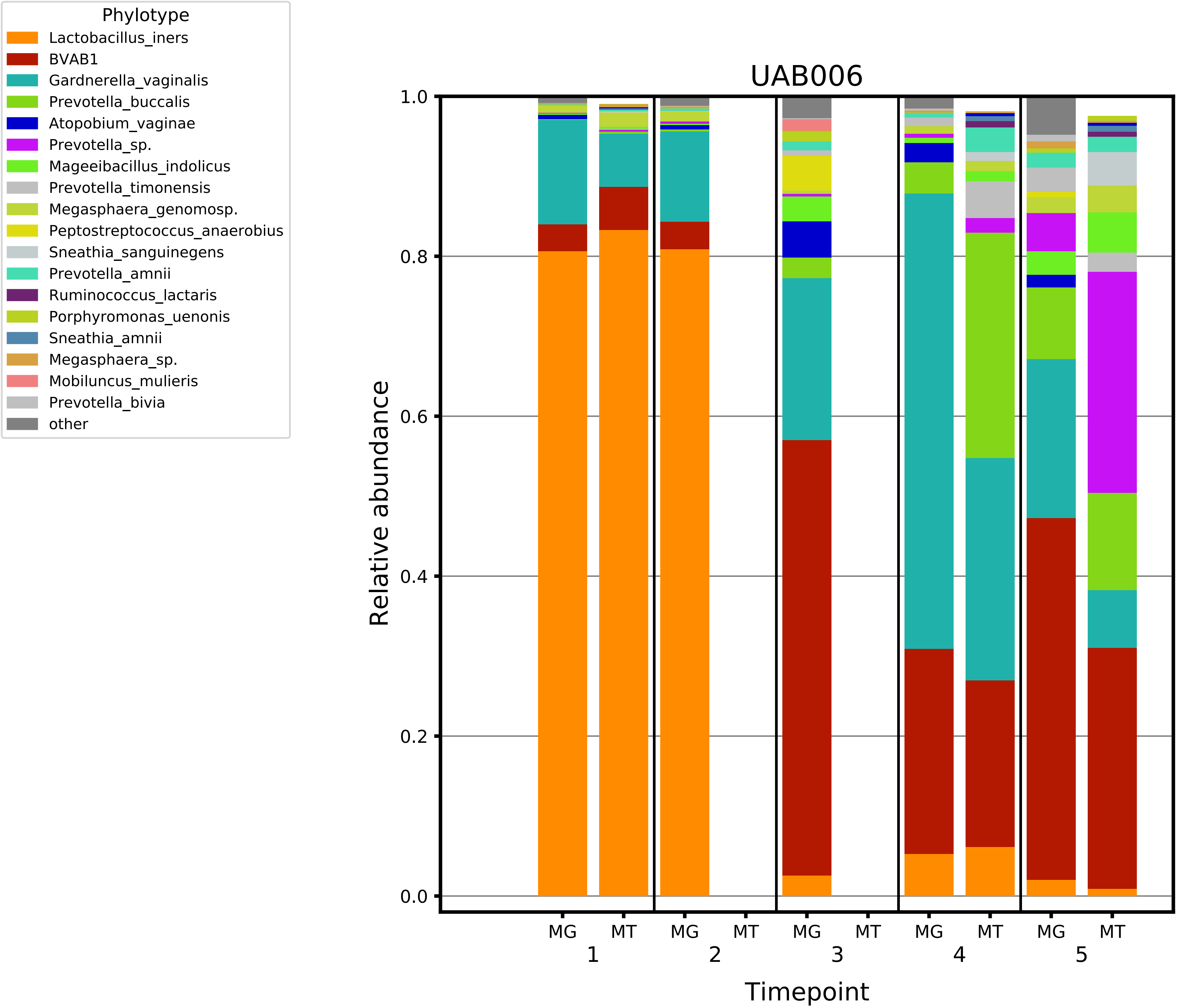

Phylotype
Lactobacillus_iners
BVAB1
Gardnerella_vaginalis
Prevotella_buccalis
Atopobium_vaginae
Prevotella_sp.
Mageeibacillus_indolicus
Prevotella_timonensis
Megasphaera_genomosp.
Peptostreptococcus_anaerobius
Sneathia_sanguinegens
Prevotella_amnii
Ruminococcus_lactaris
Porphyromonas_uenonis
Sneathia_amnii
Megasphaera_sp.
Mobiluncus_mulieris
Prevotella_bivia
other
UAB006
Relative abundance
1.0
0.8
0.6
0.4
0.2
0.0
MG
MT
1
2
3
4
5
Timepoint

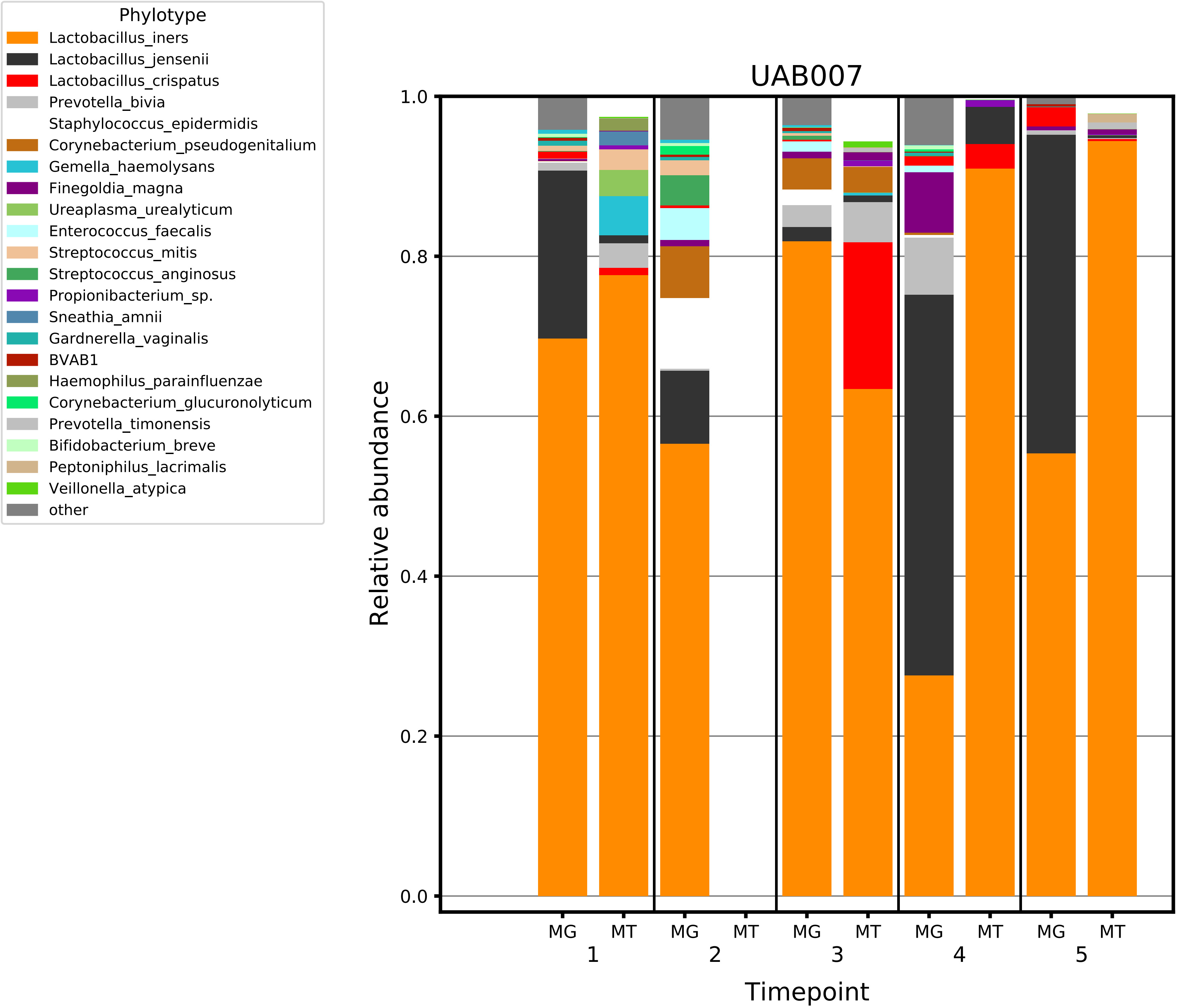

Phylotype
Lactobacillus_iners
Lactobacillus_jensenii
Lactobacillus_crispatus
Prevotella_bivia
Staphylococcus_epidermidis
Corynebacterium_pseudogenitalium
Gemella_haemolysans
Finegoldia_magna
Ureaplasma_urealyticum
Enterococcus_faecalis
Streptococcus_mitis
Streptococcus_anginosus
Propionibacterium_sp.
Sneathia_amnii
Gardnerella_vaginalis
BVAB1
Haemophilus_parainfluenzae
Corynebacterium_glucuronolyticum
Prevotella_timonensis
Bifidobacterium_breve
Peptoniphilus_lacrimalis
Veillonella_atypica
other
UAB007
Relative abundance
1.0
0.8
0.6
0.4
0.2
0.0
MG
MT
1
2
3
4
5
Timepoint

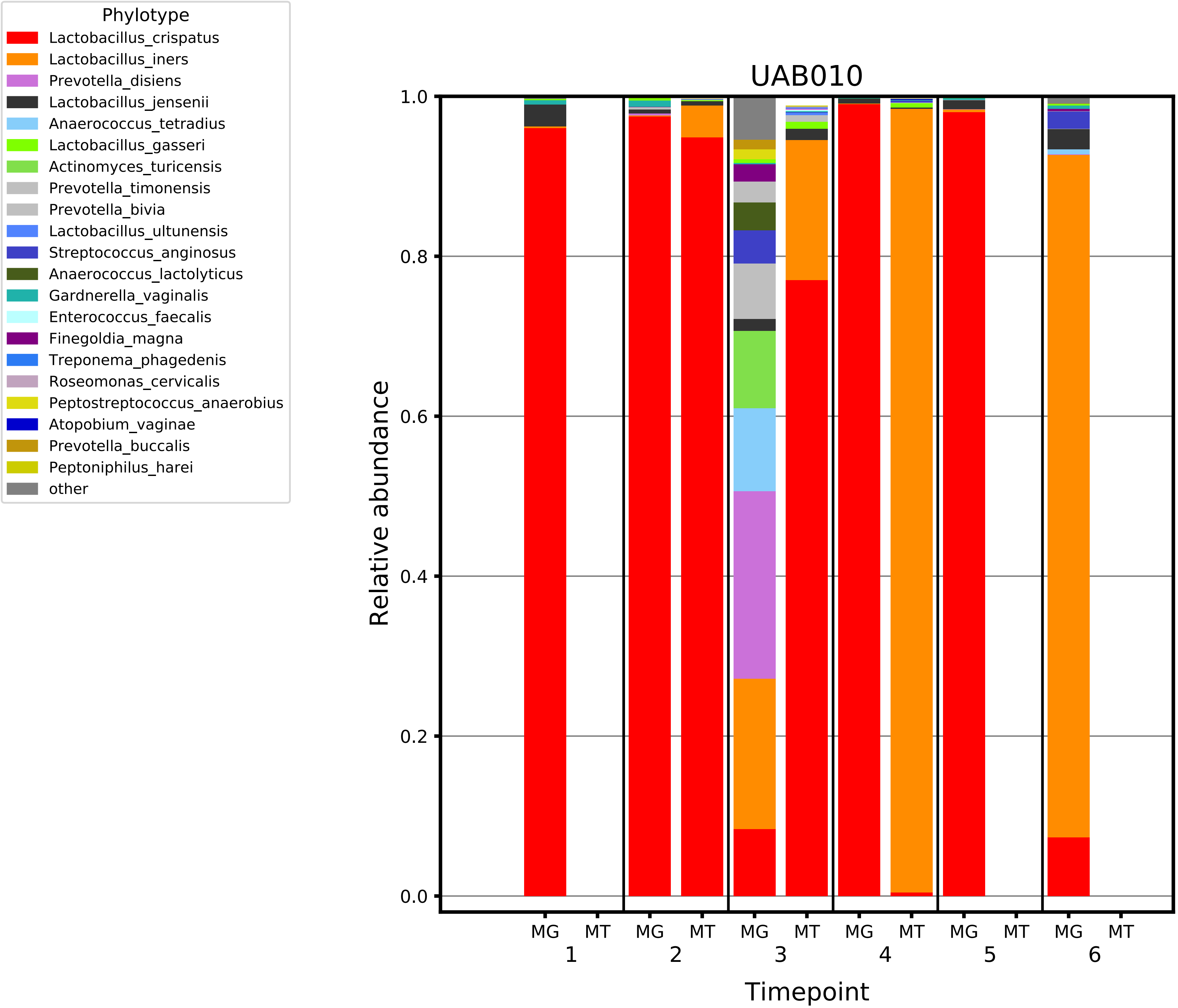

Phylotype
Lactobacillus_crispatus
Lactobacillus_iners
Prevotella_disiens
Lactobacillus_jensenii
Anaerococcus_tetradius
Lactobacillus_gasseri
Actinomyces_turicensis
Prevotella_timonensis
Prevotella_bivia
Lactobacillus_ultunensis
Streptococcus_anginosus
Anaerococcus_lactolyticus
Gardnerella_vaginalis
Enterococcus_faecalis
Finegoldia_magna
Treponema_phagedenis
Roseomonas_cervicalis
Peptostreptococcus_anaerobius
Atopobium_vaginae
Prevotella_buccalis
Peptoniphilus_harei
other
UAB010
Relative abundance
1.0
0.8
0.6
0.4
0.2
0.0
MG MT MG MT MG MT MG MT MG MT MG MT
1 2 3 4 5 6
Timepoint

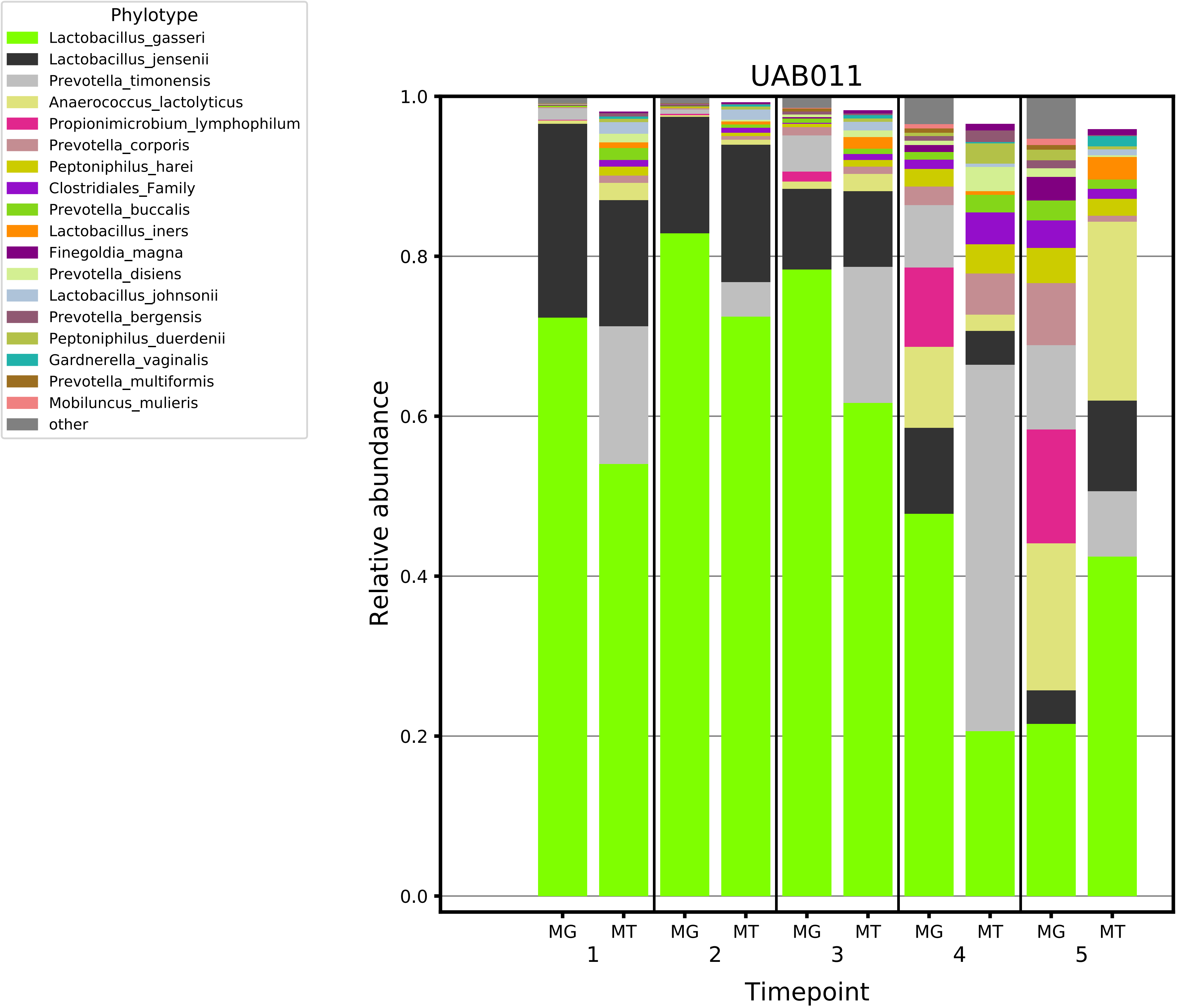

Phylotype
Lactobacillus_gasseri
Lactobacillus_jensenii
Prevotella_timonensis
Anaerococcus_lactolyticus
Propionimicrobium_lymphophilum
Prevotella_corporis
Peptoniphilus_harei
Clostridiales_Family
Prevotella_buccalis
Lactobacillus_iners
Finegoldia_magna
Prevotella_disiens
Lactobacillus_johnsonii
Prevotella_bergensis
Peptoniphilus_duerdenii
Gardnerella_vaginalis
Prevotella_multiformis
Mobiluncus_mulieris
other
UAB011
Relative abundance
1.0
0.8
0.6
0.4
0.2
0.0
MG
MT
1
2
3
4
5
Timepoint

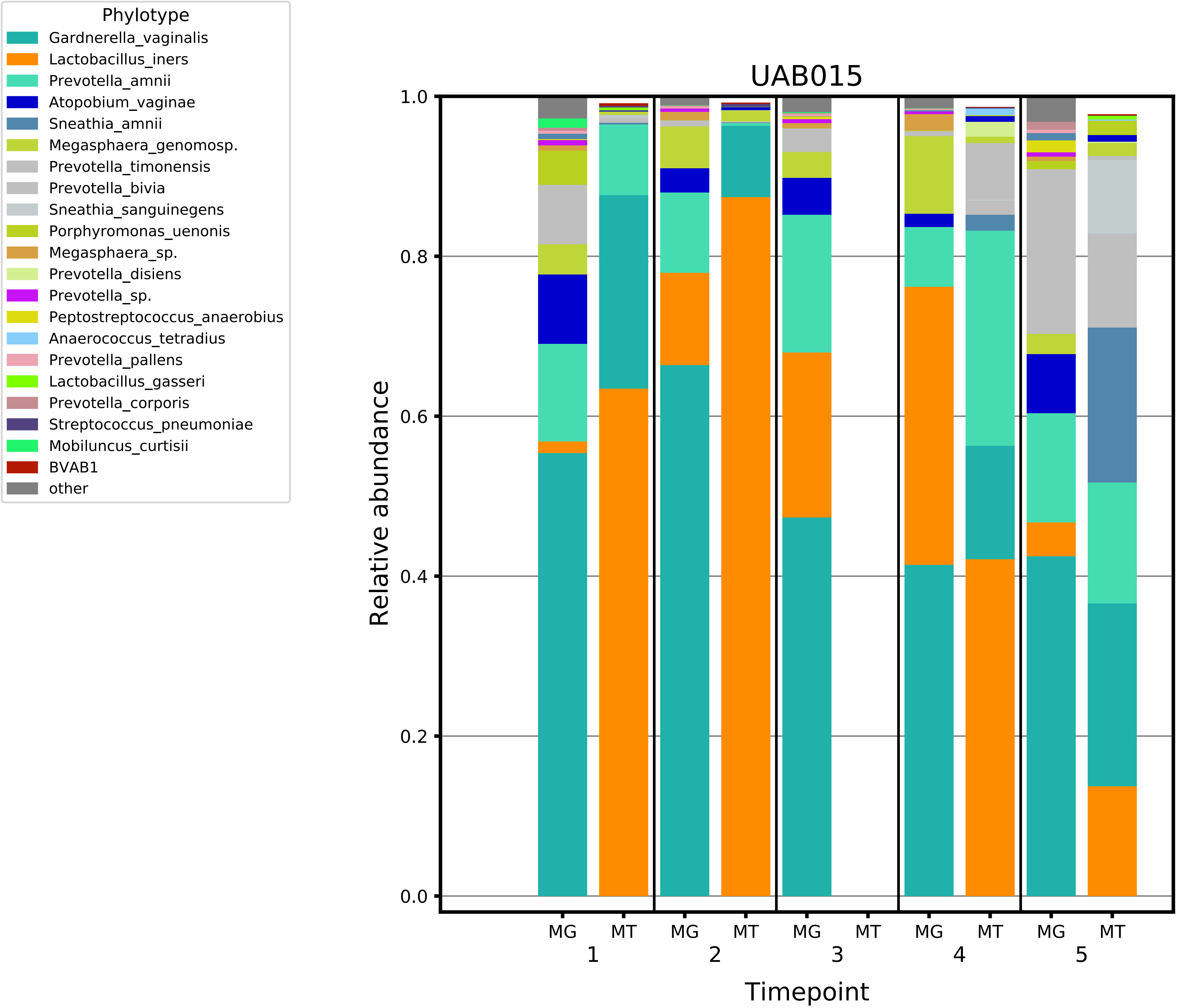
Phylotype
Gardnerella_vaginalis
Lactobacillus_iners
Prevotella_amnii
Atopobium_vaginae
Sneathia_amnii
Megasphaera_genomosp.
Prevotella_timonensis
Prevotella_bivia
Sneathia_sanguinegens
Porphyromonas_uenonis
Megasphaera_sp.
Prevotella_disiens
Prevotella_sp.
Peptostreptococcus_anaerobius
Anaerococcus_tetradius
Prevotella_pallens
Lactobacillus_gasseri
Prevotella_corporis
Streptococcus_pneumoniae
Mobiluncus_curtisii
BVAB1
other
UAB015
1.0
0.8
0.6
0.4
0.2
0.0
Relative abundance
MG
MT
MG
MT
MG
MT
MG
MT
MG
MT
1
2
3
4
5
Timepoint

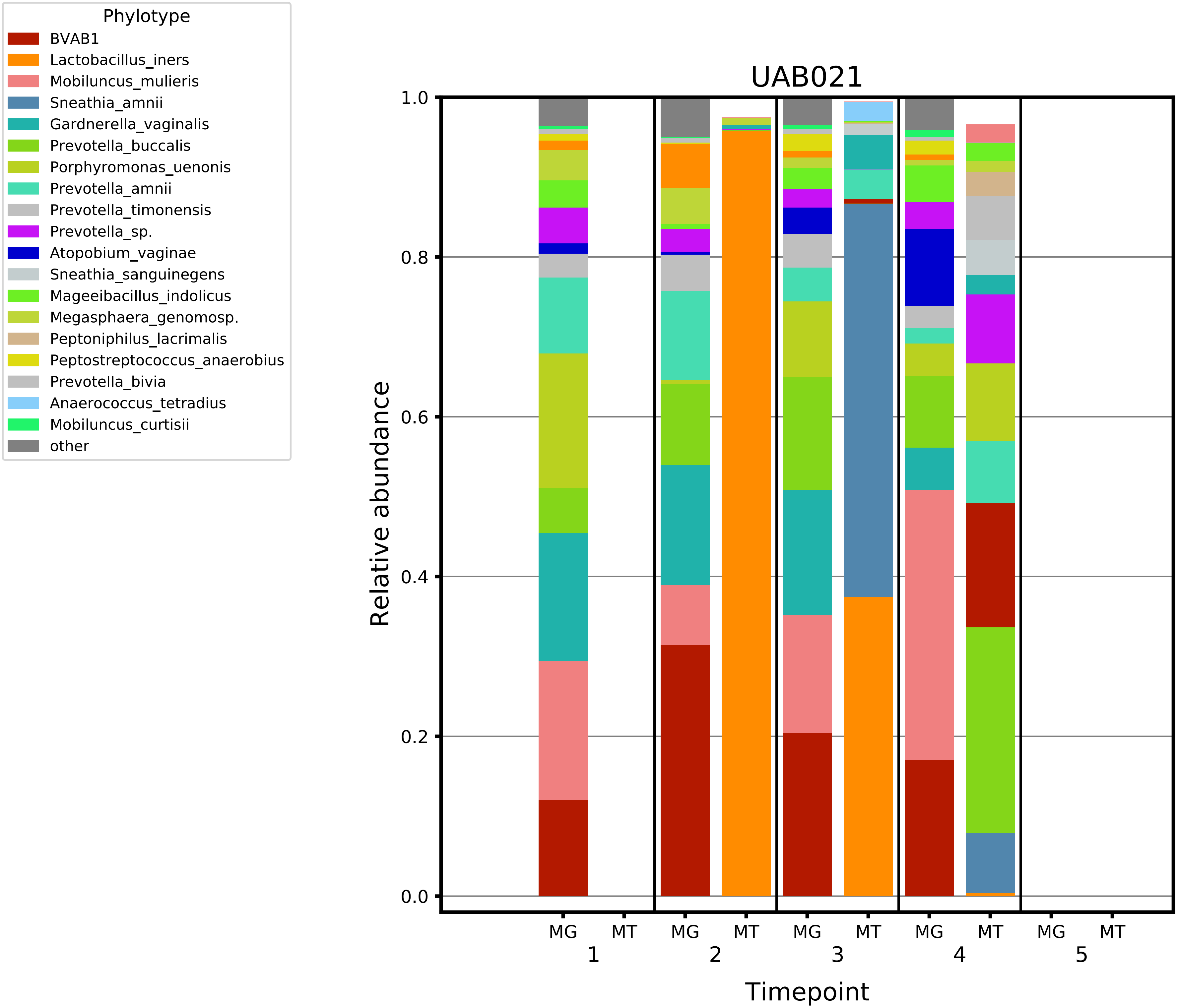

Phylotype
BVAB1
Lactobacillus_iners
Mobiluncus_mulieris
Sneathia_amnii
Gardnerella_vaginalis
Prevotella_buccalis
Porphyromonas_uenonis
Prevotella_amnii
Prevotella_timonensis
Prevotella_sp.
Atopobium_vaginae
Sneathia_sanguinegens
Mageeibacillus_indolicus
Megasphaera_genomosp.
Peptoniphilus_lacrimalis
Peptostreptococcus_anaerobius
Prevotella_bivia
Anaerococcus_tetradius
Mobiluncus_curtisii
other
UAB021
Relative abundance
1.0
0.8
0.6
0.4
0.2
0.0
MG MT MG MT MG MT MG MT MG MT
1 2 3 4 5
Timepoint

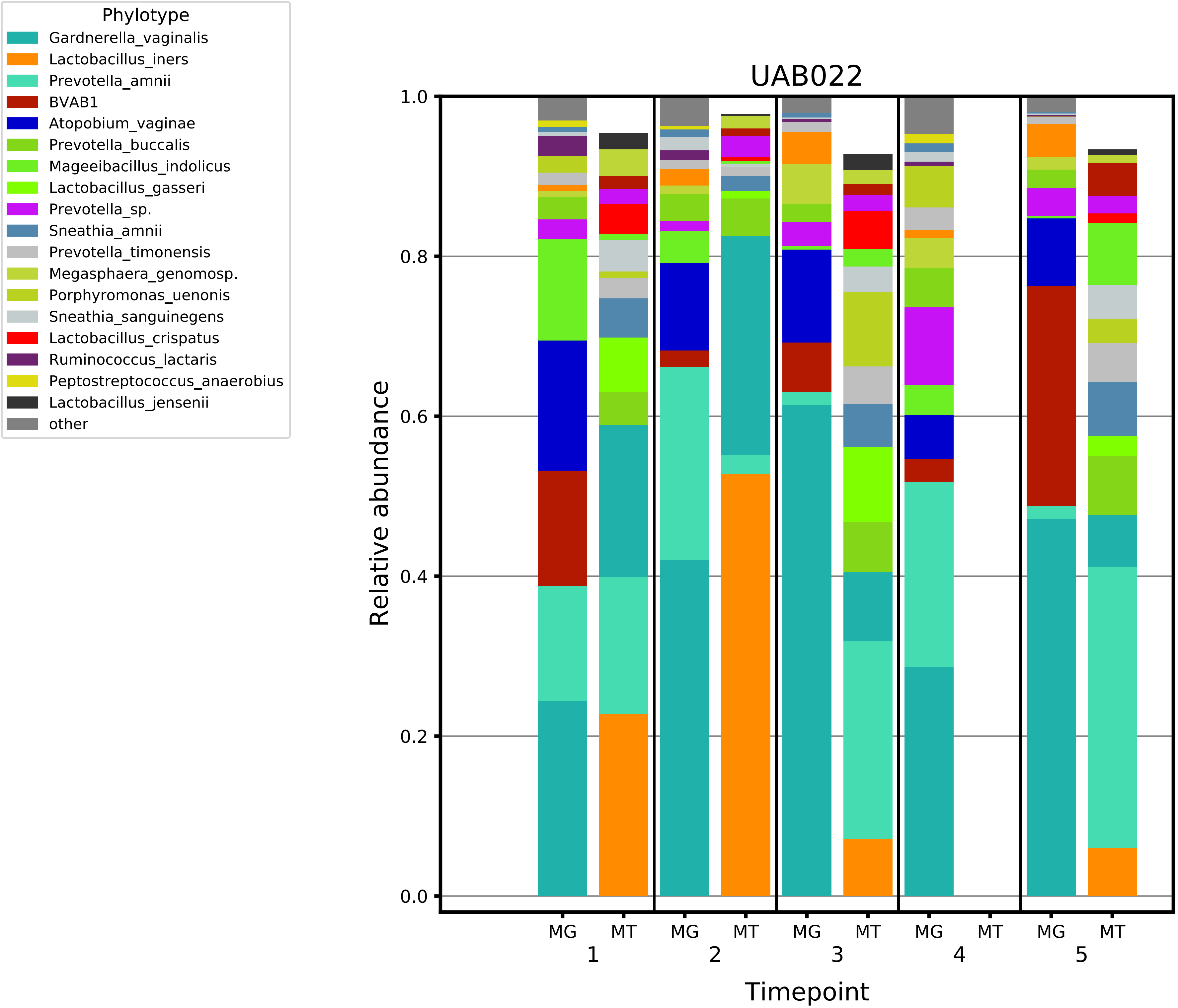

Phylotype
Gardnerella_vaginalis
Lactobacillus_iners
Prevotella_amnii
BVAB1
Atopobium_vaginae
Prevotella_buccalis
Mageeibacillus_indolicus
Lactobacillus_gasseri
Prevotella_sp.
Sneathia_amnii
Prevotella_timonensis
Megasphaera_genomosp.
Porphyromonas_uenonis
Sneathia_sanguinegens
Lactobacillus_crispatus
Ruminococcus_lactaris
Peptostreptococcus_anaerobius
Lactobacillus_jensenii
other
UAB022
Relative abundance
1.0
0.8
0.6
0.4
0.2
0.0
MG
MT
1
2
3
4
5
Timepoint

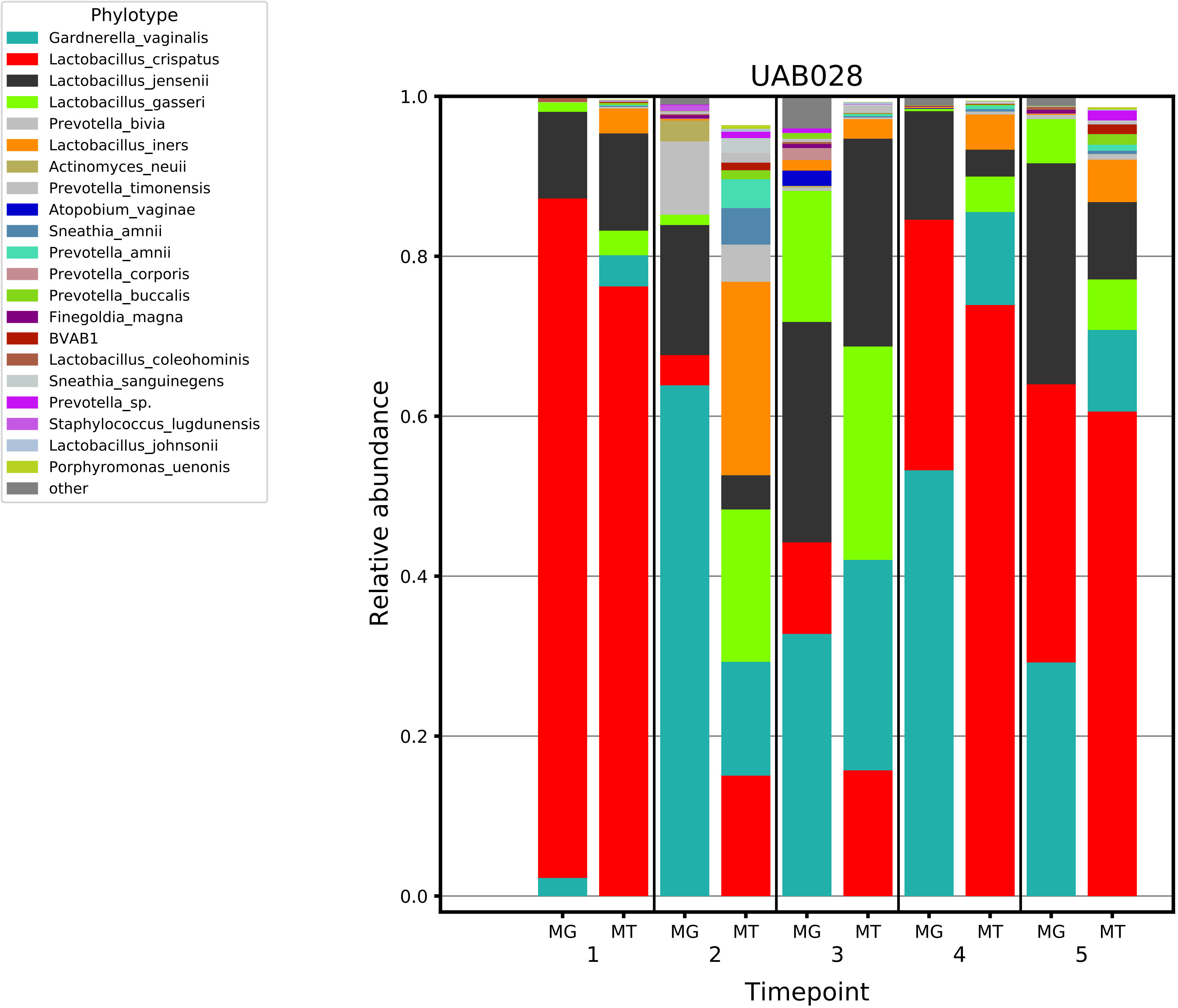
Phylotype
Gardnerella_vaginalis
Lactobacillus_crispatus
Lactobacillus_jensenii
Lactobacillus_gasseri
Prevotella_bivia
Lactobacillus_iners
Actinomyces_neuii
Prevotella_timonensis
Atopobium_vaginae
Sneathia_amnii
Prevotella_amnii
Prevotella_corporis
Prevotella_buccalis
Finegoldia_magna
BVAB1
Lactobacillus_coleohominis
Sneathia_sanguinegens
Prevotella_sp.
Staphylococcus_lugdunensis
Lactobacillus_johnsonii
Porphyromonas_uenonis
other
UAB028
Relative abundance
1.0
0.8
0.6
0.4
0.2
0.0
MG
MT
1
2
3
4
5
Timepoint

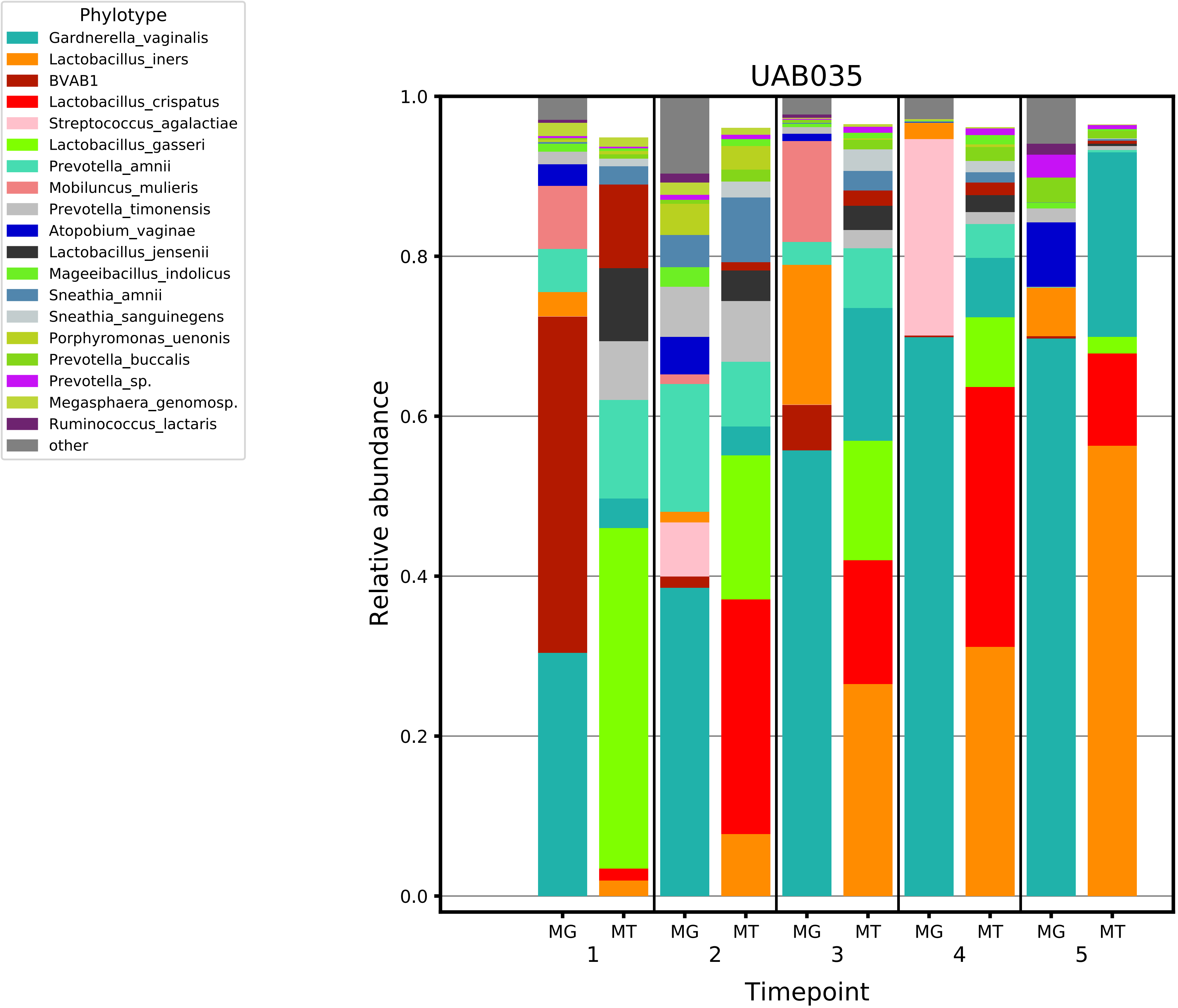

Phylotype
Gardnerella_vaginalis
Lactobacillus_iners
BVAB1
Lactobacillus_crispatus
Streptococcus_agalactiae
Lactobacillus_gasseri
Prevotella_amnii
Mobiluncus_mulieris
Prevotella_timonensis
Atopobium_vaginae
Lactobacillus_jensenii
Mageeibacillus_indolicus
Sneathia_amnii
Sneathia_sanguinegens
Porphyromonas_uenonis
Prevotella_buccalis
Prevotella_sp.
Megasphaera_genomosp.
Ruminococcus_lactaris
other
UAB035
Relative abundance
1.0
0.8
0.6
0.4
0.2
0.0
MG
MT
1
2
3
4
5
Timepoint

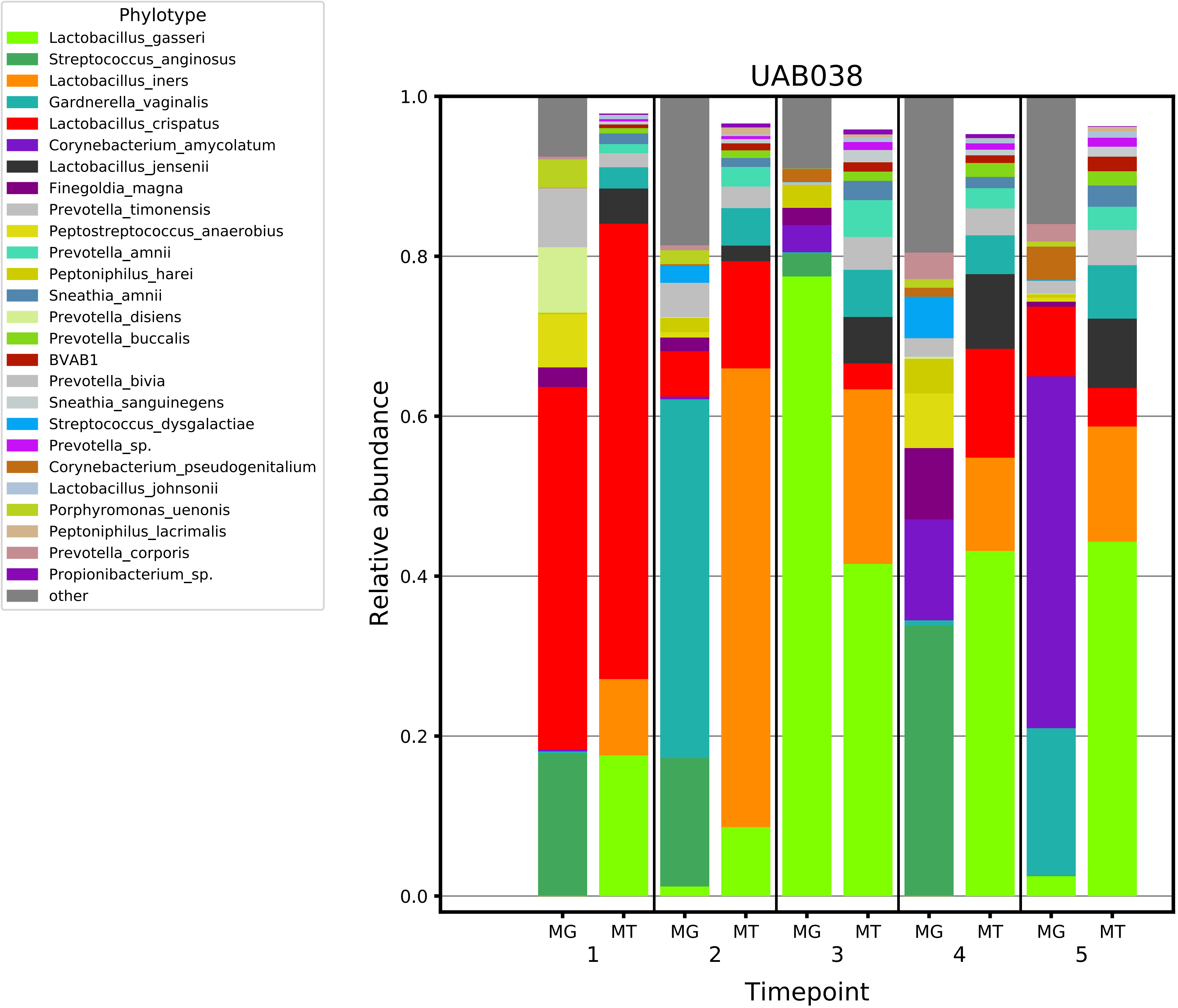

Phylotype
Lactobacillus_gasseri
Streptococcus_anginosus
Lactobacillus_iners
Gardnerella_vaginalis
Lactobacillus_crispatus
Corynebacterium_amycolatum
Lactobacillus_jensenii
Finegoldia_magna
Prevotella_timonensis
Peptostreptococcus_anaerobius
Prevotella_amnii
Peptoniphilus_harei
Sneathia_amnii
Prevotella_disiens
Prevotella_buccalis
BVAB1
Prevotella_bivia
Sneathia_sanguinegens
Streptococcus_dysgalactiae
Prevotella_sp.
Corynebacterium_pseudogenitalium
Lactobacillus_johnsonii
Porphyromonas_uenonis
Peptoniphilus_lacrimalis
Prevotella_corporis
Propionibacterium_sp.
other
UAB038
Relative abundance
1.0
0.8
0.6
0.4
0.2
0.0
MG
MT
1
2
3
4
5
Timepoint

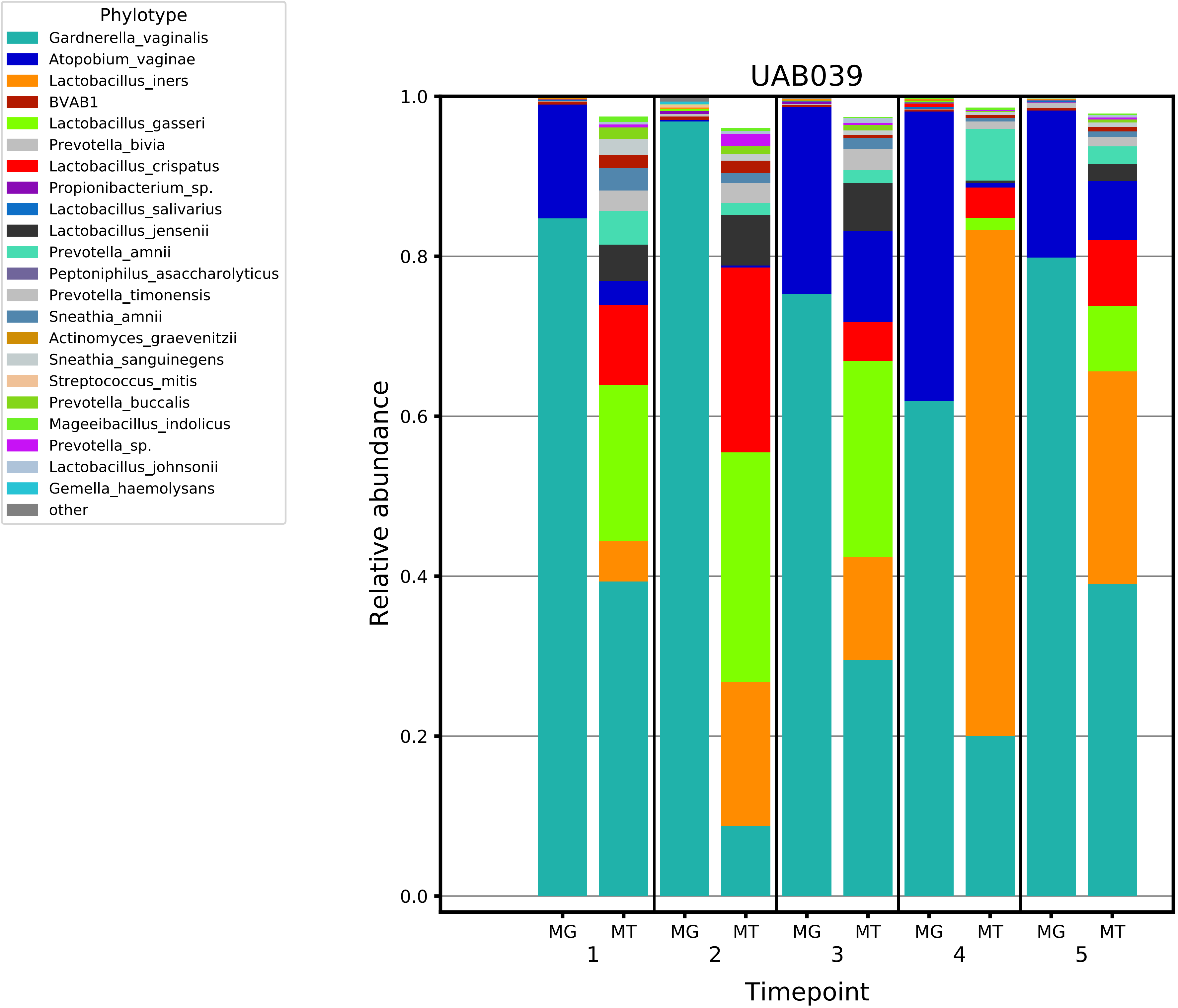
Phylotype
Gardnerella_vaginalis
Atopobium_vaginae
Lactobacillus_iners
BVAB1
Lactobacillus_gasseri
Prevotella_bivia
Lactobacillus_crispatus
Propionibacterium_sp.
Lactobacillus_salivarius
Lactobacillus_jensenii
Prevotella_amnii
Peptoniphilus_asaccharolyticus
Prevotella_timonensis
Sneathia_amnii
Actinomyces_graevenitzii
Sneathia_sanguinegens
Streptococcus_mitis
Prevotella_buccalis
Mageeibacillus_indolicus
Prevotella_sp.
Lactobacillus_johnsonii
Gemella_haemolysans
other
UAB039
Relative abundance
1.0
0.8
0.6
0.4
0.2
0.0
MG
MT
1
2
3
4
5
Timepoint

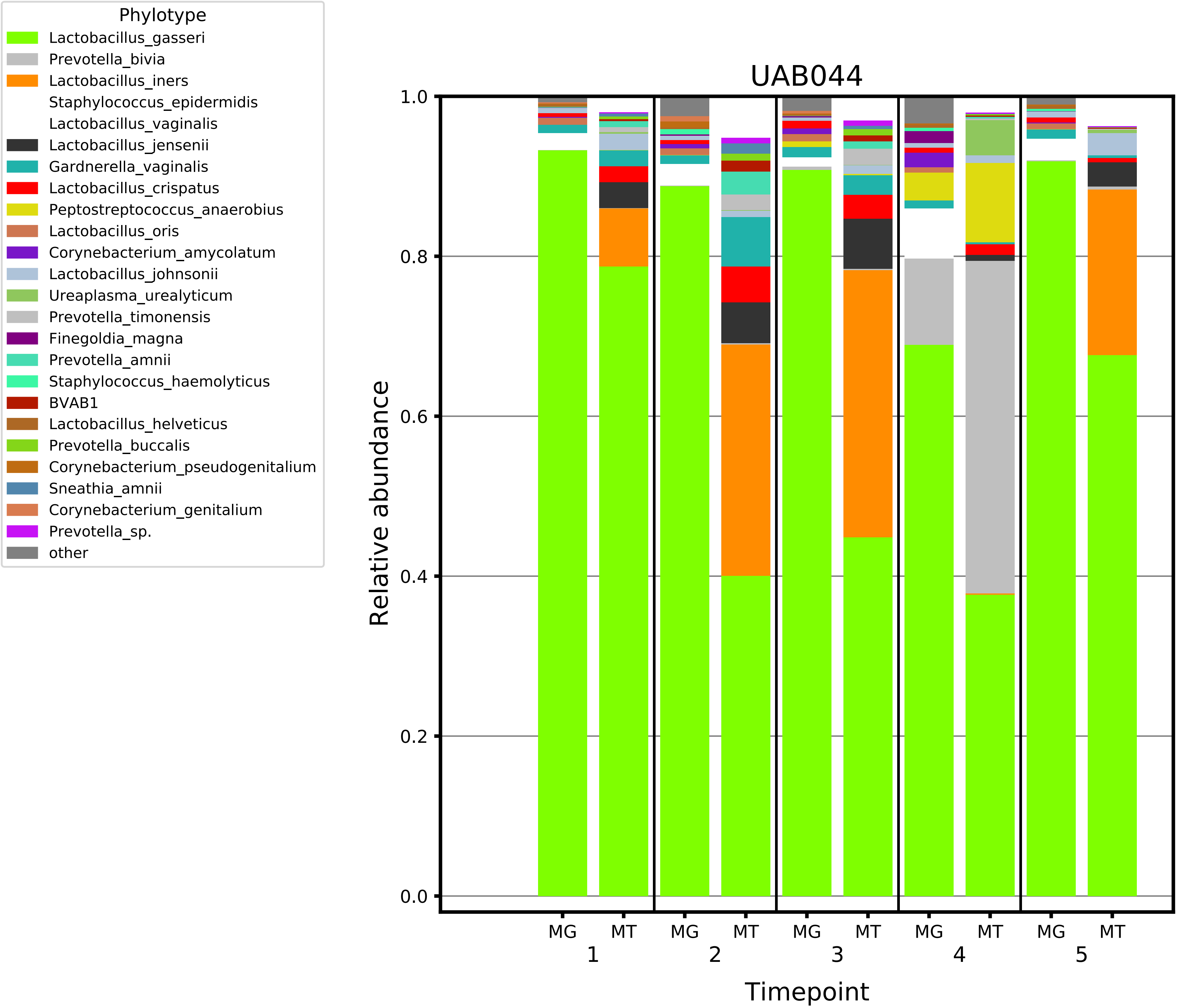
Phylotype
Lactobacillus_gasseri
Prevotella_bivia
Lactobacillus_iners
Staphylococcus_epidermidis
Lactobacillus_vaginalis
Lactobacillus_jensenii
Gardnerella_vaginalis
Lactobacillus_crispatus
Peptostreptococcus_anaerobius
Lactobacillus_oris
Corynebacterium_amycolatum
Lactobacillus_johnsonii
Ureaplasma_urealyticum
Prevotella_timonensis
Finegoldia_magna
Prevotella_amnii
Staphylococcus_haemolyticus
BVAB1
Lactobacillus_helveticus
Prevotella_buccalis
Corynebacterium_pseudogenitalium
Sneathia_amnii
Corynebacterium_genitalium
Prevotella_sp.
other
UAB044
Relative abundance
1.0
0.8
0.6
0.4
0.2
0.0
MG
MT
1
2
3
4
5
Timepoint

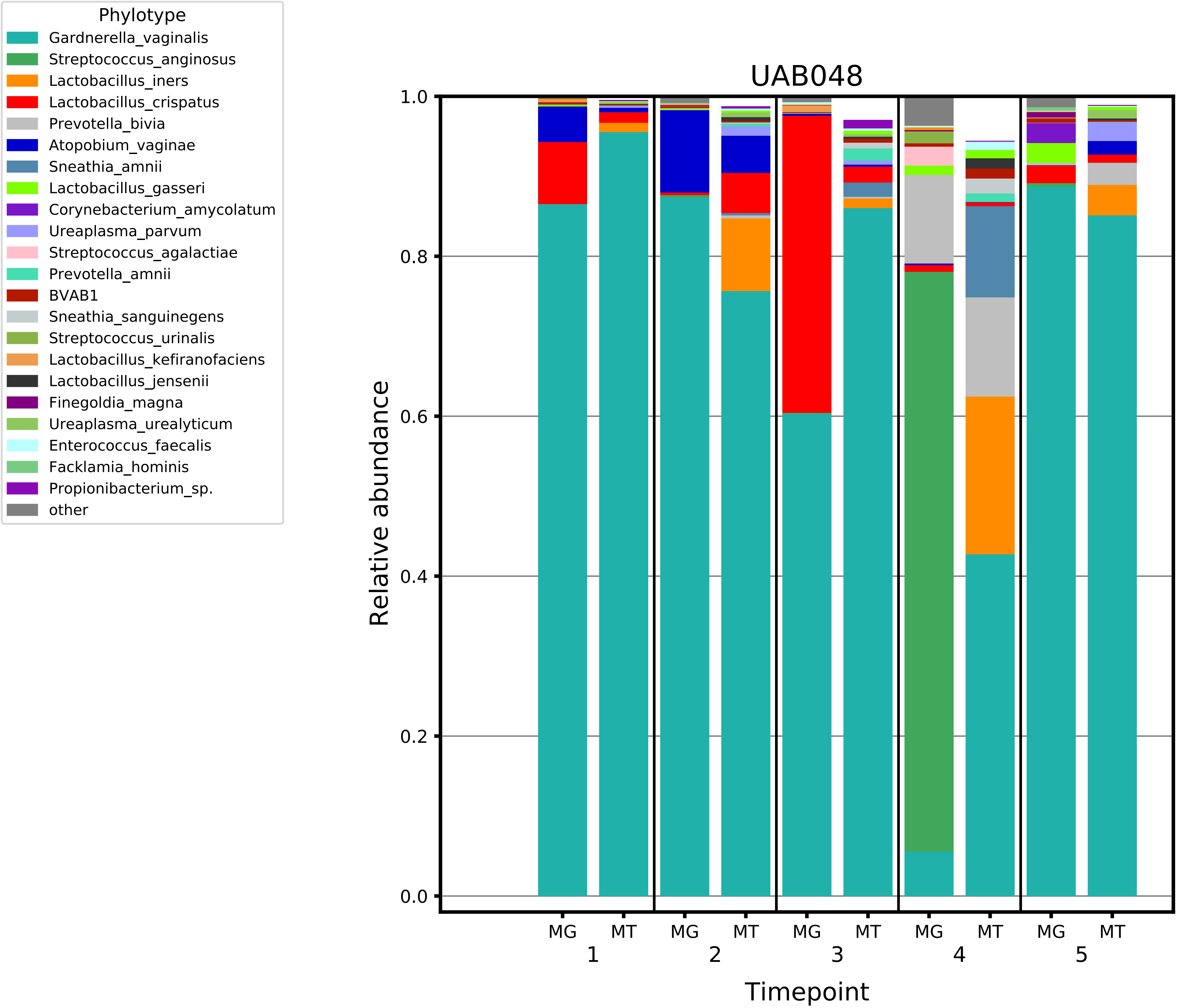

Phylotype
Gardnerella_vaginalis
Streptococcus_anginosus
Lactobacillus_iners
Lactobacillus_crispatus
Prevotella_bivia
Atopobium_vaginae
Sneathia_amnii
Lactobacillus_gasseri
Corynebacterium_amycolatum
Ureaplasma_parvum
Streptococcus_agalactiae
Prevotella_amnii
BVAB1
Sneathia_sanguinegens
Streptococcus_urinalis
Lactobacillus_kefiranofaciens
Lactobacillus_jensenii
Finegoldia_magna
Ureaplasma_urealyticum
Enterococcus_faecalis
Facklamia_hominis
Propionibacterium_sp.
other
UAB048
Relative abundance
1.0
0.8
0.6
0.4
0.2
0.0
MG MT MG MT MG MT MG MT MG MT
1 2 3 4 5
Timepoint

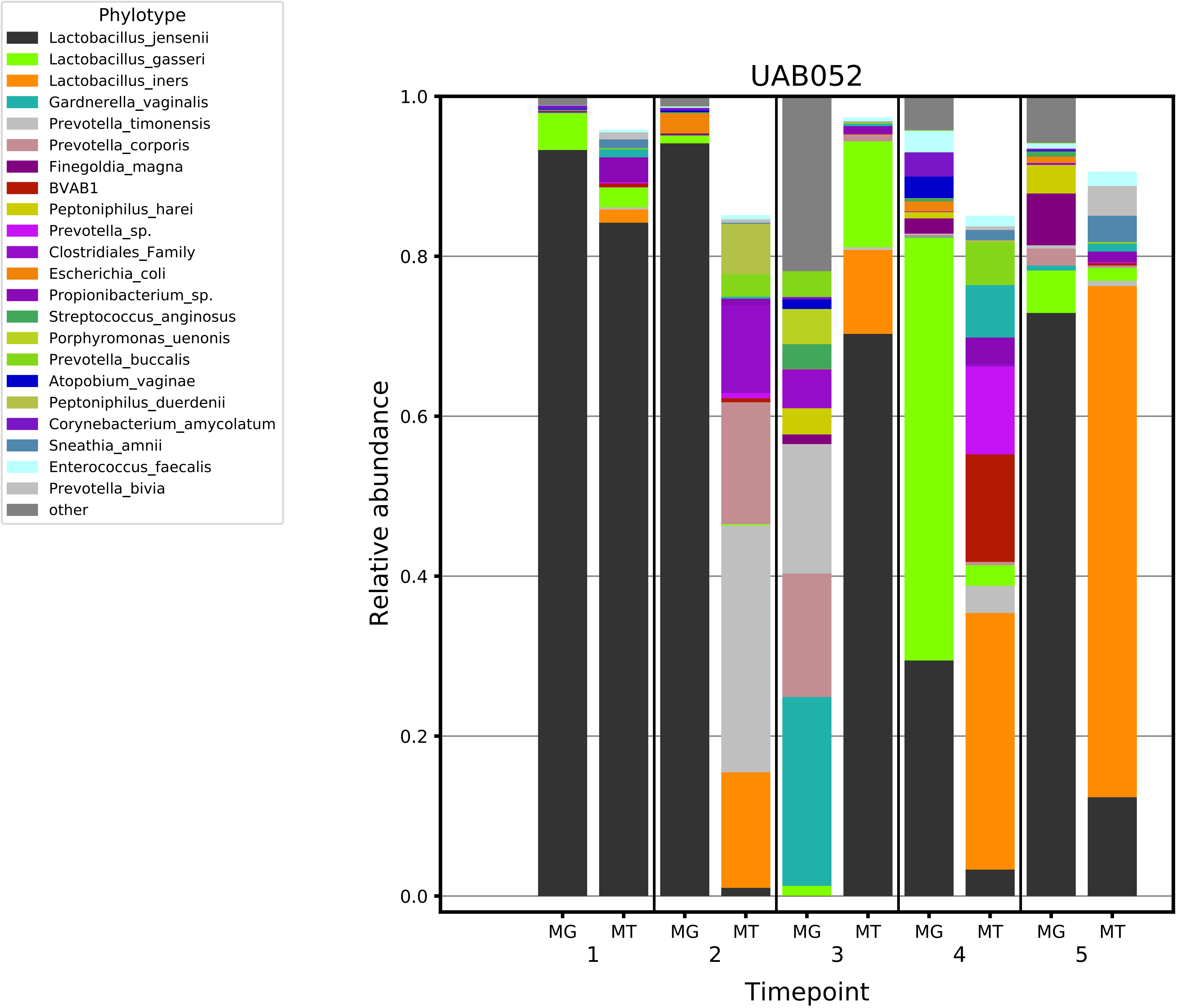
Phylotype
Lactobacillus_jensenii
Lactobacillus_gasseri
Lactobacillus_iners
Gardnerella_vaginalis
Prevotella_timonensis
Prevotella_corporis
Finegoldia_magna
BVAB1
Peptoniphilus_harei
Prevotella_sp.
Clostridiales_Family
Escherichia_coli
Propionibacterium_sp.
Streptococcus_anginosus
Porphyromonas_uenonis
Prevotella_buccalis
Atopobium_vaginae
Peptoniphilus_duerdenii
Corynebacterium_amycolatum
Sneathia_amnii
Enterococcus_faecalis
Prevotella_bivia
other
UAB052
Relative abundance
1.0
0.8
0.6
0.4
0.2
0.0
MG
MT
1
2
3
4
5
Timepoint

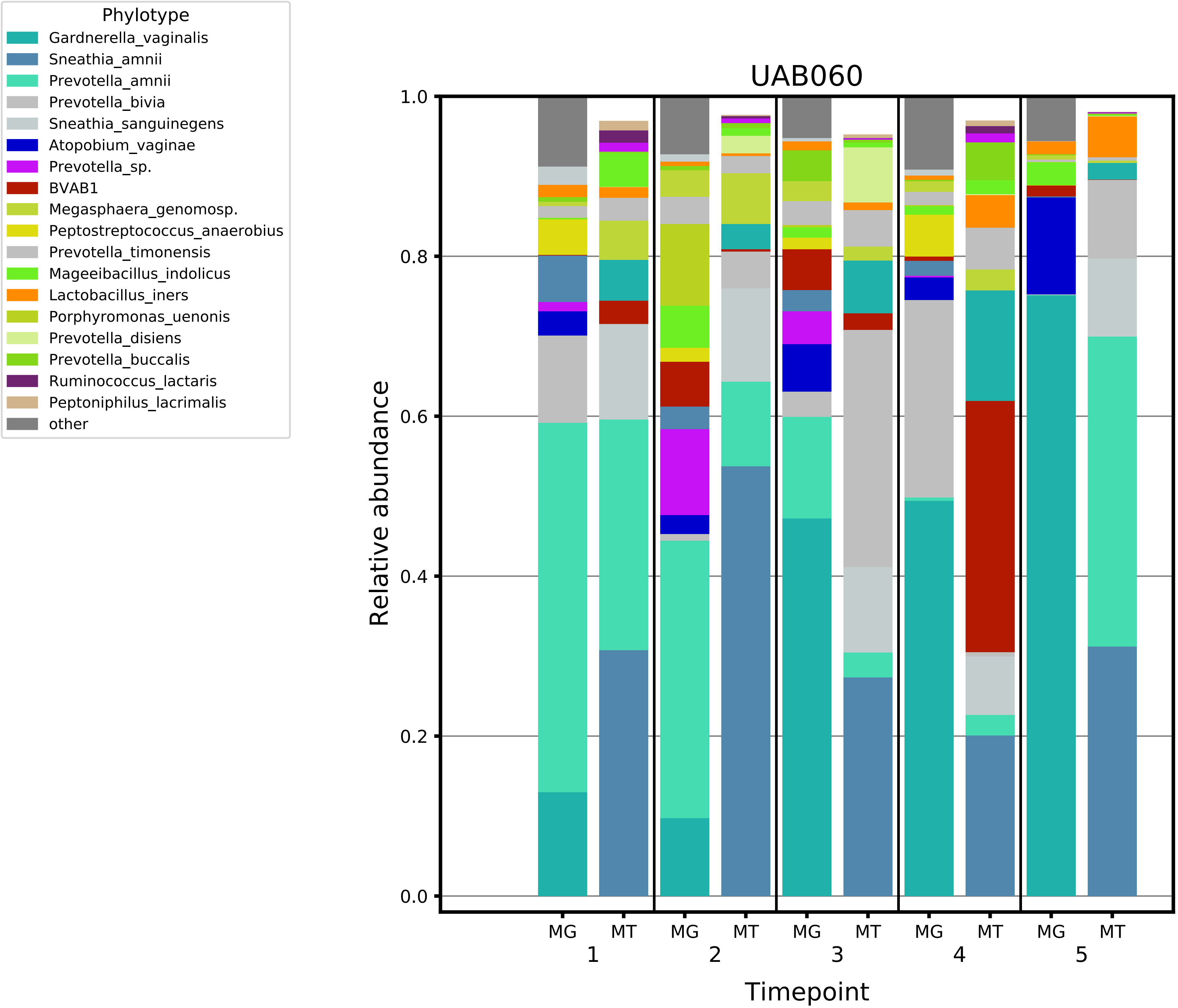
Phylotype
Gardnerella_vaginalis
Sneathia_amnii
Prevotella_amnii
Prevotella_bivia
Sneathia_sanguinegens
Atopobium_vaginae
Prevotella_sp.
BVAB1
Megasphaera_genomosp.
Peptostreptococcus_anaerobius
Prevotella_timonensis
Mageeibacillus_indolicus
Lactobacillus_iners
Porphyromonas_uenonis
Prevotella_disiens
Prevotella_buccalis
Ruminococcus_lactaris
Peptoniphilus_lacrimalis
other
UAB060
Relative abundance
1.0
0.8
0.6
0.4
0.2
0.0
MG
MT
1
2
3
4
5
Timepoint

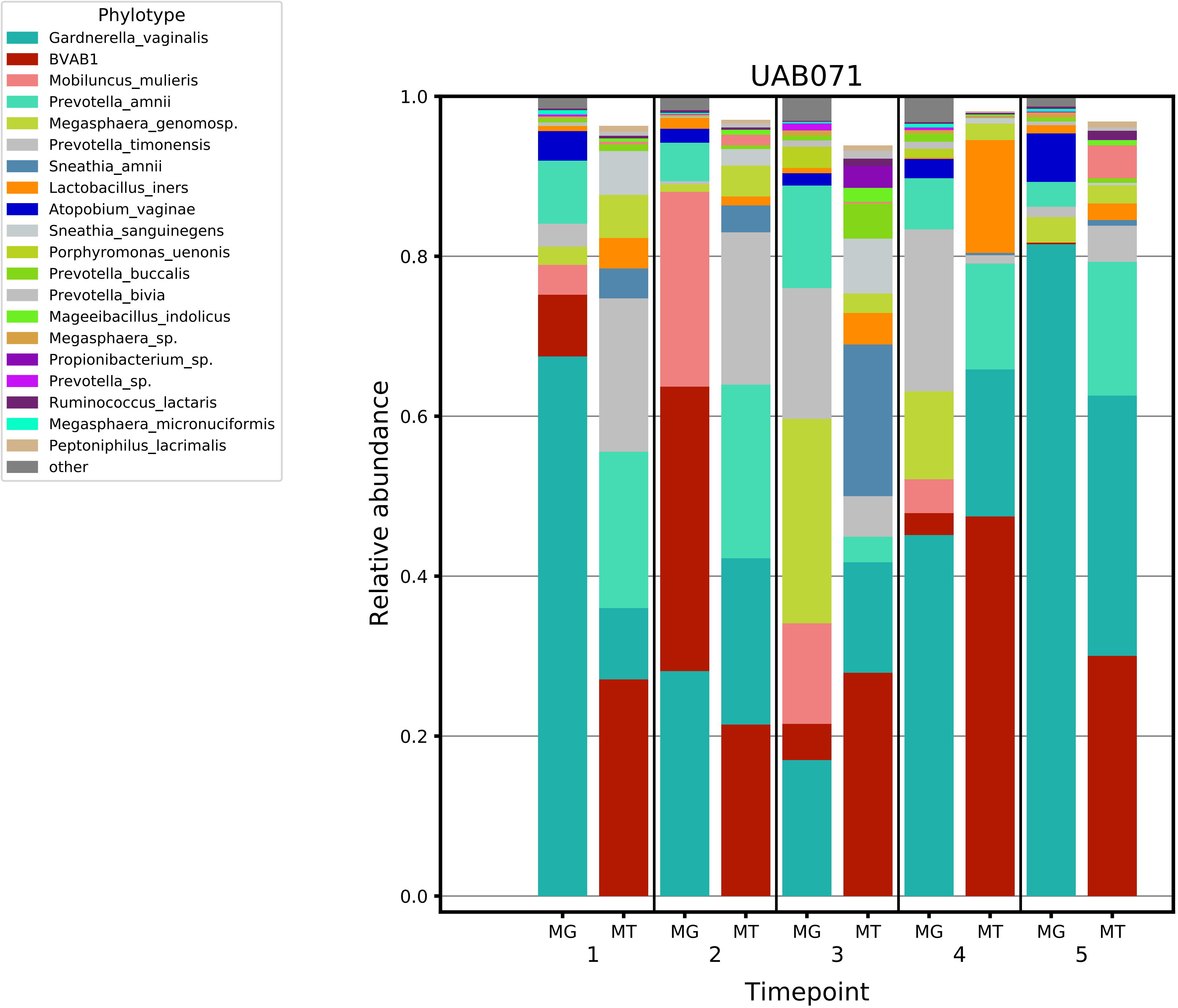
Phylotype
Gardnerella_vaginalis
BVAB1
Mobiluncus_mulieris
Prevotella_amnii
Megasphaera_genomosp.
Prevotella_timonensis
Sneathia_amnii
Lactobacillus_iners
Atopobium_vaginae
Sneathia_sanguinegens
Porphyromonas_uenonis
Prevotella_buccalis
Prevotella_bivia
Mageeibacillus_indolicus
Megasphaera_sp.
Propionibacterium_sp.
Prevotella_sp.
Ruminococcus_lactaris
Megasphaera_micronuciformis
Peptoniphilus_lacrimalis
other
UAB071
Relative abundance
1.0
0.8
0.6
0.4
0.2
0.0
MG
MT
1
2
3
4
5
Timepoint

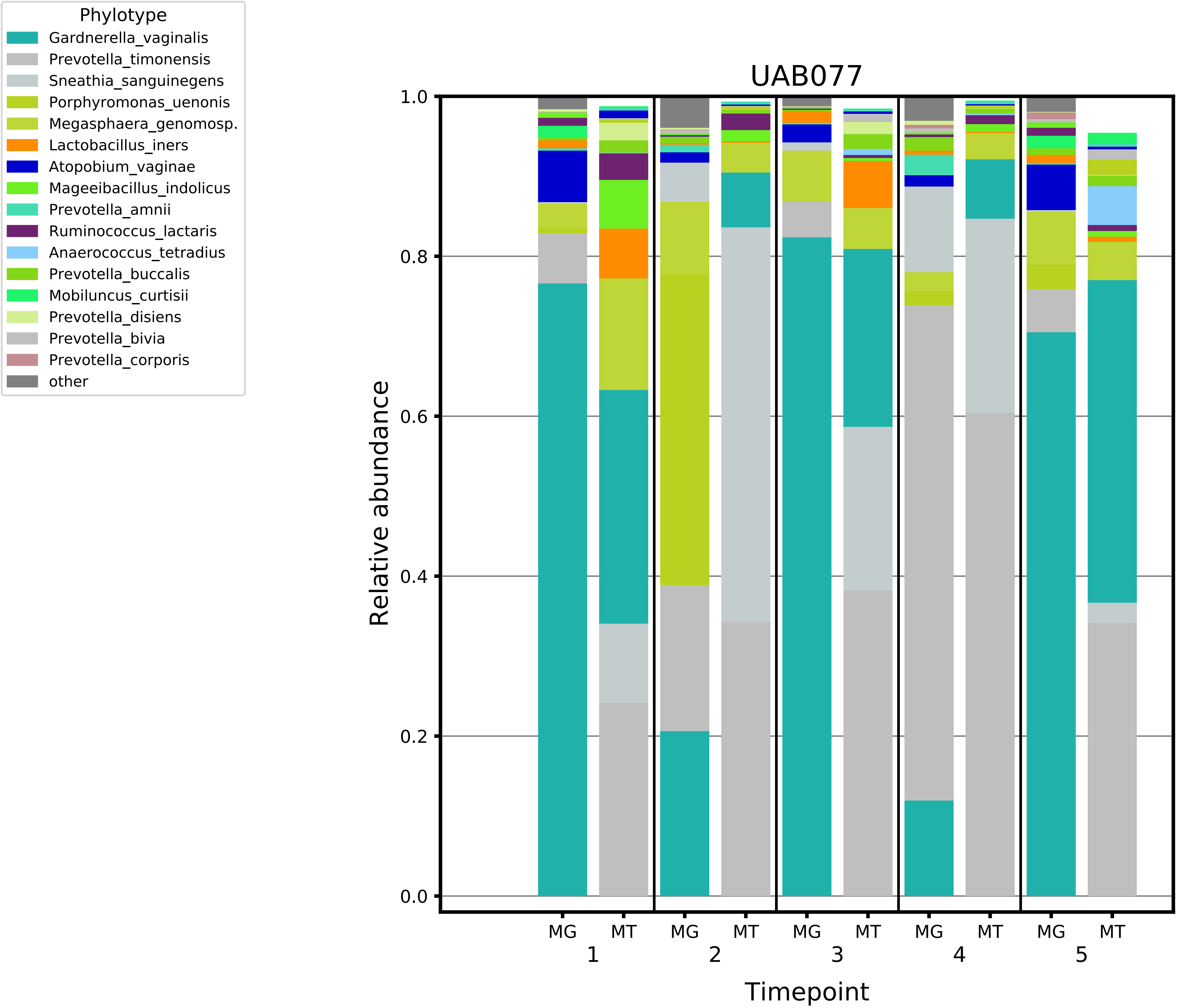
Phylotype
Gardnerella_vaginalis
Prevotella_timonensis
Sneathia_sanguinegens
Porphyromonas_uenonis
Megasphaera_genomosp.
Lactobacillus_iners
Atopobium_vaginae
Mageeibacillus_indolicus
Prevotella_amnii
Ruminococcus_lactaris
Anaerococcus_tetradius
Prevotella_buccalis
Mobiluncus_curtisii
Prevotella_disiens
Prevotella_bivia
Prevotella_corporis
other
UAB077
Relative abundance
0.0
0.2
0.4
0.6
0.8
1.0
MG
MT
1
2
3
4
5
Timepoint

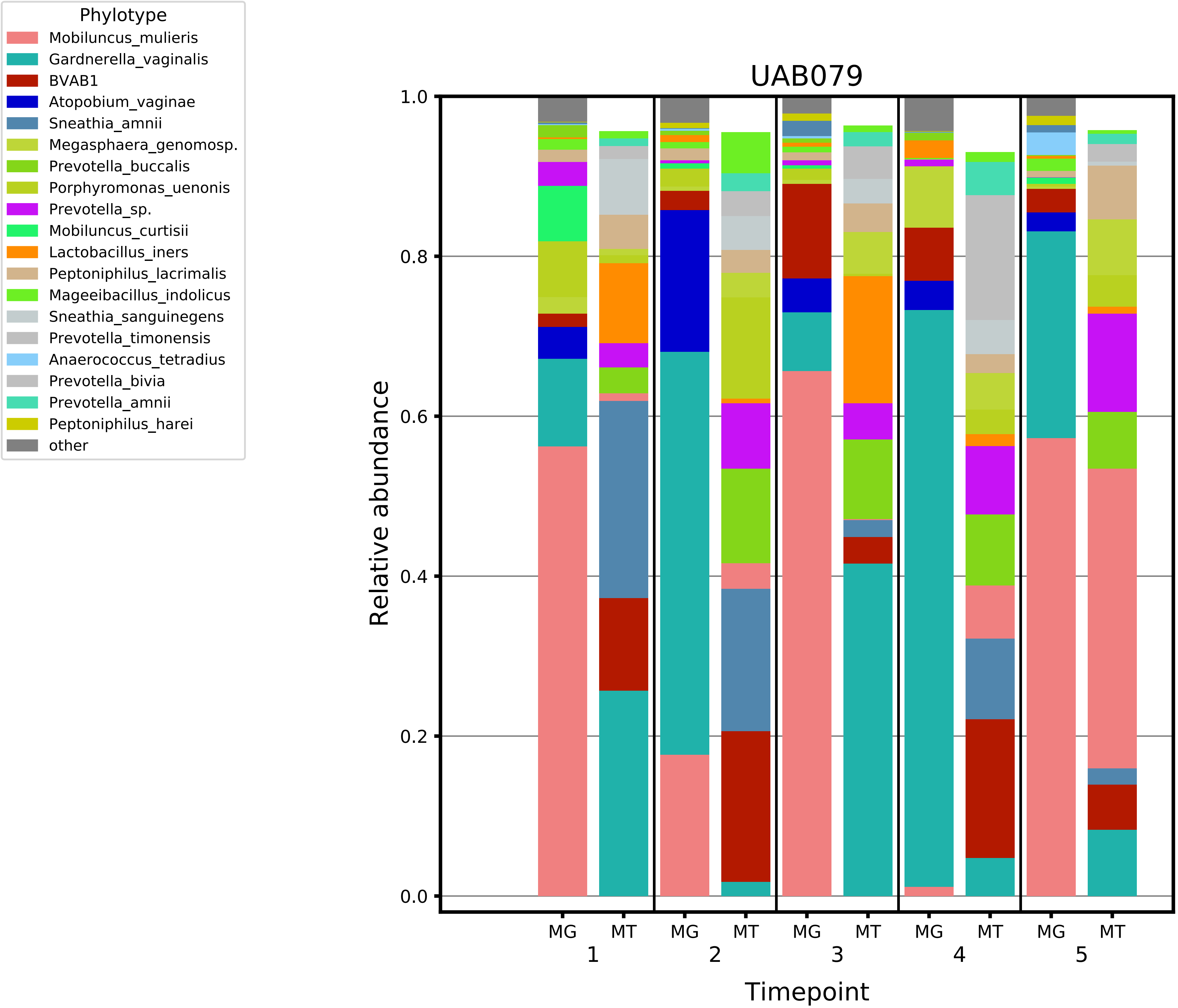

Phylotype
Mobiluncus_mulieris
Gardnerella_vaginalis
BVAB1
Atopobium_vaginae
Sneathia_amnii
Megasphaera_genomosp.
Prevotella_buccalis
Porphyromonas_uenonis
Prevotella_sp.
Mobiluncus_curtisii
Lactobacillus_iners
Peptoniphilus_lacrimalis
Mageeibacillus_indolicus
Sneathia_sanguinegens
Prevotella_timonensis
Anaerococcus_tetradius
Prevotella_bivia
Prevotella_amnii
Peptoniphilus_harei
other
UAB079
Relative abundance
0.0
0.2
0.4
0.6
0.8
1.0
MG
MT
1
2
3
4
5
Timepoint

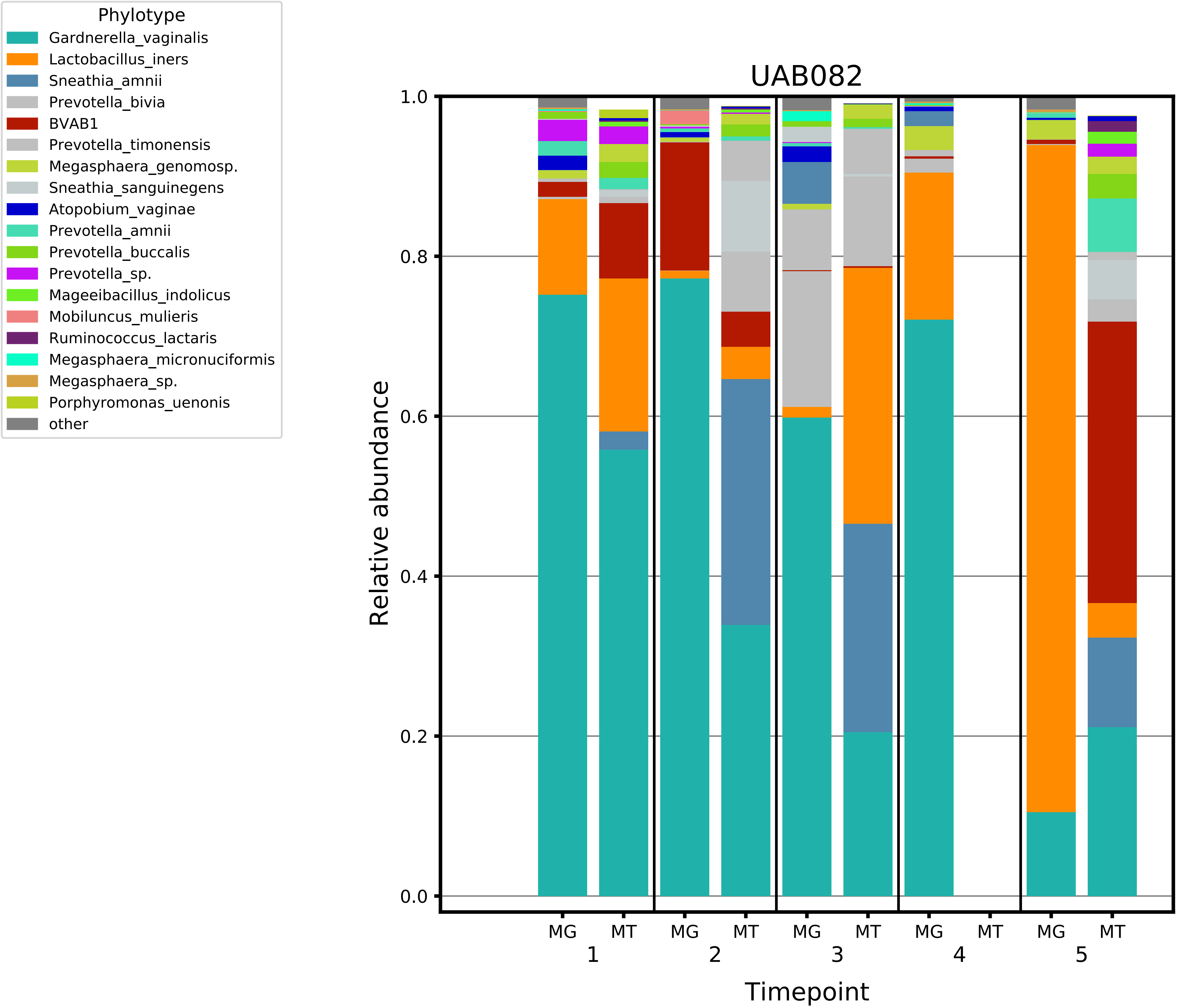
Phylotype
Gardnerella_vaginalis
Lactobacillus_iners
Sneathia_amnii
Prevotella_bivia
BVAB1
Prevotella_timonensis
Megasphaera_genomosp.
Sneathia_sanguinegens
Atopobium_vaginae
Prevotella_amnii
Prevotella_buccalis
Prevotella_sp.
Mageeibacillus_indolicus
Mobiluncus_mulieris
Ruminococcus_lactaris
Megasphaera_micronuciformis
Megasphaera_sp.
Porphyromonas_uenonis
other
UAB082
Relative abundance
1.0
0.8
0.6
0.4
0.2
0.0
MG
MT
1
2
3
4
5
Timepoint

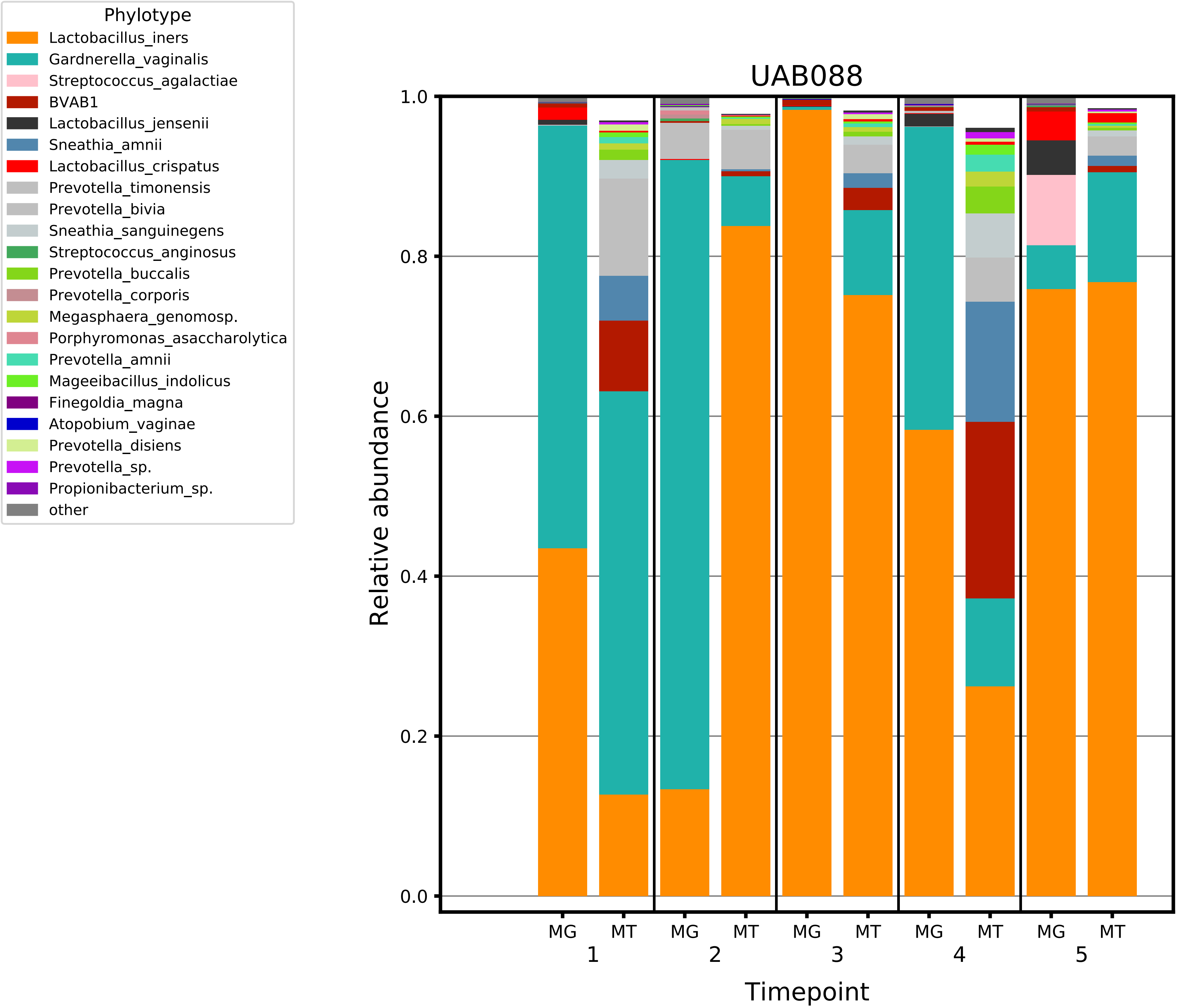

Phylotype
Lactobacillus_iners
Gardnerella_vaginalis
Streptococcus_agalactiae
BVAB1
Lactobacillus_jensenii
Sneathia_amnii
Lactobacillus_crispatus
Prevotella_timonensis
Prevotella_bivia
Sneathia_sanguinegens
Streptococcus_anginosus
Prevotella_buccalis
Prevotella_corporis
Megasphaera_genomosp.
Porphyromonas_asaccharolytica
Prevotella_amnii
Mageeibacillus_indolicus
Finegoldia_magna
Atopobium_vaginae
Prevotella_disiens
Prevotella_sp.
Propionibacterium_sp.
other
UAB088
Relative abundance
1.0
0.8
0.6
0.4
0.2
0.0
MG
MT
1
2
3
4
5
Timepoint

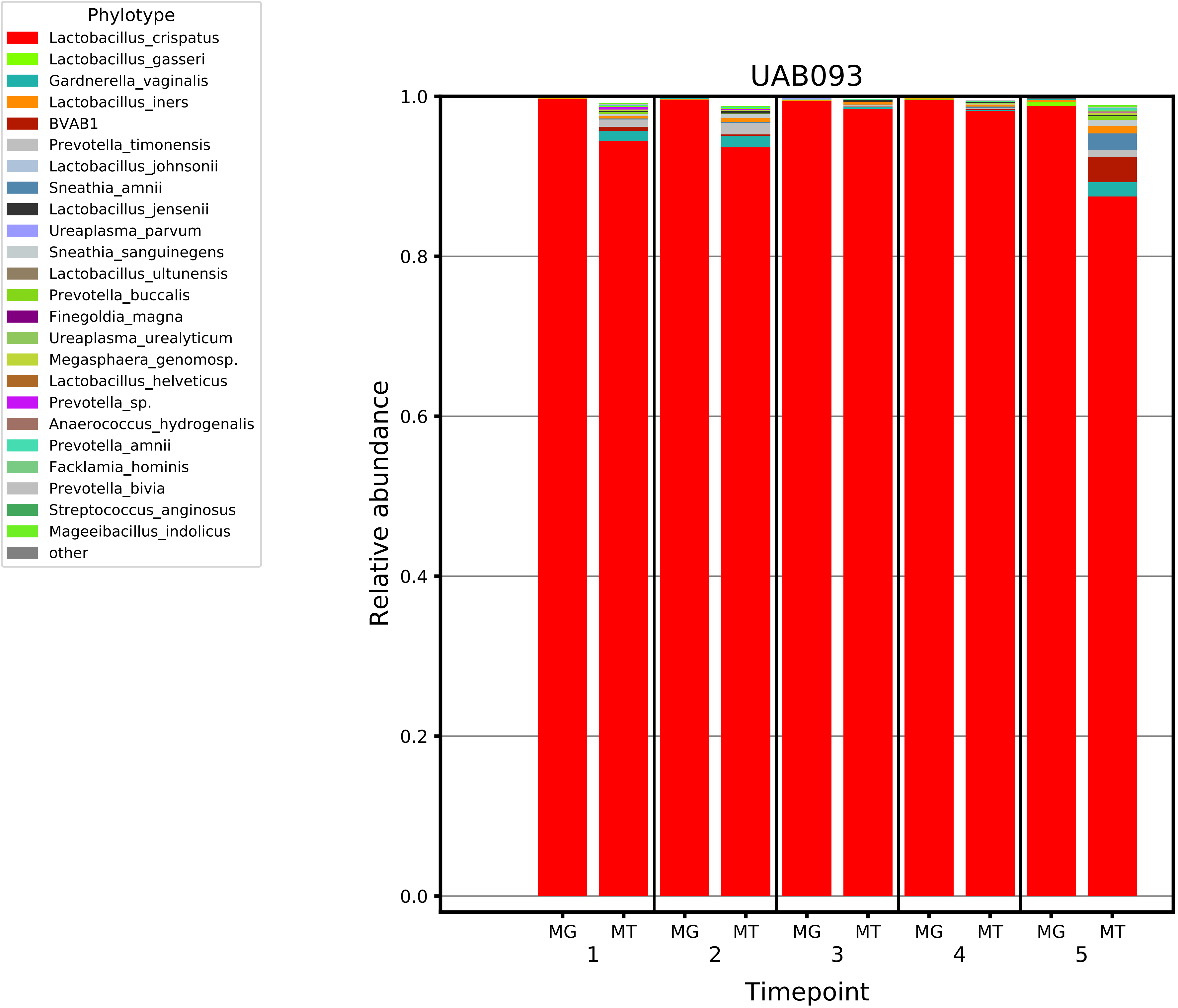
Phylotype
Lactobacillus_crispatus
Lactobacillus_gasseri
Gardnerella_vaginalis
Lactobacillus_iners
BVAB1
Prevotella_timonensis
Lactobacillus_johnsonii
Sneathia_amnii
Lactobacillus_jensenii
Ureaplasma_parvum
Sneathia_sanguinegens
Lactobacillus_ultunensis
Prevotella_buccalis
Finegoldia_magna
Ureaplasma_urealyticum
Megasphaera_genomosp.
Lactobacillus_helveticus
Prevotella_sp.
Anaerococcus_hydrogenalis
Prevotella_amnii
Facklamia_hominis
Prevotella_bivia
Streptococcus_anginosus
Mageeibacillus_indolicus
other
UAB093
Relative abundance
1.0
0.8
0.6
0.4
0.2
0.0
MG
MT
1
2
3
4
5
Timepoint

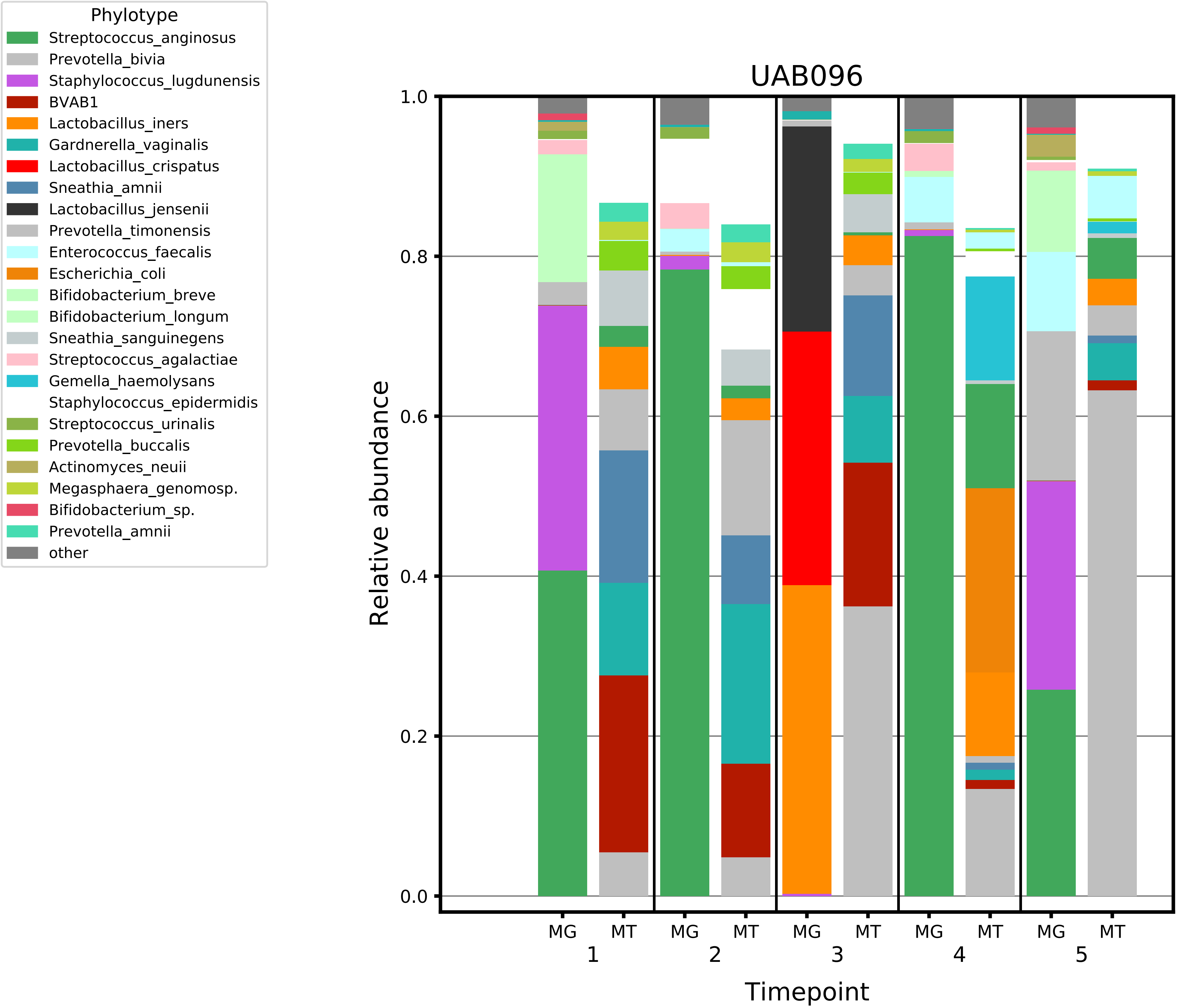

Phylotype
Streptococcus_anginosus
Prevotella_bivia
Staphylococcus_lugdunensis
BVAB1
Lactobacillus_iners
Gardnerella_vaginalis
Lactobacillus_crispatus
Sneathia_amnii
Lactobacillus_jensenii
Prevotella_timonensis
Enterococcus_faecalis
Escherichia_coli
Bifidobacterium_breve
Bifidobacterium_longum
Sneathia_sanguinegens
Streptococcus_agalactiae
Gemella_haemolysans
Staphylococcus_epidermidis
Streptococcus_urinalis
Prevotella_buccalis
Actinomyces_neuii
Megasphaera_genomosp.
Bifidobacterium_sp.
Prevotella_amnii
other
UAB096
Relative abundance
1.0
0.8
0.6
0.4
0.2
0.0
MG
MT
1
2
3
4
5
Timepoint

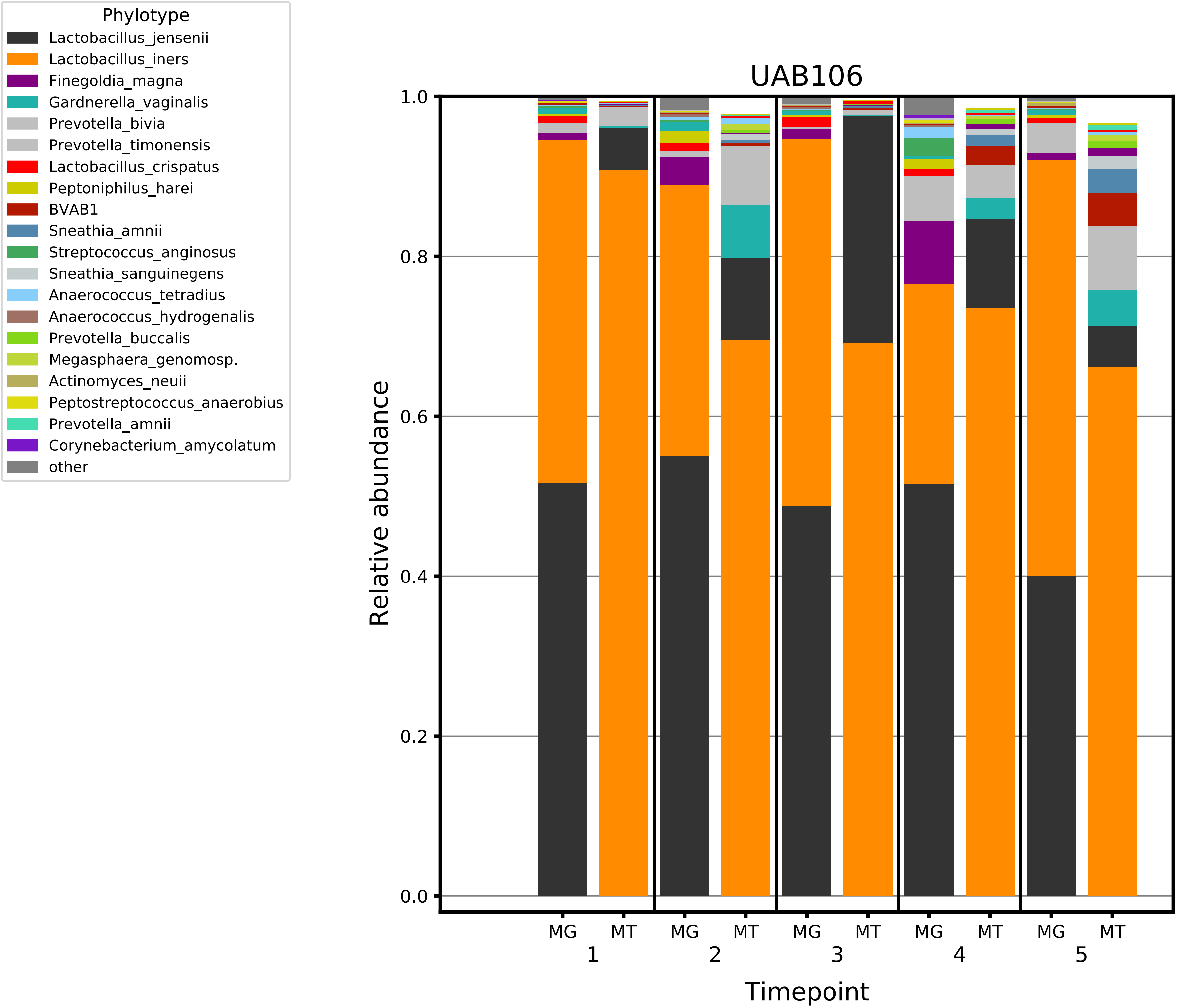

Phylotype
Lactobacillus_jensenii
Lactobacillus_iners
Finegoldia_magna
Gardnerella_vaginalis
Prevotella_bivia
Prevotella_timonensis
Lactobacillus_crispatus
Peptoniphilus_harei
BVAB1
Sneathia_amnii
Streptococcus_anginosus
Sneathia_sanguinegens
Anaerococcus_tetradius
Anaerococcus_hydrogenalis
Prevotella_buccalis
Megasphaera_genomosp.
Actinomyces_neuii
Peptostreptococcus_anaerobius
Prevotella_amnii
Corynebacterium_amycolatum
other
UAB106
Relative abundance
0.0
0.2
0.4
0.6
0.8
1.0
MG
MT
1
2
3
4
5
Timepoint

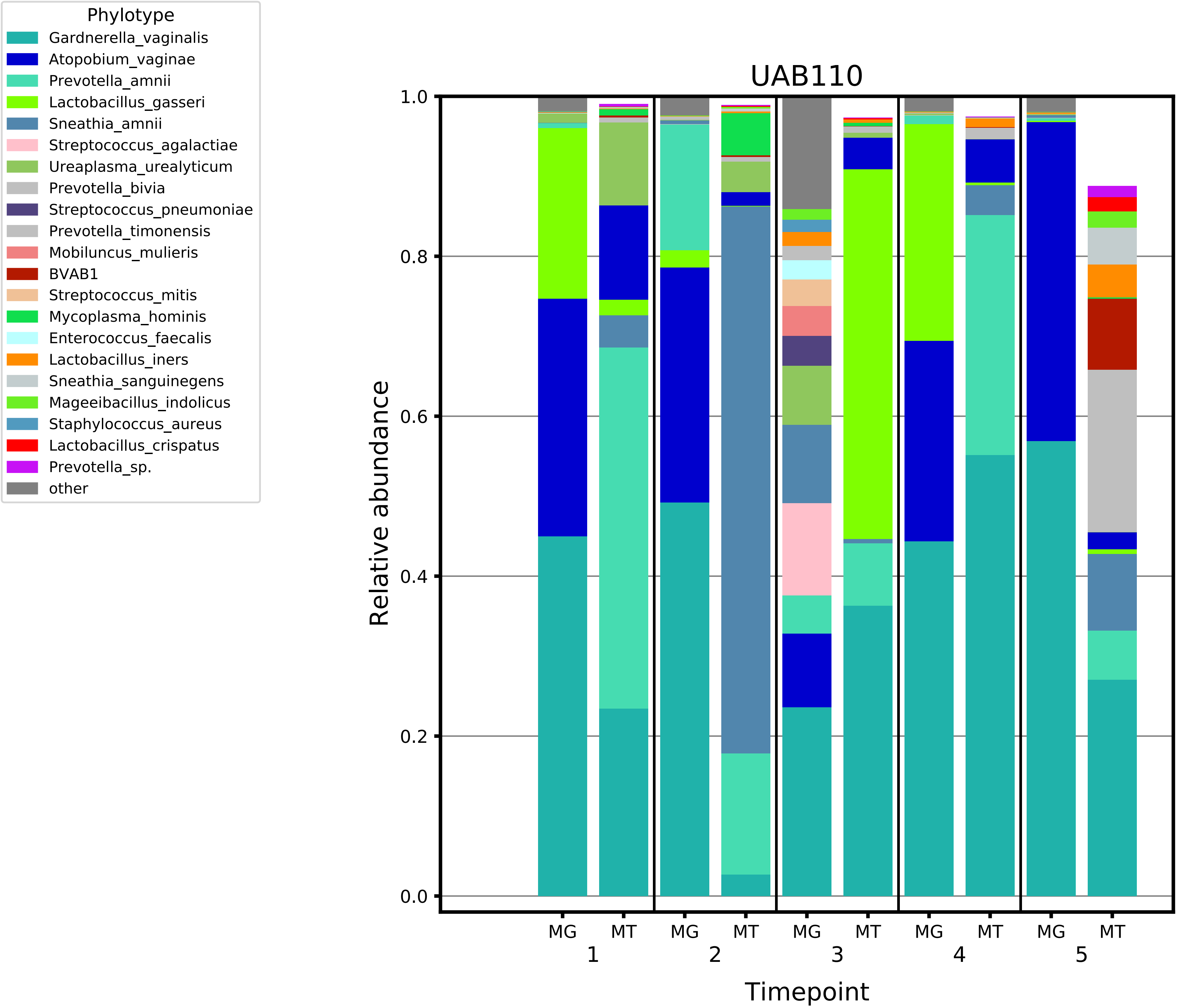
Phylotype
Gardnerella_vaginalis
Atopobium_vaginae
Prevotella_amnii
Lactobacillus_gasseri
Sneathia_amnii
Streptococcus_agalactiae
Ureaplasma_urealyticum
Prevotella_bivia
Streptococcus_pneumoniae
Prevotella_timonensis
Mobiluncus_mulieris
BVAB1
Streptococcus_mitis
Mycoplasma_hominis
Enterococcus_faecalis
Lactobacillus_iners
Sneathia_sanguinegens
Mageeibacillus_indolicus
Staphylococcus_aureus
Lactobacillus_crispatus
Prevotella_sp.
other
UAB110
Relative abundance
1.0
0.8
0.6
0.4
0.2
0.0
MG
MT
1
2
3
4
5
Timepoint

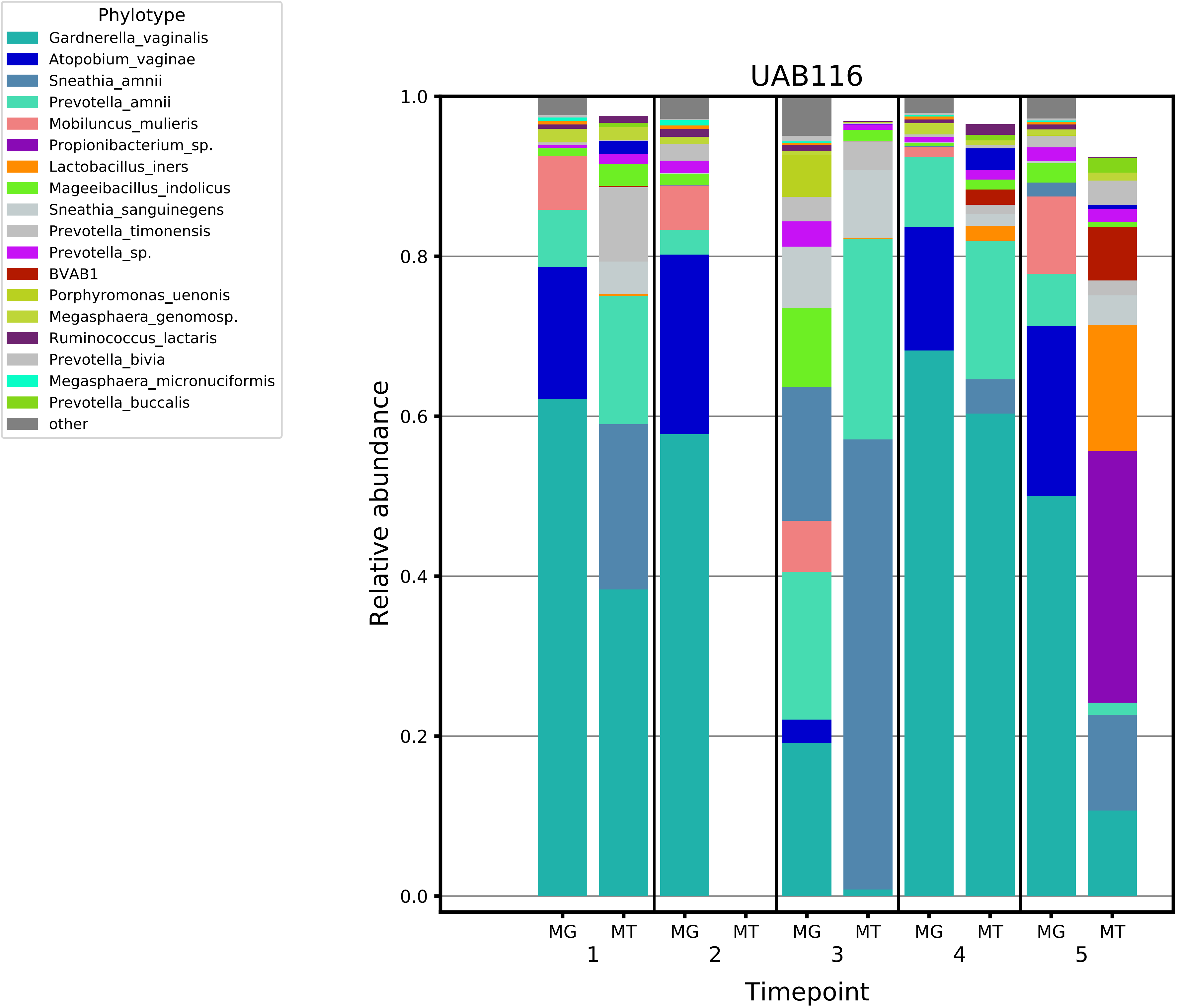

Phylotype
Gardnerella_vaginalis
Atopobium_vaginae
Sneathia_amnii
Prevotella_amnii
Mobiluncus_mulieris
Propionibacterium_sp.
Lactobacillus_iners
Mageeibacillus_indolicus
Sneathia_sanguinegens
Prevotella_timonensis
Prevotella_sp.
BVAB1
Porphyromonas_uenonis
Megasphaera_genomosp.
Ruminococcus_lactaris
Prevotella_bivia
Megasphaera_micronuciformis
Prevotella_buccalis
other
UAB116
Relative abundance
1.0
0.8
0.6
0.4
0.2
0.0
MG
MT
1
2
3
4
5
Timepoint

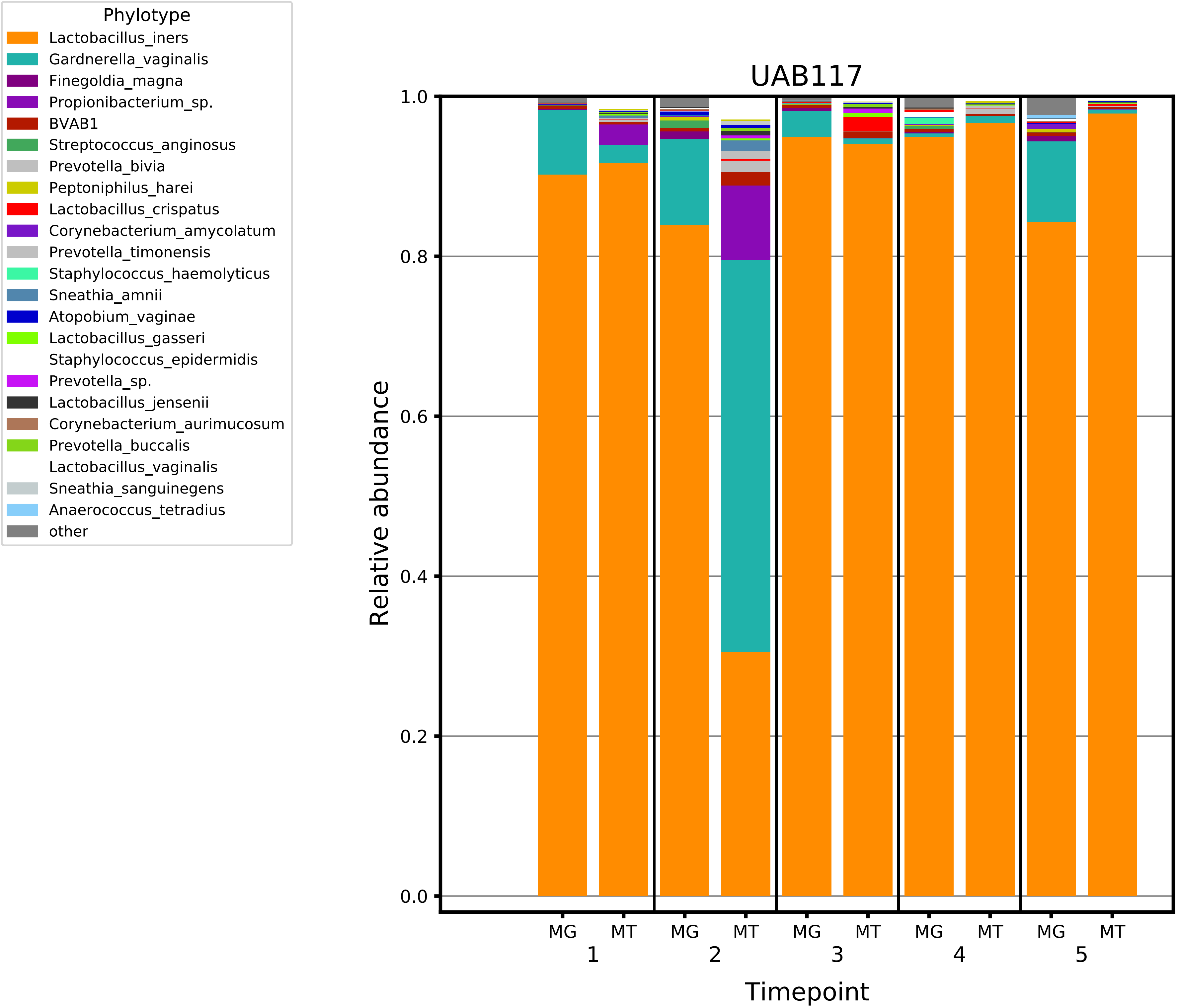
Phylotype
Lactobacillus_iners
Gardnerella_vaginalis
Finegoldia_magna
Propionibacterium_sp.
BVAB1
Streptococcus_anginosus
Prevotella_bivia
Peptoniphilus_harei
Lactobacillus_crispatus
Corynebacterium_amycolatum
Prevotella_timonensis
Staphylococcus_haemolyticus
Sneathia_amnii
Atopobium_vaginae
Lactobacillus_gasseri
Staphylococcus_epidermidis
Prevotella_sp.
Lactobacillus_jensenii
Corynebacterium_aurimucosum
Prevotella_buccalis
Lactobacillus_vaginalis
Sneathia_sanguinegens
Anaerococcus_tetradius
other
UAB117
Relative abundance
1.0
0.8
0.6
0.4
0.2
0.0
MG
MT
1
2
3
4
5
Timepoint

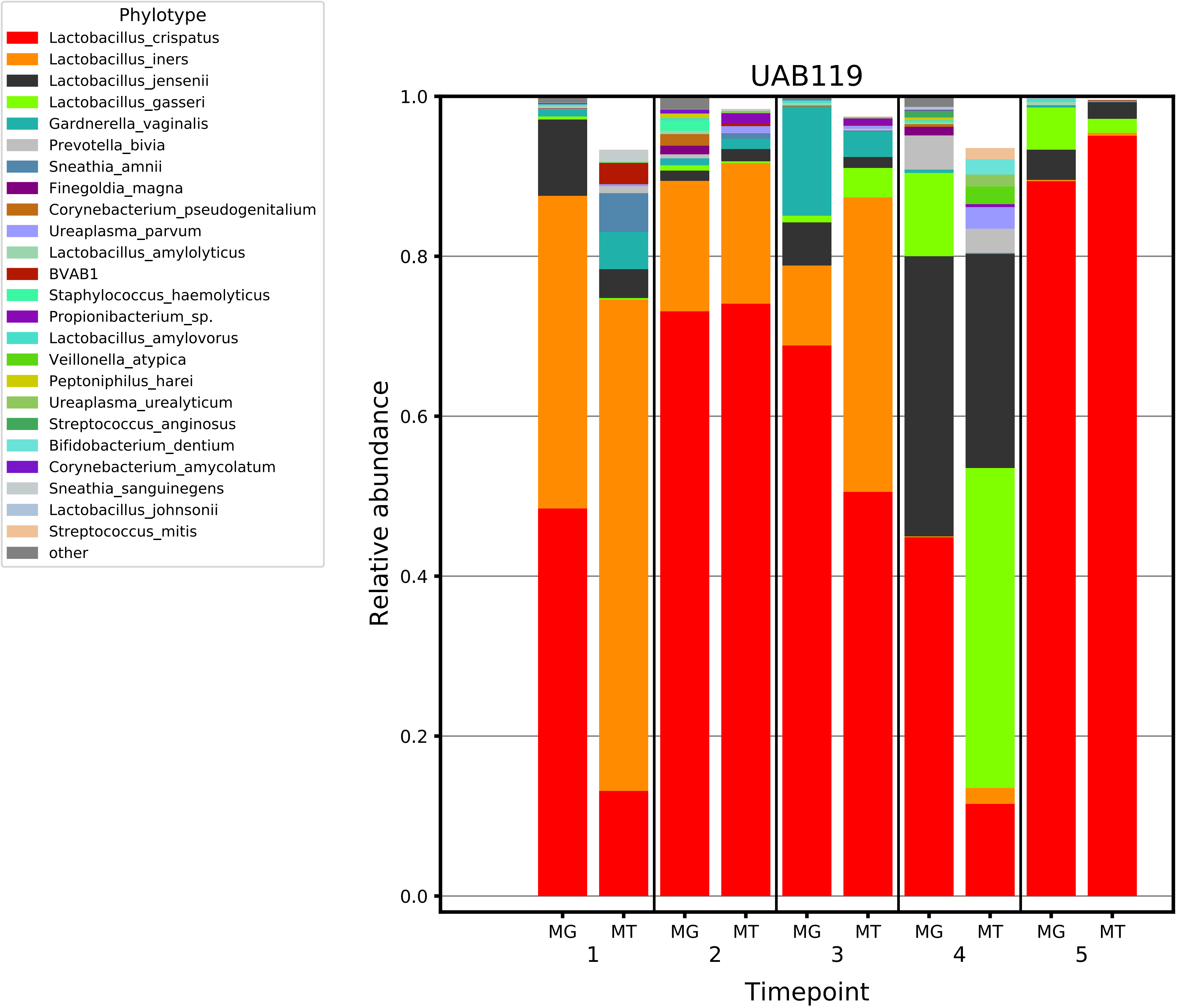

Phylotype
Lactobacillus_crispatus
Lactobacillus_iners
Lactobacillus_jensenii
Lactobacillus_gasseri
Gardnerella_vaginalis
Prevotella_bivia
Sneathia_amnii
Finegoldia_magna
Corynebacterium_pseudogenitalium
Ureaplasma_parvum
Lactobacillus_amylolyticus
BVAB1
Staphylococcus_haemolyticus
Propionibacterium_sp.
Lactobacillus_amylovorus
Veillonella_atypica
Peptoniphilus_harei
Ureaplasma_urealyticum
Streptococcus_anginosus
Bifidobacterium_dentium
Corynebacterium_amycolatum
Sneathia_sanguinegens
Lactobacillus_johnsonii
Streptococcus_mitis
other
UAB119
Relative abundance
1.0
0.8
0.6
0.4
0.2
0.0
MG MT MG MT MG MT MG MT MG MT
1 2 3 4 5
Timepoint

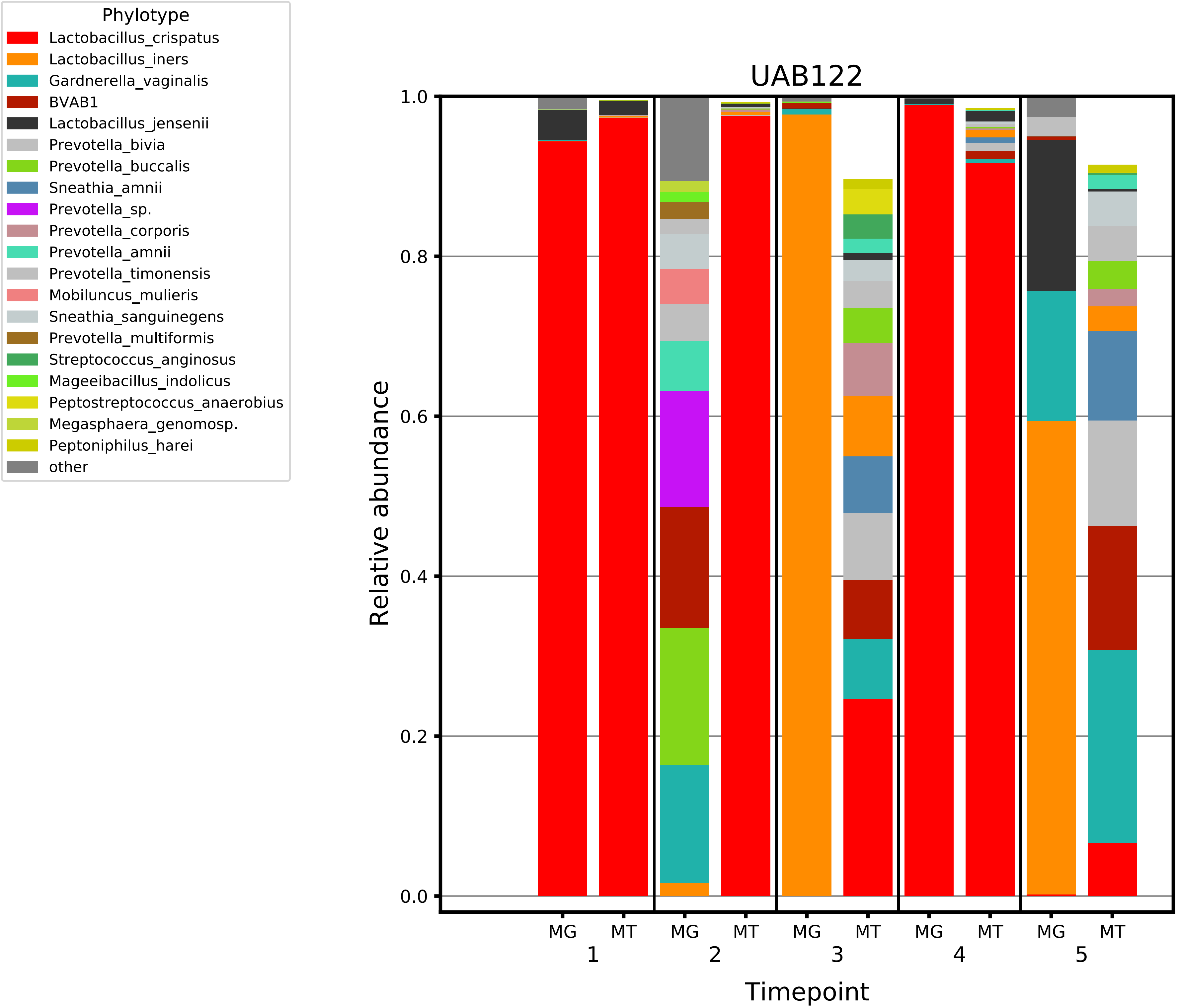

Phylotype
Lactobacillus_crispatus
Lactobacillus_iners
Gardnerella_vaginalis
BVAB1
Lactobacillus_jensenii
Prevotella_bivia
Prevotella_buccalis
Sneathia_amnii
Prevotella_sp.
Prevotella_corporis
Prevotella_amnii
Prevotella_timonensis
Mobiluncus_mulieris
Sneathia_sanguinegens
Prevotella_multiformis
Streptococcus_anginosus
Mageeibacillus_indolicus
Peptostreptococcus_anaerobius
Megasphaera_genomosp.
Peptoniphilus_harei
other
UAB122
Relative abundance
1.0
0.8
0.6
0.4
0.2
0.0
MG
MT
1
2
3
4
5
Timepoint

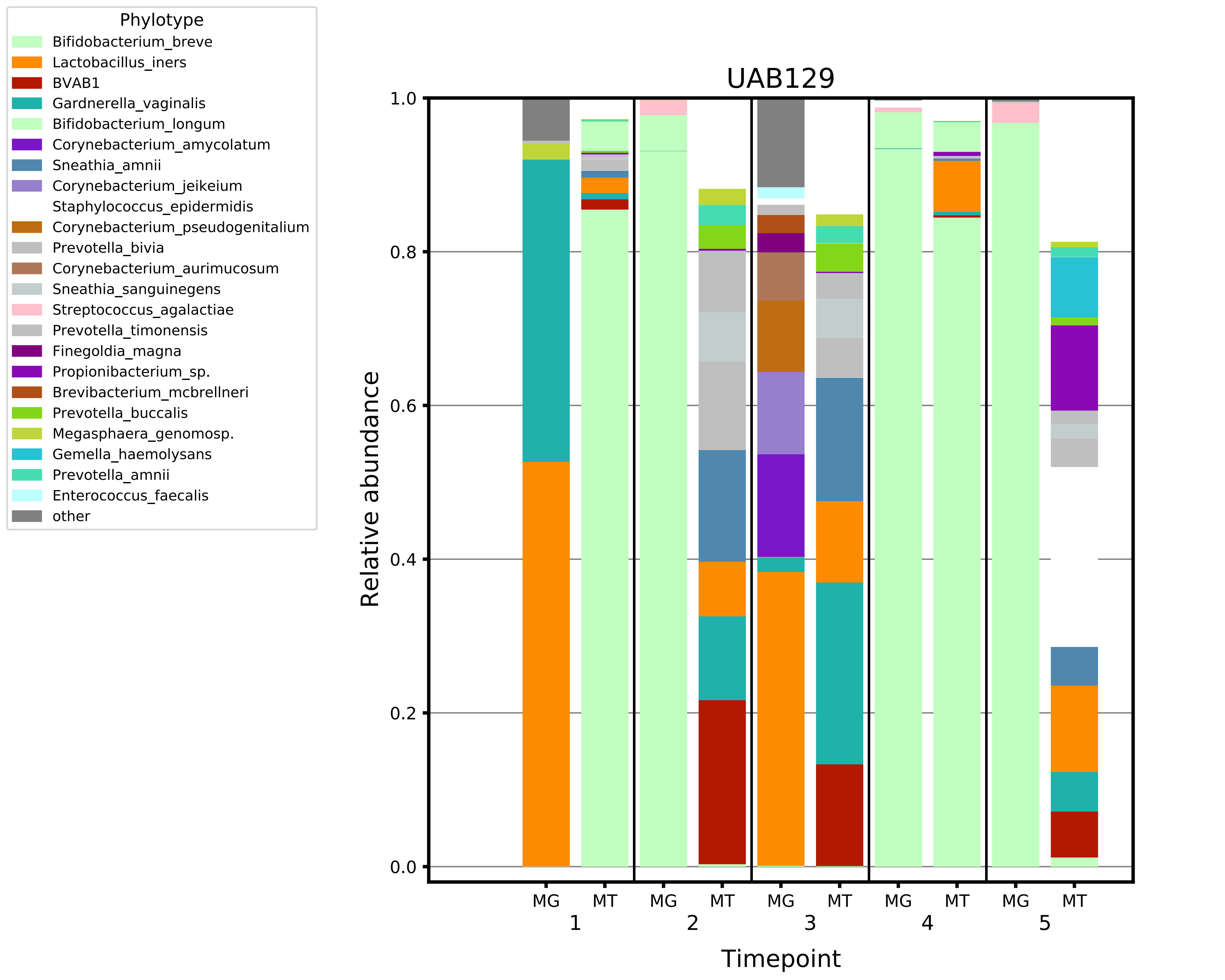
Phylotype
Bifidobacterium_breve
Lactobacillus_iners
BVAB1
Gardnerella_vaginalis
Bifidobacterium_longum
Corynebacterium_amycolatum
Sneathia_amnii
Corynebacterium_jeikeium
Staphylococcus_epidermidis
Corynebacterium_pseudogenitalium
Prevotella_bivia
Corynebacterium_aurimucosum
Sneathia_sanguinegens
Streptococcus_agalactiae
Prevotella_timonensis
Finegoldia_magna
Propionibacterium_sp.
Brevibacterium_mcbrellneri
Prevotella_buccalis
Megasphaera_genomosp.
Gemella_haemolysans
Prevotella_amnii
Enterococcus_faecalis
other
UAB129
Relative abundance
1.0
0.8
0.6
0.4
0.2
0.0
MG
MT
1
2
3
4
5
Timepoint
