# Supplementary material for: Insight into the ecology of vaginal bacteria through integrative analyses of metagenomic and metatranscriptomic data
Source: Genome Biol. 2022 Mar 1;23:66. doi: 10.1186/s13059-022-02635-9 (PMC8886902; doi:10.1186/s13059-022-02635-9)
Supplement: Supplementary file 2 — Additional file 2: Supplementary Figure 1. Linear relationship of the log10 relative expression of each taxon in relation to its log10 relative abundance. Vertical gray lines represent the X intercept of the fit, representing the relative abundance at which each taxon transitions from over- to under-expressive. Supplementary Figure 2. Scatter plot of correlated canonical variables identified in the analysis of L. crispatus (a) L. iners (b) and Gardnerella (c) gene expression. Supplemental Figure 3. Scatter plot of correlated canonical variables identified in the analysis of L. crispatus (a) L. iners (b) and Gardnerella (c) gene expression. [file 13059_2022_2635_MOESM2_ESM.docx]

# Supplemental information

##

## Supplemental Figure 1

Linear relationship of the log_10_ relative expression of each taxa in relation to its log_10_ relative abundance. Vertical gray lines represent the X intercept of the fit, representing the relative abundance at which each taxon transitions from over- to under-expressive.

**Supplemental Figure 2**

Subject level timeseries representation of glycogen and mucin degradation enzyme expression. Data points from the same subject are indicated by the dotted connecting lines and are color coded according to the CST assignment for that sample.

##

## Supplemental Figure 3

Scatter plot of correlated canonical variables identified in the analysis of *L. crispatus* (a) *L. iners* (b) and *Gardnerella* (c) gene expression.
