# Supplementary material for: Insight into the ecology of vaginal bacteria through integrative analyses of metagenomic and metatranscriptomic data
Source: Genome Biol. 2022 Mar 1;23:66. doi: 10.1186/s13059-022-02635-9 (PMC8886902; doi:10.1186/s13059-022-02635-9)
Supplement: Supplementary file 7 — Additional file 7. Review history. [file 13059_2022_2635_MOESM7_ESM.docx]

**1^st^ round**

**Reviewer 1**

This manuscript by France et al. examines the association between the vaginal microbiome's functional potential and transcriptional profile. They use a paired analysis integrating shotgun metagenomics and meta-transcriptomics and leverage access to a unique database for annotation. The data show a discordance between abundance of DNA and RNA reads where some species contribute less or more to the transcriptional landscape of the community than the DNA read abundance would suggest. These studies highlight the importance of studying microbial communities beyond the level of DNA. The text is rather difficult to navigate and there are some areas that should be clarified to enhance impact of the work presented.

Specific concerns:

1. The text should be carefully read to address numerous typographical and grammatical errors - The text is rather verbose in some sections (especially the second half of the results), strongly suggest condensing the results section to make it more succinct

2. First section of the results should be removed. It is repetitive of the information provided in the methods and materials section

3. The authors need to provide a detailed description of how "relative activity" was calculated

4. In figure 1 some paired genomic/transcriptomic samples have zero shared taxa. This happens throughout the data set but the sample closet to the taxonomy key is a good example. It is difficult to imagine microbes being completely undetectable at the DNA level but accounting for the entire transcriptome. How do the authors explain this, and could it potentially be due to the depth/annotation concerns noted below

5. For both metagenomic and metatransciptomic analysis the depth of sequencing post cleanup is highly variable with some samples range for some samples is concerning, 660K for metagenomic and 300K for transcriptomic is concerning. Additionally, it is unclear how much of these cleaned reads are annotated using the VIRGO database. It would be important to elaborate on the number of reads annotated in the final analysis.

6. It is also important to show that sequencing depth is not the reason for the discordant results. The possibility that samples with low sequencing depth/ poor annotation have a higher probability of differential metagenome/transcriptome should be eliminated to strengthen the conclusion of the study.

7. One of the initial results of this study show that a samples metagenomic CST classification is not always reflective of the metatrasctriptomic CST. However, the authors use metagenomic CST classifications for their differential expression analysis. For example, when the author do DEG analysis between CST1 and CST3 they are including samples that would be classified as ¬different CST at the transcriptome level. Could this be leading to some of the authors conclusions such as "In contrast, functions that are enriched in CST III compared to CST I do not primarily originate from L. iners, the species which defines CST III. Instead, the expression of these functions primarily originated from species like G. vaginalis, Bifidobacterium breve, and several Prevotella spp. This should be addressed in the discussion and potentially by carrying out a new classification based on transcriptional profiles.

8. Panels in Figure 3 are mislabeled making following this section of the results very challenging

a. Please add number of differentially abundant KEGG in figure 3A, C, E

b. Several examples are provided in the results section to highlight KEGG terms that are enriched in one CST versus another - however these examples are not highlighted in the figure - e.g., D-lactate dehydrogenase

c. It is near impossible to appreciate the number of KEGG pathways that are comparable at the metagenomics level

d. KEGG enrichment of the shotgun metagenomics data should be provided

e. Figure 3 would be far more informative if rather than indicating number of KEGG pathways that meet a certain FC, examples of thes pathways could be provided

9. In the methods for Shotgun metagenomic the author state that "MagAttract PowerMicrobiome DNA/RNA Kit" was used. This kit can extract both DNA and RNA from the same sample. Why was the RNA from this extraction not used? The authors should briefly discuss how using two different extraction methods/storage/handling for the DNA and RNA extraction may have impacted the results.

Minor concerns:

The image quality of figures 3 and 4 are extremely poor making them hard to interpret. I realize that figures with so many points were likely too large to upload as a PDF and this probably led to the low quality PNG's. This just needs to be fixed prior to publication.

**Reviewer 2**

France et al. studied the vaginal microbiome of 39 women using metagenomic shotgun sequencing and metatranscriptome, and found some mismatch between transcriptome and metagenome.

The results make sense, as G. vaginae (Bifidobacterium vaginae) does not mean bacterial vaginosis without other bacteria. S. amnii and S. sanguinegens showed a metatranscriptome/metagenome ratio of higher than 1.

Major issues:

1. The swab samples were resuspended using Ames transport medium, which was designed for a few pathogens. Can the authors demonstrate its lack of bias for a metagenomic community, using mock and real samples?

For the metatranscriptome, please report the percentile of ribosomal RNA before and after RiboZero.

2. Could the authors estimate the detection limit for the metatranscriptome and the metagenome here?

Page 33, line 53. "per metagenomic dataset was 2.2*10^7 (6.6*10^5-9.9*10^7)", and 1.4*10^7 per metatranscriptomic dataset (3.0*10^5-2.0*10^8). If the lowest number of reads for one sample is 6.6*10^5, a taxon whose relative abundance is less than 6.6*10^-5 will be covered by less than one sequencing read. In that case, the authors had better filter out all bacteria or KEGG pathways with a relative abundance <10-4.

The sequencing amount was even more variable for the metatranscriptome.

That would of course also impact the number of zeros (sampling zero instead of structural zero), and influence the metatranscriptome/metagenome ratio that is reported throughout the manuscript.

These are all relative, and some (RT-)qPCR results should be presented, to get a sense of the variation among samples.

3. Page 34, line 53. "the taxa as a categorical predictor". What is the cutoff used to transform a continue abundance to presence/absence?

A continuous variable is preferred, and information is lost in the transformation to a categorical variable.

4. For the metagenomics, do the authors observe a difference in the percentile of human sequences for the different vagino-types? E.g. A higher host rate with the more inflammatory bacteria.

5. With iHMP and earlier studies emphasizing an ethnic difference, do the authors think the 24 African American and the 10 Caucasian samples are similar or different here? What metadata are available? E.g. age, BMI, pregnancy history, hormones, menstrual cycle.

6. Most figures should show longitudinal data for each individual, instead of aggregated plots of all data points.

7. Growth rates can be estimated from metagenomic data. How does that compare with metatranscriptome, especially regarding longitudinal predictions?

8. Bearing in mind the compositional nature of relative abundance data (CLR transformation used here), how do the microbial networks look like for the metatranscriptome and the metagenome?

I don't find the middle part of Fig. 2 so useful, if the trend is the same for all the 6 species shown (and more in Sup. Fig. 1).

9. It is also surprising that there is no viral or fungi results here. These would at least show up with MetaPhylAn. What percentage of sequencing data in each sample is represented by the taxonomic method used in this study?

10. In fig. 1, the relative abundance of L. crispatus in sample 7 was almost 100%, but the metatranscriptome in this sample showed almost 100% relative contribution of L. iners? This is unheard of, so please double-check, and clearly show the paired omics and the time sequence for each data entry in a table.

11. Page 35, line 10. Generalized Lotka-Volterra model would be better here. It detrends the time series.

12. Page 37, line 12. "Final principal components analysis of the correlation VOGs". The loading result from sCCA was already the maximized correlation between two datasets and orthogonality. There is no need to do a second PCA on the loadings. What do the authors think this second PCA could do?

As noted above, both are compositional data, and CLR transformation was only performed for the metagenomic data, not the metatranscriptome data. Other method would be needed to take account the composition effect of both datasets (like mmvec).

13. In addition to the species networks, KO results such as D-lactate, mucin degradation (the enzymes in Fig. 5) can also potentially be shown as networks.

Minor issues:

1. The term 'ecology' is overused in microbiota studies. A more appropriate title is needed, e.g. Metatranscriptome reveals potential interplay between vaginal bacteria.

2. As it is, the metatranscriptome results seems more useful for diagnostics, if validated in more samples. But the Abstract emphasizes management of the vaginal ecosystem.

3. Lack of reference to the existing literature, e.g. L. iners coexists with other bacteria.

4. Please clearly state the inclusion & exclusion criteria.

5. Please make sure the raw data, host metadata, and codes are available. Page 32, "PRJXXXXXX", "(github.com/ravel-lab/XXXX)".

6. Each figure legend should begin with a more conclusive title

7. Fig. 1. The abbreviation "Rel." in the Y-axis of the metatranscriptome is unexplained, and should rather be spelled out as 'relative'. The figure legend in Page 38, function instead of 'fuction'.

'up to five time points' is imprecise, and should be clearly presented in figures and tables.

8. Fig. 3. Mismatch between panel labels A,C,E and legend A-C. Unexplained abbreviation in the Y-axis. 'DE' means differentially expressed?

9. Fig. 4. Please clearly mark the color code, which is positive and which is negative.

10. Fig. 5. Undefined asterisks, which presumably correspond to p-values.

11. Please be more concise throughout the manuscript. For example, we do not need a whole paragraph on Page 9 to explain Fig. 2H, if the individual trajectories, instead of an aggregated scatterplot, are presented in the figure. Also, by 'expressive', the authors probably mean under- or over-expressed. But that would not be precise either, and should rather be 'greater than x'.

12. Page 8, line 15, G. vaginalis was one of the bacteria that tend to be under-expressed (Fig. 2A). Page 9, line 9, G. vaginalis was one of the bacteria that 'maintain over expression when common' (Fig. 2B-D), which needs to have clear numbers for the transcriptome 'over expression' and the presumably metagenome 'common'.

13. Page 33, line 58. "Bowtie (85) using VIRGO default settings". Do you mean Bowtie2 here? Please verified the version.

14. Page 34, line 15. Please clarify you hmmsearch parameter and cutoff for CAZy database annotation.

15. Page 34, line 55. What does "their interaction" means? Are the results robust against sequencing depth (high-quality non-host reads)?

16. Page 35, line 14. Please clarify the number of tests here. Multiple testing should be adjusted for across taxa.

17. Page 35, line 40. Please add the r package name, "variancePartition".

18. Please use past tense in describing results. For example, in page 9, "maintain" in line 9，"transition" in line 14，and "are" in line 19 should all be past tense.

**Reviewer 3**

With pleasure, I read the study titled "Insight into the ecology of vaginal bacteria through integrative analyses of metagenomic and metatranscriptomic data" by France et al. In this study, the authors analyzed longitudinal vaginal swabs from 39 healthy women (180-195 samples total) by integrating metagenomic and transcriptomic datasets. This study resulted in several findings critical to advancing our understanding of the ecology of the vaginal microbiome in health. One important (but not completely surprising) finding is that relative abundance does not reflect transcription/activity of an organism and that transcriptional activity is dependent on co-colonizers. This was particularly evident with Sneathia amnii and Streptococcus sanguinens, two taxa that are present at low relative abundance but had disproportionately high levels of activity/gene expression. This could be relevant in susceptibility to BV or infection. The authors then focused on transcription of potential carbon and energy sources in the prevalent Community State Types (CSTs) present in their population and demonstrated that mucin degradation potential is higher in CST IV, which is non-Lactobacillus dominant. Finally, they focused on transcriptional activity of L. iners, L. crispatus, and G. vaginalis. These analyses importantly demonstrate that gene regulation is dependent on community composition. Overall, this was an exciting and robust study that advances our understanding of the ecology of the vaginal microbiota. I have a few minor critiques:

There is an initial focus on the longitudinal nature of collections, but longitudinal analyses are somewhat lacking. Fig 2H/I are the only figures that use the longitudinal design to demonstrate that expression levels correlate with future relative abundances.

It's not clear how repeated measures are accounted for or leveraged in the remaining analyses (other than subject being included as a random factor in the LMM), specifically Fig 4-6. E.g. it could be important to know how mucin/glycogen metabolism potential fluctuates over time.

It could be important to determine how an individual's vaginal microbiome ecology or metabolism potential (for mucin/glycogen) changes over time, if the relative expression score within an individual associates with lifestyle/behavioral information that the authors collected. This is mentioned in the methods but not incorporated into any analyses.

**Authors’ response**

This manuscript by France et al. examines the association between the vaginal microbiome's functional potential and transcriptional profile. They use a paired analysis integrating shotgun metagenomics and meta-transcriptomics and leverage access to a unique database for annotation. The data show a discordance between abundance of DNA and RNA reads where some species contribute less or more to the transcriptional landscape of the community than the DNA read abundance would suggest. These studies highlight the importance of studying microbial communities beyond the level of DNA. The text is rather difficult to navigate and there are some areas that should be clarified to enhance impact of the work presented.

Specific concerns:

1.The text should be carefully read to address numerous typographical and grammatical errors - The text is rather verbose in some sections (especially the second half of the results), strongly suggest condensing the results section to make it more succinct

We corrected typographical and grammatical errors. As suggested, we have reduced the length of the Results section and made it more concise. The changes are too numerous to list here and are highlighted using track change in the attached revised manuscript.

2. First section of the results should be removed. It is repetitive of the information provided in the methods and materials section

While we agree with the reviewer that this information is partly repeated in the methods section, we note that in this journal the methods are described near the end of the manuscript. We think that a brief overview of the methods provides a more natural transition from the introduction to the results. Nevertheless, we have pared down this paragraph and moved it into the next section of the results.

Line 89-97 now reads: “We characterized the vaginal microbiota of 39 North American reproductive age women (age range: 19-45) at five timepoints, spaced 2 weeks apart. Subjects included in the study self-identified as either Black or African American (n=24), White or Caucasian (n=10), Hispanic or Latino (n=4), and Asian (n=1). The metagenomes (n=195) and metatranscriptomes (n=180) of these samples were sequenced and the resulting reads were mapped to VIRGO, a non-redundant and comprehensive gene catalog of the vaginal microbiome (34) to determine the taxonomic composition of both the metagenome and metatranscriptome (Figure 1). The composition of a metagenome represents the relative abundances of taxa, whereas the composition of the metatranscriptome represents the relative activities of taxa (Supplemental Table 1).”

3. The authors need to provide a detailed description of how "relative activity" was calculated

It appears that in our descriptions of how relative activity was calculated we erred in using the terms “relative contribution” and “relative transcriptional activities. We have corrected these mistakes in the manuscript. The complete description appears in the methods and in the results.

Line 92-97: “The metagenomes (n=195) and metatranscriptomes (n=180) of these samples were sequenced and the resulting reads were mapped to VIRGO, a nonredundant and comprehensive gene catalog of the vaginal microbiome (34) to determine the taxonomic composition of both the metagenome and metatranscriptome (Figure 1). The composition of a metagenome represents the relative abundances of taxa, whereas the composition of the metatranscriptome represents the relative activities of taxa (Supplemental Table 1).”

Line 619-623: “We used the VIRGO mapping results to define the composition and structure of our metagenomic and metatranscriptomic data (Supplemental Table 1). The relative abundance of each taxon was determined by first correcting by dividing the number of mapped reads per gene by its length. These estimates of coverage were combined for each species and divided by sample total coverage in order to compute the relative abundances/activities.”

4. In figure 1 some paired genomic/transcriptomic samples have zero shared taxa. This happens throughout the data set but the sample closet to the taxonomy key is a good example. It is difficult to imagine microbes being completely undetectable at the DNA level but accounting for the entire transcriptome. How do the authors explain this, and could it potentially be due to the depth/annotation concerns noted below

While it may appear that some paired genomic and transcriptomic data have zero shared taxa that is almost always not the case. It merely appears that way because the stacked barplots are not able to graphically display taxa that have low representation in the metagenomic or metatranscriptomic data. In Supplemental Table 1 we provide the complete taxonomic table for both the metagenomic and metatranscriptomic data.

Here it can be seen that in most cases, the taxon was detected in both datasets, but in different proportions. Our observation that some species over or under contribute to the metatranscriptome is further supported by our demonstration that these differences are associated with increases or decreases in the relative abundance of the taxon at the next timepoint. We also note that detection of a bacterium’s DNA does not demonstrate their viability.

5. For both metagenomic and metatransciptomic analysis the depth of sequencing post cleanup is highly variable with some samples range for some samples is concerning, 660K for metagenomic and 300K for transcriptomic is concerning. Additionally, it is unclear how much of these cleaned reads are annotated using the VIRGO database. It would be important to elaborate on the number of reads annotated in the final analysis.

We appreciate that there are some disparities in the number of post-preprocessing reads among our metagenomic (MG) and metatranscriptomic (MT) sequence datasets. The number of reads sequenced for each MG and MT was fairly even, but the percent of human and residual rRNA reads were not predictable. Sequence data for only one metagenome and four metatranscriptomes contained less than 1 million postprocessing reads. We have added a supplemental table (Supplemental Table 4) that shows the number of post-processing reads available for each metagenome and metatranscriptome. In this table we have also indicated the percent of reads that we were able to map to the VIRGO contig database. On average, we were able to map 96.2% of the metagenomic reads and 91.5% of the metatranscriptomic reads. We have conducted some sensitivity analyses wherein we filtered out these lower read count samples and haven’t found significant disparities in the results. Consequently, we have elected to leave in the analyses that were done using the full dataset.

6. It is also important to show that sequencing depth is not the reason for the discordant results. The possibility that samples with low sequencing depth/ poor annotation have a higher probability of differential metagenome/transcriptome should be eliminated to strengthen the conclusion of the study.

We agree with the reviewer that samples that yielded fewer reads could contribute to the observed discordance our assessment of the relative abundances/contributions of taxa to the metagenome/metatranscriptome. For this reason, we reran our statistical model twice iusing 1 million and 5 million read count thresholds for the metagenomic and metatranscriptomic data. Of the 180 samples for which we had both metagenomic (MG) and metatranscriptomic (MT) data, only 4 samples had either MG or MT data that did not meet the 1 million read threshold and 45 that did not meet the 5 million read threshold. Results from the GLM did not differ when using these reduced data sets (all model terms remain significant). We therefore elected to leave the full analysis and associated figures in the manuscript but have added mention of this sensitivity analysis in the methods.

Line 637-641: “To demonstrate that these results were not affected by samples that yielded fewer reads we also conducted the analysis using datasets which contained only samples whose metagenomic and metatranscriptomic datasets contained at least 1 million microbial reads (n=176) and at least 5 million microbial reads (n=135). Results originating from the analysis of these filtered datasets did not differ from those obtained using the full dataset.”

7. One of the initial results of this study show that a samples metagenomic CST classification is not always reflective of the metatrasctriptomic CST. However, the authors use metagenomic CST classifications for their differential expression analysis. For example, when the author do DEG analysis between CST1 and CST3 they are including samples that would be classified as different CST at the transcriptome level. Could this be leading to some of the authors conclusions such as "In contrast, functions that are enriched in CST III compared to CST I do not primarily originate from L. iners, the species which defines CST III. Instead, the expression of these functions primarily originated from species like G. vaginalis, Bifidobacterium breve, and several Prevotella spp. This should be addressed in the discussion and potentially by carrying out a new classification based on transcriptional profiles.

We are glad the reviewer made note of this as we initially considered doing as the reviewer suggested. In the end we decided against the approach suggested by the reviewer wherein the transcriptome is used to define the CST and these assignments used to assess functional differences. This is because it was not clear to us how the transcriptome fit into the definition of CST, which are intended to reflect the composition of the community. To our knowledge, no one has validated the application of the CST framework to transcriptomic data. We don’t see a reason why this wouldn’t work, however, in this light the CST would have a completely different definition. It would no longer reflect the composition of the community but rather the activity of the community.

8. Panels in Figure 3 are mislabeled making following this section of the results very challenging

a. Please add number of differentially abundant KEGG in figure 3A, C, E

b. Several examples are provided in the results section to highlight KEGG terms that are enriched in one CST versus another - however these examples are not highlighted in the figure - e.g., D-lactate dehydrogenase

c. It is near impossible to appreciate the number of KEGG pathways that are comparable at the metagenomics level

d. KEGG enrichment of the shotgun metagenomics data should be provided

e. Figure 3 would be far more informative if rather than indicating number of KEGG pathways that meet a certain FC, examples of thes pathways could be provided

We thank the reviewer for pointing out the mistakes with figure 3 and have corrected them. Labels of example KEGG orthologs which were called out in the main text have been added to the figure. We also note that the complete table of FC and p-values for all of the comparisons (MG and MT) are available in Supplemental Table 2.

9. In the methods for Shotgun metagenomic the author state that "MagAttract PowerMicrobiome DNA/RNA Kit" was used. This kit can extract both DNA and RNA from the same sample. Why was the RNA from this extraction not used? The authors should briefly discuss how using two different extraction methods/storage/handling for the DNA and RNA extraction may have impacted the results.

RNA yield from the MagAttract Power Microbiome kit was found to be of insufficient quality and quantity for downstream rRNA removal and cDNA synthesis. It could be that variations in extraction procedures contribute to the observed difference, however we think it unlikely to be the primary explanatory factor. This is because the degree to which a taxon was over- or under- expressed was found to be predictive of its change in relative abundance over a two week time period. As suggested we have added this point to the discussion.

Line 336-339: “It could be that variation in the techniques used to extract the DNA and RNA contributed to the observed differences. However, we think it is unlikely in this case because the differences between the composition of the metagenome and metatranscriptome were found to be predictive of community dynamics.”

Minor concerns:

The image quality of figures 3 and 4 are extremely poor making them hard to interpret. I realize that figures with so many points were likely too large to upload as a PDF and this probably led to the low quality PNG's. This just needs to be fixed prior to publication.

We apologize for the poor figure resolutions. This was indeed caused by the required use of PNGs in the submission. We have vector PDFs available for all of the figures.

Reviewer #2:

France et al. studied the vaginal microbiome of 39 women using metagenomic shotgun sequencing and metatranscriptome, and found some mismatch between transcriptome and metagenome. The results make sense, as G. vaginae (Bifidobacterium vaginae) does not mean bacterial vaginosis without other bacteria. S. amnii and S. sanguinegens showed a metatranscriptome/metagenome ratio of higher than 1.

Major issues:

1. The swab samples were resuspended using Ames transport medium, which was designed for a few pathogens. Can the authors demonstrate its lack of bias for a metagenomic community, using mock and real samples?

For the metatranscriptome, please report the percentile of ribosomal RNA before and after RiboZero.

Liquid Amies transport media has been previously validated for storage of vaginal swab specimens for use in microbiome studies (Bai et al, Comparison of Storage Conditions for Human Vaginal Microbiome Studies, 2012 PLoS One). We have added mention of the residual percent rRNA following RiboZero reduction in the methods. We did not sequence any metatranscriptomes without first using RiboZero as the samples would have been 99% or more rRNA.

Line 604-605: “Host-removed metatranscriptomic sequence datasets contained, on average, 31.7% residual rRNA sequences.”

2. Could the authors estimate the detection limit for the metatranscriptome and the metagenome here?

Page 33, line 53. "per metagenomic dataset was 2.2*10^7 (6.6*10^5-9.9*10^7)", and 1.4*10^7 per metatranscriptomic dataset (3.0*10^5-2.0*10^8). If the lowest number of reads for one sample is 6.6*10^5, a taxon whose relative abundance is less than 6.6*10^-5 will be covered by less than one sequencing read. In that case, the authors had better filter out all bacteria or KEGG pathways with a relative abundance <10-4. The sequencing amount was even more variable for the metatranscriptome. That would of course also impact the number of zeros (sampling zero instead of structural zero), and influence the metatranscriptome/metagenome ratio that is reported throughout the manuscript. These are all relative, and some (RT-)qPCR results should be presented, to get a sense of the variation among samples.

We have added a supplemental table that describes the number of post-and preprocessing reads for each metagenome and metatranscriptome and the percentage of these reads that map to VIRGO contigs. It is true that several of the samples are on the lower end of sequence depth (1 MG and 4 MT with <1million reads). There are many different approaches to dealing with variation in sequencing depth for sequence datasets. Subsampling to the lowest common denominator, as suggested by the reviewer, has not proven to be useful as it eliminates useful data and also introduces too many zeros as mentioned by the reviewer. We took the approach of performing a sensitivity analysis using filtered datasets that do not include low read count samples (in this instance thresholds of 1 million and 5 million reads). Our results did not change when these low read count samples were omitted and therefore we elected to keep the analysis that used the full dataset.

We interpret the reviewer’s comment on RT-qPCR to be a suggestion that there might be differences in the numbers of bacteria that contribute to differences in sequencing depth. Differences in sequencing depth primarily originate from variation in the percent of host DNA and RNA in the sample as well as the percent of residual rRNA (in the case of metatranscriptomic data). It could be that this results from differences in the absolute abundance of bacteria in the community contribute to the percent host DNA and RNA. We do have qPCR census estimates for bacterial communities in a majority of these samples. There is variation in our estimates for the number of 16S rRNA gene copies per swab but these values do not correlate with differences in sequence depth. (See figure below).

3. Page 34, line 53. "the taxa as a categorical predictor". What is the cutoff used to transform a continue abundance to presence/absence? A continuous variable is preferred, and information is lost in the transformation to a categorical variable.

In this section of the methods we described the statistical approach used to model the relationship between the abundance of a taxon and its relative expression. We do not transform a taxon abundance into presence/absence scores. As laid out in the text, the model includes the abundance data (log10 transformed, continuous), the taxa (categorical), and their interaction(s) as predictor variables. The first term produces a slope value that describes the overall relationship between the abundance of taxa and the relative levels of gene expression. The second term produces a separate intercept value for each taxon and the interaction term results in separate slopes that describe taxa specific modifying effects on the overall relationship between abundance and relative expression.

4. For the metagenomics, do the authors observe a difference in the percentile of human sequences for the different vagino-types? E.g. A higher host rate with the more inflammatory bacteria.

We have previously shown that the percentage of host reads in a vaginal shotgun metagenome varies with community state type (Ma et al, A comprehensive nonredundant gene catalog reveals extensive within-community intraspecies diversity in the human vagina, 2020, Nature Com). This is the opposite of what the reviewer expected.

5. With iHMP and earlier studies emphasizing an ethnic difference, do the authors think the 24 African American and the 10 Caucasian samples are similar or different here? What metadata are available? E.g. age, BMI, pregnancy history, hormones, menstrual cycle.

We decided not to focus our analysis on the variation seen in women identifying as different ethnicities because the number of women participating in this study was too low to allow a robust comparison. This is especially true if we stratifyed by community state type, which would be necessary due to known differences in the frequencies of each community state type among women of various ethnicities.

6. Most figures should show longitudinal data for each individual, instead of aggregated plots of all data points.

We have added supplemental figures which display the data for individuals including: 1) longitudinal profiles of metagenome and metatranscriptome taxonomic composition for each subject, and 2) longitudinal expression of glycogen and mucin degradation enzymes. All of our analyses except for the sCCA account for the longitudinal nature of the data.

7. Growth rates can be estimated from metagenomic data. How does that compare with metatranscriptome, especially regarding longitudinal predictions?

While it is true that it is possible to approximate a taxa’s growth rate from metagenomics data, it has not been validated for vaginal bacteria. These analyses would require a substantial amount of additional work to assemble and bin high quality MAGs, and would require in vitro experimentation to validate the results.

8. Bearing in mind the compositional nature of relative abundance data (CLR transformation used here), how do the microbial networks look like for the metatranscriptome and the metagenome?

I don't find the middle part of Fig. 2 so useful, if the trend is the same for all the 6 species shown (and more in Sup. Fig. 1).

We did not produce microbial co-abundance networks for these data as its difficult to interpret the correlation coefficients due to the intrinsic negative correlation bias of compositional data. A future study could leverage the data produced here with in vitro and in vivo estimates of synergy/antagonism between species and with absolute abundance measures to generate a more meaningful co-abundance network.

While the slopes of each line appear to be similar for each taxon, the intercept varies between taxa. These differences are discussed in the text with regard to each taxon’s ability to dominate the vaginal microbiota.

9. It is also surprising that there is no viral or fungi results here. These would at least show up with MetaPhylAn. What percentage of sequencing data in each sample is represented by the taxonomic method used in this study?

VIRGO has some capability to identify Candida species (the most common vaginal fungi in the human vagina) in shotgun metagenomics and metatranscriptomic datasets. However, very few fungal reads were detected in these samples, precluding any analyses of the mycobiome. The maximum percent fungal reads detected in a metagenome was only 0.05% and most samples were not found to contain any fungal reads. Similarly, the maximum percent fungal reads detected in a metatranscriptome was 0.2%. There are some viral genes annotated in VIRGO however, these are primarily bacteriophage genes which are often found integrated in bacterial genomes. Over 90% of the post-preprocessing reads were able to be mapped to the VIRGO contig database, indicating that most of the species present in the samples are also present in VIRGO.

10. In fig. 1, the relative abundance of L. crispatus in sample 7 was almost 100%, but the metatranscriptome in this sample showed almost 100% relative contribution of L. iners? This is unheard of, so please double-check, and clearly show the paired omics and the time sequence for each data entry in a table.

We looked into the results for this sample and have confirmed that the correct MG and MT data are associated with it. The paired omics time series data are provided for each subject in Supplemental File 1. As can be seen in this file, this subject (AYAC03) has a high proportion of L. crispatus in the MG, but L. iners is also present at a lower relative abundance. The associated MT contains a high proportion of L. iners with a very small proportion of L. crispatus, further supporting the results presented in this study. Importantly, at the subsequent timepoint, the abundance of L. iners has increased, consistent with the predictive capacity of relative expression. It is not clear what to expect when looking at paired vaginal metagenomes and metatranscriptomes as there are relatively few published studies have collected such data. When looking at one such study (Serrano et al, Racioethnic diversity in the dynamics of the vaginal microbiome during pregnancy, 2019, Nature Medicine) discordance was also observed between these two datatypes, although perhaps not to the same extent.

11. Page 35, line 10. Generalized Lotka-Volterra model would be better here. It detrends the time series.

It is not clear to us how to implement a Generalized Lotka-Volterra model using our data given the limited number of samples we have for each subject. This approach is generally used when a large number of samples (>10) are available for a community. This threshold was demonstrated nicely in: [Joseph et al, Compositional Lotka-Volterra describes microbial dynamics in the simplex, 2020, PLoS Computational Biology]. The authors of this study investigated the effect of small samples sizes on the inference of Generalized Lotka-Volterra model parameters and found that the predictive power of the model declined when smaller sample sizes (5-10) were used or when sampling times were spaced further apart (>4 days apart). The advantage of the approach we used (linear mixed model) is that it does not require the inference of as many parameters and therefore doesn’t require as many samples per subject.

12. Page 37, line 12. "Final principal components analysis of the correlation VOGs". The loading result from sCCA was already the maximized correlation between two datasets and orthogonality. There is no need to do a second PCA on the loadings. What do the authors think this second PCA could do?

As noted above, both are compositional data, and CLR transformation was only performed for the metagenomic data, not the metatranscriptome data. Other method would be needed to take account the composition effect of both datasets (like mmvec). The final principal component analysis provides a visualization of where samples reside in a space that is defined by the correlated features identified by the sCCA. This allows the researcher to make inferences about the samples whereas the factor loadings give insight into the kinds of correlated features.

We considered using the CLR transformation on the metatranscriptomic data as well but ultimately decided against it. A major drawback of this type of log transformation is that all of the zeroes in the dataset must be inferred. In the taxonomic table, there were relatively few zeroes as only the most prevalent taxa were included in the analysis. However, the metatranscriptomic dataset contains a large number of 0s as genes are not always expressed in all samples. We chose instead to use the transcripts per million transformation which does correct for differences in library size and gene length. This approach is used frequently in RNAseq analyses and has been recommended for such data (see [Abrams et al, A protocol to evaluate RNA sequencing normalization methods, 2019, BMC Bioinformatics]).

13. In addition to the species networks, KO results such as D-lactate, mucin degradation (the enzymes in Fig. 5) can also potentially be shown as networks.

It is not clear to us what type of network the reviewer thinks we should construct using data from our functional analyses. In our experience, network diagrams with many nodes (here there are >2000) are rarely useful.

Minor issues:

1. The term 'ecology' is overused in microbiota studies. A more appropriate title is needed, e.g. Metatranscriptome reveals potential interplay between vaginal bacteria.

We can agree that ‘ecology’ is often misused in microbiome research. However in this case, we attempted integration of metatranscriptomic and metagenomic data to gain meaningful insight to the dynamics and interactions of vaginal vaginal microbiota. Given this we think “ecology” is entirely appropriate.

2. As it is, the metatranscriptome results seems more useful for diagnostics, if validated in more samples. But the Abstract emphasizes management of the vaginal ecosystem.

We appreciate the reviewer’s suggestion and have edited the abstract to include mention of the diagnostic potential of metatranscriptomic data.

Line 29-31: “Our results provide insight to the functional ecology of the vaginal microbiota, demonstrate the diagnostic potential of metatranscriptomic data, and reveal strategies for the management of these ecosystems.”

3. Lack of reference to the existing literature, e.g. L. iners coexists with other bacteria.

We have added mention of this previous observation and added references to relevant papers.

Line 458-560: “L. iners is often found to co-occur with bacteria associated with CST IV communities, including G. vaginalis (73, 74).”

4. Please clearly state the inclusion & exclusion criteria.

Inclusion exclusion criteria are have been described previously and are extensively described in the referenced text.

5. Please make sure the raw data, host metadata, and codes are available. Page 32, "PRJXXXXXX", "(github.com/ravel-lab/XXXX)".

The sequence data, metadata, and all computer code have been deposited in either SRA or Github. Accession numbers have been provided in the text.

L542-457 "Metagenomic and metatranscriptomic datasets generated as part of this study have been deposited in the NCBI Short Read Archive under the Bioprojects PRJNA208535 & PRJNA575586. Accession numbers for each individual metagenome and metatranscriptome will be found in Supplemental Table 4, upon acceptance and submission. Python and R code used in the described analyses and the generation of figures is available at: (github.com/ravel-lab/MTMG_longitudinal).”

6. Each figure legend should begin with a more conclusive title

Figure legends have been updated as suggested by the reviewer.

“Figure 1: Taxonomic composition of the vaginal microbiota of 39 women at up to five timepoints as assessed by shotgun metagenomics (n=194, panel A). Relative contribution of each species to the metatranscriptome of the corresponding samples (n=180, B). Both estimates were derived using VIRGO mapping results corrected for gene length. Samples were sorted in the same order in the two datasets. Gaps indicate the fourteen samples for which no corresponding metatranscriptomic data were available. Communities were assigned to CSTs based on their taxonomic composition using VALENCIA (9) according to the following scheme: CST I –L. crispatus dominated, CST II–L. gasseri dominated, CST III–L. iners dominated, and CST V–L. jensenii dominated) and CST IV. Separate plots that display the longitudinal data series for each individual subject can be found in Supplemental File 1.”

“Figure 2: The contribution of a species to the metatranscriptome does not always match its relative abundance. Relative gene expression in the 25 most abundant species in vaginal communities was calculated by dividing the species contribution to the metatranscriptome by its relative abundance in the metagenome (A). Relationship between a species’ relative expression and its relative abundance in a community (BG, Supplemental Figure 1).”

“Figure 3: A taxon’s relative activitiy is predictive of its change in relative abundance. Log10 fold change in the relative abundance of a species from one timepoint (T1) to the next (T2) as a function of its relative expression at T1 (A). Species-specific intercepts from this model represent the minimum relative expression requred for a species to maintain its relative abundance from T1 to T2 (B).”

“Figure 4: Functional differences in the transcriptional activity of CST and associated taxonomic drivers. Volcano plots displaying log2 fold change differences in the expression of KEGG orthologs between communities assigned to different community state types (panels A,C,and E). Points in blue represent KEGG orthologs which were identified as differentially expressed and differentially abundant. Points in yellow represent KEGG orthologs that were found to be differentially expressed but not differentially abundant. In panels B,D,and F the taxa responsible for the expression of these KEGG orthologs are displayed. For each fold change category, the relative contribution of each taxon is displayed, averaged across all of the KEGG orthologs in that category. Numbers above the bars indicate the number of differentially expressed KEGG orthologs in the category. *DE, Differentially expressed.”

“Figure 5: CST associated differences in the expression of KEGG modules. Differentially expressed KEGG orthologs were mapped to KEGG modules (represent metabolic functions). The heatmap displays the average log2 fold change values for KEGG modules that demonstrated at least a four-fold difference in one of three comparisons (CST I versus CST III; CST I versus CST IV, or CST III versus CST IV). Two columns are plotted for each of the three comparisons, with each representing KEGG modules whose orthologs demonstrated higher expression in the specified CST. Some KEGG modules included KEGG orthologs that had higher expression in each of the CSTs compared. Hierarchical clustering was performed on the fold change values using Euclidean distance and Ward linkage to separate the modules into seven categories.”

“Figure 6: Expression of mucin but not glycogen degradation related genes varied by CST. Differences in the expression of key functions related to the metabolism of glycogen and mucin, two abundant host-derived resources in the vagina. Plots on the left (panels A, C, E, G, I, K, and M) display the combined transcription of the specified enzymes in log10 transformed transcripts per million (TPM). For each enzyme class, a linear mixed model was used to test for significant differences in gene expression between CST, with subject included as a random factor. Brackets represent significant post hoc comparisons derived from these models. Plots on the right (panels B, D, F, H, J, L, and N) display the average relative contribution of various taxa to the transcription of these genes by members of each CST. Longitudinal representations of the expression levels of each enzyme class, for each subject, can be found in Supplemental Figure 2. *p<0.05; ** p<0.01; *** p<0.001.”

“Figure 7: Community composition modulates species’ gene expression. Principal component biplots of the VOGs and taxa identified as correlated by sparse canonical correlation analysis (sCCA). The analysis was conducted on the transcriptional activity of the three most prevalent species: L. crispatus (panel A), L. iners (panel B) and G. vaginalis (panel C). The large circular points in each plot represent the samples. In panel A these points are colored according to the combined log10 relative abundance the two negatively contributing taxa (A. vaginae and G. vaginalis). In panels B and C these points are colored according to the ratio of the combined relative abundances of taxa that contribute either positively and negatively to the correlation. Factor loadings of the genes (Positive, blue; Negative, magenta) and taxa (Positive, green; Negative, gold) are included in the plot. For legibility, the labels for the taxa loadings, but not the VOG loadings are displayed.”

7. Fig. 1. The abbreviation "Rel." in the Y-axis of the metatranscriptome is unexplained, and should rather be spelled out as 'relative'. The figure legend in Page 38, function instead of 'fuction'.

We have updated Figure 1 as suggested by the reviewer and have corrected the typo in the legend for Figure 2.

'up to five time points' is imprecise, and should be clearly presented in figures and

tables.

We have revised the text as suggested by the reviewer to more clearly state the number of samples for which we generated paired shotgun metagenomic and metatranscriptomic data.

Line 555-557: “Paired metagenomic and metatranscriptomic could not be generated for 15 of the samples resulting in six women with four samples, three women three samples, and one woman with two samples.”

8. Fig. 3. Mismatch between panel labels A,C,E and legend A-C. Unexplained abbreviation in the Y-axis. 'DE' means differentially expressed?

We have corrected the labeling of the subplots in now figure 4 and have updated the legend to explain the meaning of “DE”.

“Figure 4: Functional differences in the transcriptional activity of CST and associated taxonomic drivers. Volcano plots displaying log2 fold change differences in the expression of KEGG orthologs between communities assigned to different community state types (panels A,C,and E). Points in blue represent KEGG orthologs which were identified as differentially expressed and differentially abundant. Points in yellow represent KEGG orthologs that were found to be differentially expressed but not differentially abundant. In panels B,D,and F the taxa responsible for the expression of these KEGG orthologs are displayed. For each fold change category, the relative contribution of each taxon is displayed, averaged across all of the KEGG orthologs in that category. Numbers above the bars indicate the number of differentially expressed KEGG orthologs in the category. *DE, Differentially expressed.”

9. Fig. 4. Please clearly mark the color code, which is positive and which is negative.

Rather than plotting positive and negative fold changes, whose meaning is dependent on the directionality of the comparison, we have elected to split the fold changes of KEGG orthologs by their association with the CSTs compared. This results in two columns for each comparison as shown in Figure 4.

10. Fig. 5. Undefined asterisks, which presumably correspond to p-values.

We have added the omitted information regarding the significance levels in now Figure 6.

11. Please be more concise throughout the manuscript. For example, we do not need a whole paragraph on Page 9 to explain Fig. 2H, if the individual trajectories, instead of an aggregated scatterplot, are presented in the figure. Also, by 'expressive', the authors probably mean under- or over-expressed. But that would not be precise either, and should rather be 'greater than x'.

We have made some adjustments to the text of the results for the sake of brevity. We have also split Figure 2 parts H and I into a separate figure as we believe its inclusion in Figure 2 is causing some confusion. In Figure 2, parts A-G, we are displaying the relative transcriptional activity of species and its relationship to its relative abundance. In Figure 2 parts H and I (Now Figure 3 parts A and B) we are displaying how a species’ relative expression impacts its change in relative abundance over two timepoints.

12. Page 8, line 15, G. vaginalis was one of the bacteria that tend to be underexpressed (Fig. 2A). Page 9, line 9, G. vaginalis was one of the bacteria that 'maintain over expression when common' (Fig. 2B-D), which needs to have clear numbers for the transcriptome 'over expression' and the presumably metagenome 'common'.

We do not believe these two statements are in conflict. While Gardnerella is more likely to be under-expressive than over, it is also more likely to be at a high relative abundance. We have added some numbers to the text which support these statements.

Line 124-131: “The relative abundance at which this switch from over- to underexpressive occurs varied by species (Figure 2B-G; Supplemental Figure 1; linear mixed model; F24,4261=198, p<0.001). Species such as L. crispatus, L. iners, and G. vaginalis (Figure 2, panels B-D), which tend to be found at higher relative abundances,

maintain over-expression when common (x-intercept >10-2 relative abundance). Conversely, species like A. vaginae and Ca. L. vaginae (Figure 2 E and F), which tend to be found at moderate or low relative abundances, transition to under-expression at lower relative abundances (x-intercept <10-2 relative abundance).”

13. Page 33, line 58. "Bowtie (85) using VIRGO default settings". Do you mean Bowtie2 here? Please verified the version.

Bowtie (not Bowtie2) was used in the mapping of reads to the VIRGO database as it allows for precise control of the number of mismatches allowed.

14. Page 34, line 15. Please clarify you hmmsearch parameter and cutoff for CAZy database annotation.

As requested, we have added more details on the use of dbCAN2 to annotate VIRGO genes using the CAZy database.

Line 613-617: “To enable our analysis of carbohydrate catabolic enzymes, we further annotated the VIRGO database with the CAZy schema using dbCAN2 with default settings (87). Annotations where two of the three methods used by dbCAN2 (Hmmer, DIAMOND, Hotpep) were accepted, as recommended.”

15. Page 34, line 55. What does "their interaction" means? Are the results robust against sequencing depth (high-quality non-host reads)?

In the context of a linear mixed model an interaction term represents instances where the effects of two independent variables are not additive, but rather impact one another.

16. Page 35, line 14. Please clarify the number of tests here. Multiple testing should be adjusted for across taxa.

As described in the text, the post hoc comparisons were adjusted using the Tukey method.

17. Page 35, line 40. Please add the r package name, "variancePartition".

We have corrected the package name to “variancePartition”, the reference for this package already appears in the text.

Line 647-650: “We then performed library size normalization using the edgeR package (88) and then modeled the differential expression of each KEGG ortholog using the dream function from the variancePartition package in R (89).”

18. Please use past tense in describing results. For example, in page 9, "maintain" in line 9，"transition" in line 14，and "are" in line 19 should all be past tense.

We thank the reviewer for pointing this out and have gone through the results section and corrected any mistakes in verb tense.

Reviewer #3:

With pleasure, I read the study titled "Insight into the ecology of vaginal bacteria through integrative analyses of metagenomic and metatranscriptomic data" by France et al. In this study, the authors analyzed longitudinal vaginal swabs from 39 healthy women (180-195 samples total) by integrating metagenomic and transcriptomic datasets. This study resulted in several findings critical to advancing our understanding of the ecology of the vaginal microbiome in health. One important (but not completely surprising) finding is that relative abundance does not reflect transcription/activity of an organism and that transcriptional activity is dependent on co-colonizers. This was particularly evident with Sneathia amnii and Streptococcus sanguinens, two taxa that are present at low relative abundance but had disproportionately high levels of activity/gene expression. This could be relevant in susceptibility to BV or infection. The authors then focused on transcription of potential carbon and energy sources in the prevalent Community State Types (CSTs) present in their population and demonstrated that mucin degradation potential is higher in CST IV, which is non- Lactobacillus dominant. Finally, they focused on transcriptional activity of L. iners, L. crispatus, and G. vaginalis. These analyses importantly demonstrate that gene regulation is dependent on community composition. Overall, this was an exciting and robust study that advances our understanding of the ecology of the vaginal microbiota.

I have a few minor critiques:

There is an initial focus on the longitudinal nature of collections, but longitudinal analyses are somewhat lacking. Fig 2H/I are the only figures that use the longitudinal design to demonstrate that expression levels correlate with future relative abundances. It's not clear how repeated measures are accounted for or leveraged in the remaining analyses (other than subject being included as a random factor in the LMM), specifically Fig 4-6. E.g. it could be important to know how mucin/glycogen metabolism potential fluctuates over time.

It could be important to determine how an individual's vaginal microbiome ecology or metabolism potential (for mucin/glycogen) changes over time, if the relative expression score within an individual associates with lifestyle/behavioral information that the authors collected. This is mentioned in the methods but not incorporated into any analyses.

The comments from reviewer three appear to be related to one another so we have elected to provide a joint response.

As pointed out by the reviewer, the first part of the paper does focus heavily on the longitudinal aspect of the data, demonstrating the predictive power of a species’ relative expression. However, in subsequent analyses we do not investigate, in detail, the longitudinal aspect of the data, but we do correct for it using mixed effect models. It was not clear to us how to present a longitudinal analysis of the functional data in a meaningful way, as function is driven in a large part by composition (which was analyzed in the first part). Further compounding this is that each women’s longitudinal profile is fairly unique and would almost necessitate deep dives into individual time series. The reviewer also wonders on our lack of analyses of lifestyle/behavioral information. They are correct that these data were collected and are available. However, the uniqueness of each women precludes their incorporation into our analyses given the size of our dataset. We hope to perform a subsequent study leveraging these data, in combination with yet-to-be-generated data, in order to examine more carefully the association between such factors and especially our metatranscriptomic data.

We appreciate the reviewer’s suggestion of presenting the time series data for the expression of glycogen and mucin degradation genes. We have generated figures for each gene type which displays the individual time series of expression for each participant and placed them in the supplemental material. We have added mention of this figure in the text and believe its inclusion has strengthened the study.

**2^nd^ round**

**Reviewer 3**

I thank the authors for a careful revision of their manuscript per reviewer's instructions. Their revisions sufficiently address my prior comments. I have no further critiques.
